# Are Plant Species Able to Keep Pace with the Rapidly Changing Climate?

**DOI:** 10.1371/journal.pone.0067909

**Published:** 2013-07-24

**Authors:** Sarah Cunze, Felix Heydel, Oliver Tackenberg

**Affiliations:** 1 Biodiversity and Climate Research Centre Frankfurt (BiK-F), Frankfurt am Main, Germany; 2 Institute of Ecology, Evolution and Diversity, Goethe University, Frankfurt am Main, Germany; University of Kent, United Kingdom

## Abstract

Future climate change is predicted to advance faster than the postglacial warming. Migration may therefore become a key driver for future development of biodiversity and ecosystem functioning. For 140 European plant species we computed past range shifts since the last glacial maximum and future range shifts for a variety of Intergovernmental Panel on Climate Change (IPCC) scenarios and global circulation models (GCMs). Range shift rates were estimated by means of species distribution modelling (SDM). With process-based seed dispersal models we estimated species-specific migration rates for 27 dispersal modes addressing dispersal by wind (anemochory) for different wind conditions, as well as dispersal by mammals (dispersal on animal's coat – epizoochory and dispersal by animals after feeding and digestion – endozoochory) considering different animal species. Our process-based modelled migration rates generally exceeded the postglacial range shift rates indicating that the process-based models we used are capable of predicting migration rates that are in accordance with realized past migration. For most of the considered species, the modelled migration rates were considerably lower than the expected future climate change induced range shift rates. This implies that most plant species will not entirely be able to follow future climate-change-induced range shifts due to dispersal limitation. Animals with large day- and home-ranges are highly important for achieving high migration rates for many plant species, whereas anemochory is relevant for only few species.

## Introduction

Climate change is expected to have an important impact on biodiversity. Due to climate warming the potential ranges of European plant species will probably shift pole wards and to higher altitudes (e.g. [1]). Although evolutionary adaptations to warmer conditions have been documented, there is little evidence that observed genetic shifts will mitigate negative effects at the species' level [2].The impact of climate change on biodiversity and properties of ecosystems will clearly depend on the ability of plant species to migrate to new sites with suitable habitat conditions [3], [4]. Migration of plant species (i.e. a directional shift in a species' ranges, [5]) is a complex process determined by dispersal potentials, fecundity, population establishment, population growth, landscape structure, and the availability of suitable habitat [6], [7]. Future climate change is predicted to advance much faster than during post glacial times and thus higher migration rates will be necessary to follow the associated range shifts [8]. A mismatch between the rate of change in climatic habitat conditions and the ability of species to follow these changes may strongly influence ecosystem properties and processes [9]. Hence, climate change can be considered a major threat to biodiversity (e.g. [1]) especially in case of dispersal limitation. Species distribution modelling (SDM) - widely and successfully used to predict species responses to climate change (e.g. [1], [10], [11]) - mostly ignores differences between species migration potentials by assuming that migration is either not limited (full migration) or absent (no migration) [6]. For the realization of range shifts rare long distance dispersal (LDD) events are highly important [12] and thus should be taken into account for estimations of migration rates. Although there are efforts in simulating LDD, modelled migration rates are seldom implemented into SDM yet ([13] but see [14], [15], [6], [16]).

A limitation of estimating migration rates is that rarely more than a single dispersal mode is considered (e.g. [6]). [12] stresses the importance of considering total dispersal kernels that incorporate multiple dispersal modes. Specifically, dispersal by animals should be considered, as it seems to be the most efficient dispersal mode for many plant species (e.g. [17], [18]).

In order to improve our knowledge on the importance of dispersal limitation for plant migration, this study addresses the following questions:

- Are migration rates derived from process-based seed dispersal models high enough to explain the realized postglacial migration?
- Which dispersal vectors are most effective in terms of resulting in high migration rates?
- How big are the differences in migration rates between plant species?
- Will plant species be able to keep pace with a rapidly changing climate and to what extent will species be able to fulfil their potential future ranges?
- What has a greater impact on predictions of future development of biodiversity: differences in migration rates achieved by different dispersal modes or differences in climate change scenarios?

## Materials and Methods

### General approach

**Figure 1:**
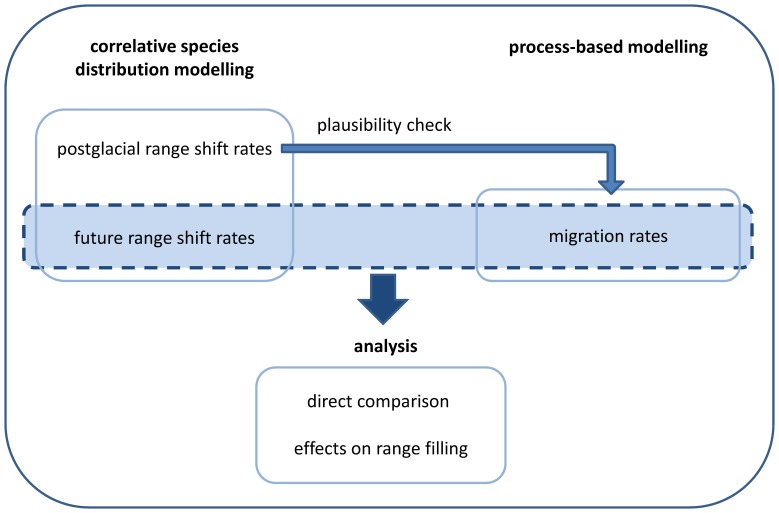
General work flow of the study: We compared the modelled potential future range shift rates and the modelled migration rates. Future range shift rates can be seen as a measure of the distances that are required to be covered per year and the migration rates as a measure for the distances that can be covered by migration per year by plant species. The future range shifts were modelled by means of species distribution modelling (SDM), considering nine different environmental models for 2080. The migration rates were modelled by means of process-based models considering 27 different dispersal modes. For a coarse plausibility check, we tested if the modelled migration rates (maximum level estimation) can explain the modelled postglacial range shifts (minimum level estimation). The postglacial range shifts were also modelled by means of SDM. The comparison of the modelled potential future range shifts and the migration rates was carried out in a direct comparison of the annual rates as well as in a spatial explicit comparison of the potential distributions assuming no migration, full migration and „realistic“ migration (based on the modelled migration rates. We calculated the percentage of the predicted future range that is reached assuming the modelled migration rates for different dispersal modes (range filling).

On the example of 140 European plant species, we predict future and postglacial range shifts by means of SDM. To address uncertainty due to climate change scenarios, we considered nine different environmental models. We decided to disregard uncertainty in predicted range shifts due to the used SDM algorithms in order to keep the analysis focused and manageable. Using a process-based approach, we simulate dispersal kernels (i.e. frequency distributions of dispersal distances) for the 140 plant species and several dispersal modes from which we derive estimations for the migration rates. We focus on dispersal by mammals (dispersal on animal's coat – epizoochory and dispersal by animals after feeding and digestion – endozoochory) and dispersal by wind (anemochory), which are both often regarded as highly relevant for LDD and because models for other dispersal types were not available. By combining migration modelling and SDM we aim to quantify the importance of dispersal limitation for the climate-change-induced range shifts in these 140 European plant species. A description of the general work flow of our study is given in fig. 1.

### Plant species

Our selection of plant species includes 140 species (table S1 in Supporting Information). Our aim was to consider as many plant species as possible. The limiting factor was the availability of data for the process-based modelling of the migration rates as well as occurrence data (at least 20 presences within the study area) for the SDM (see next sections).

The species set comprises species with different dispersal strategies: some of the selected species are clearly adapted to wind dispersal (e.g. *Salix hastata* L.), others to epizoochory (e.g. *Geum urbanum* L.). Some species are known to be dispersed frequently via endozoochory (e.g. *Chenopodium foliosum* Asch.), whereas others miss clear adaptions for LDD (e.g. *Papaver hybridum* L.); for details see [19].

### Species distribution modelling (SDM)

Based on the Atlas Florae Europaeae occurrence data (AFE, [20]), we modelled the potential range under the last glacial maximum (LGM); and for current and future climatic conditions for the 140 species, taking 19 bioclimatic variables into account (see table S2 and S3). Since we focused on climate change we only considered climatic variables. These variables can be regarded to be the most important for the expected large-scale climate-change-induced range shifts. The spatial resolution of our study is beyond the scale of e.g. edaphic factors. Taking factors such as edaphic factors into account would thus not impact the large scale patterns of the modelling results. All variable layers were rescaled to a spatial resolution of 0.5° by computing the mean. This spatial resolution is in accordance with the 50×50 km^2^ resolution of the AFE data. We used the presence-only modelling algorithms Maxent [21] version 3.3 with minor modifications of the default settings (only linear, quadratic and product features, maximum iterations = 50 000)). Maxent is relatively robust against collinear variables, i.e. collinearity does not affect the performance of Maxent, but can impair the interpretation of variable influence [22], [23]. As we did not focus on variable contribution, we decided to use all 19 bioclimatic variables. The study area ranges from 16° to 84° north and from 42° west to 84° east and covers the AFE area completely. In order to transform the continuous modelling results into binary presence-absence data, we used an optimized threshold that maximizes the percentage of correct predicted presences and absences (sensitivity = specificity; [24])

### Calculating potential range shifts

As a measure of the potential range shift due to climate change we considered, first the distances between the centroids of the predicted current and future ranges (weighted by the modelled continuous occurrence probabilities), and second the maximum of the distances of the range margins between the current and the future range (also weighted by the continuous occurrence probabilities). We defined the range margin in a certain direction as the 95^th^ percentile of the modelled occurrence probabilities (exceeding the sensitivity = specificity threshold) in the respective direction (see fig. S1 for an illustration of this method). We considered the range margin in 36 directions (every 10 degrees). To calculate the distance between the modelled range limits we took Earth's curvature into account (assuming an Earth's radius of 6 371 km). Since in Europe many plant species are expected to shift their potential ranges rather north-eastwards instead of strictly northwards we decided to consider range shifts in all directions.

### Postglacial range shifts

In order to test whether the simulated migration rates are in accordance with realized postglacial migration rates of the species, we used the SDM results for the potential ranges under LGM conditions and compared these with the results under current climatic conditions. As we assume that postglacial migration and resettlement mainly started 10 000 years back (cf. [25]) we calculated the average annual range shifts by dividing the absolute shift by 10 000 years. By dividing the absolute shift by 10 000 years, the calculations of average annual range shift rates are subjected to the assumption that species moved during the whole period of time. As it is likely that the ranges of at least some species have been stable within the past few thousand years, the calculated annual range shift rates are thus rough estimators for the minimum level of yearly migration rates that were realized.

### Future range shifts

In order to assess the uncertainty due to future development of the environment, we used a combination of three IPPC emission scenarios (A1, A2 and B2, [26]) and three global circulation models (GCMs: CCCMA, CSIRO and HADCM3) for 2080 (i.e. in total nine environmental models). To predict the annual future range shifts we divided the absolute range shifts (from current to 2080) by 105 years, as the data on current climatic conditions comprise the period of 1950 to 2000 (and 1975 is the midpoint between 1950 and 2000).

### Dispersal kernels

For each of the 140 plant species we simulated 100 000 dispersal distances (dispersal kernel) for dispersal on animal coats (epizoochory), for dispersal by animals after feeding and digestion (endozoochory) and for dispersal by wind (anemochory).

### Dispersal by animals

Dispersal distances for zoochory were computed based on information of animal movement and retention times in the digestive tract as well as retention times on the coat of animals. As animal movement and retention time are species-specific we considered nine ‘model’ animal species differing in body mass, day-range and home-range to generate the presented kernels (table S4). The study of these model animal species does not aim to study these species exactly but rather to give an overview to what extent the dispersal kernels can differ between animals of different body masses, day-ranges, and home-ranges.

The proportion of diaspores still attached to the animal's coat after a certain time was modelled by a bi-exponential function of the form
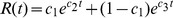
(1)The three coefficients of this bi-exponential function were empirically estimated from standardized lab-experiments on a coat-shaker (see [27] for a description of the lab-experiments and the coat shaker). For 103 plant species the proportion of diaspores still attached to cattle coat was determined after ten time periods (up to 24 hours) with up to five repetitions (see table S9 and fig. S2). As seeds that were still attached to the animal coat on the coat shaker after 24 hours are supposed to remain there for a long time (which is probably not the case under natural conditions), we standardized the measured values by subtracting the minimum value and dividing it by the range of the measured values. For each repetition, a bi-exponential function (formula 1) was fitted. Then the parameters were averaged over the repetitions for the final parameters of the bi-exponential function. With an R^2^ of 0.92 on average (table S5) the determined bi-exponential curves approximate the measured values quite well.

The proportions of diaspores that have then fallen out at a certain time are modelled as 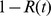. From these cumulated density functions (CDFs) that model the proportion of diaspores that had fallen out at a certain time we drew 100 000 retention times to generate the discrete dispersal kernels. This was only possible for eight of the 140 species considered in this study. For the other 132 species we had no experimentally measured retention times. For these 132 species we determined the two most similar species from the 103 species from the experiment in terms of diaspore morphology, mass, and retention potential (table S1) and sampled 100.000 retention times from the CDFs associated with the experimentally fitted bi-exponential functions of these two most similar species using the inverse distance in trait space as a weighting factor that determines sample size (table S5).

The proportion of diaspores still in the digestive system of animals after a certain time was modelled as a logistic function of the form
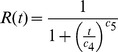
(2)We approximate the 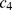 coefficient by the mean retention time (MRT) in the digestive system of the animal. MRT was parameterized for the nine model animal species based on a literature research (table S4). The 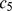 coefficient regulates the ‘tail’ of the curve, i.e. 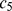 determines the modelled proportion of diaspores that stay over a longer time in the digestive tract. The 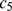 coefficient was set to 3.5. Such logistic functions (with 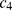 = MRT and 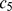 = 3.5) match empirically measured excretion data that were derived for sheep and cattle [28] very well (see fig. S3, average R^2^ = 0.99, N = 5) and are also reasonable for the nine model animal species (see fig. S4).

Again the CDF 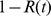 that gives the proportion of diaspores that have been excreted at a certain time is used to draw 100 000 retention times at random.

We modelled animal movement as a correlated random walk (CRW) with a range of three angles characterizing different movement patterns [18]. Average movement speed (calculated from day-ranges, i.e. the daily travelled distances) and size of the home-range were parameterized based on animal species-specific data, which were compiled from literature (table S4). The CRW modelling yields a probability distribution of net distances for seed dispersal after a certain retention time with a temporal resolution of 1 min (fig. S5).

To model seed dispersal distances, we first draw a retention time randomly based on the CDFs associated with the functions in formula 1 and 2 to determine the time that a randomly selected seed remains on an animal coat or in the digestive tract, respectively. We then draw a distance, according to the probability distribution derived from the CRW at the sampled retention time to determine the distance that a randomly chosen animal individual covers while the seed remains in the coat or digestive tract. These two steps were repeated 100 000 times in order to generate the discrete dispersal kernels, i.e. the frequency distributions of dispersal distances.

### Anemochory

Wind dispersal was simulated with PAPPUS, a mechanistic wind dispersal model that simulates flight trajectories of individual seeds. The model and its validation are described in detail in [29]. The model uses initial release height and the falling velocity (see table S1) of diaspores as input parameters and empirical measurements of the wind field (including turbulence) for three different habitats (field, forest and grassland) over three years. Our study therefore includes variability in migration rates due to differences in meteorological conditions between habitats and years. For each habitat and year we modelled 100 000 dispersal distances.

### Estimation of the potential migration rates

Based on the discrete dispersal kernels, we estimated the potential migration rate according to [30] as the expected value of the maximum of a random sample of the size of the net reproductive rate R_0_, divided by the generation time T. R_0_ is here defined as the number of offspring expected from an individual at the time of seed release [30]. We set R_0_ to 10 000 for all species in order to get an estimate of maximal migration rates under optimal conditions, specifically populations with high fecundity in a homogeneous, not fragmented landscape. The generation time T was approximated by the mean age of the first flowering of species with the same life form that was derived from the CLOPLA data base ([31], table S1). In order to calculate these values, all species in the CLOPLA data base were grouped according to their life form. For each group the average age of first flowering was calculated. In cases in which a range was given for the age of first flowering of a certain species we only considered the lowest number to calculate the group average. For a single species we thus considered the quickest time to sexual reproduction as such extremes are especially important for determining migration rates.

Data analysis was carried out with R 2.15.0 (R Foundation for Statistical Computing, 2012). Maps were generated with ARCGIS 10 (ESRI, Redlands, USA).

## Results

### Predicted future range shift

**Figure 2:**
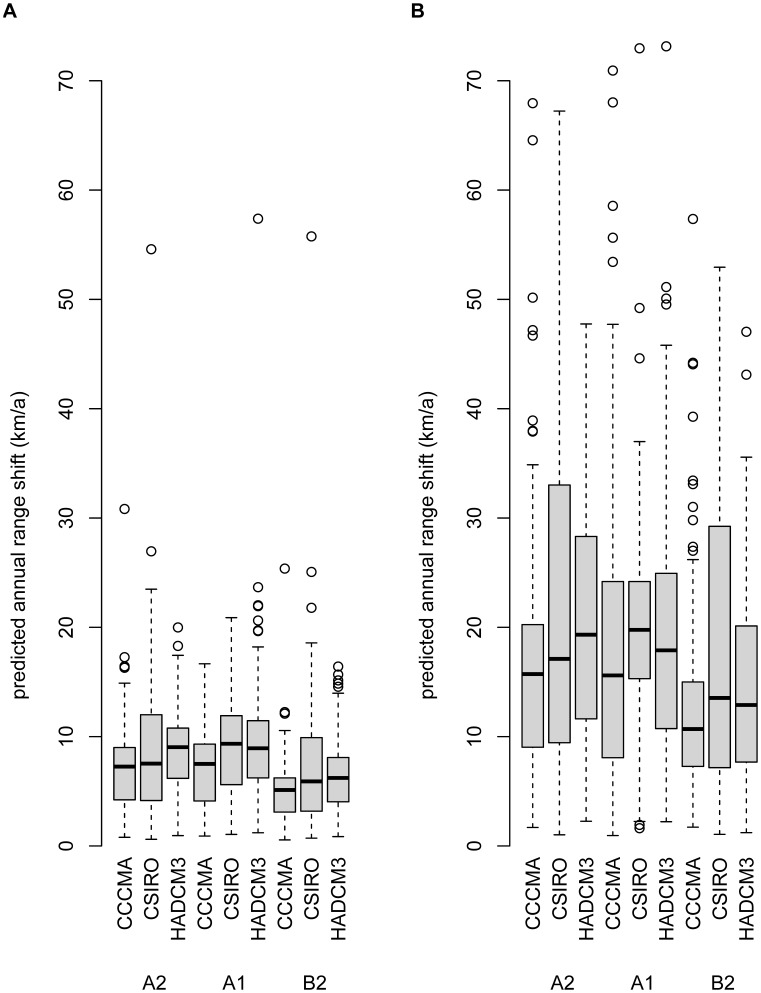
Predicted future range shifts (annual averages) according to the nine environmental models for 2080. Predicted shifts of the centroids (A) and of the range margins (B). Each boxplot represents N = 140 plant species.

According to our results, species will have to migrate rapidly in order to follow the predicted climate-change-induced range shifts. The predicted range shifts for the centroids are 7.8 km/a on average, and range up to several 10 kilometres for few species (fig. 2A). Considering the shifts of the range, margins result in significant higher values: 17.9 km/a on average and several 10 kilometres for some species (fig. 2B, table S6). The predicted range shifts for both methods differ significantly between the IPCC scenarios, as well as between the GCMs (table S7).

### Migration rates

**Figure 3:**
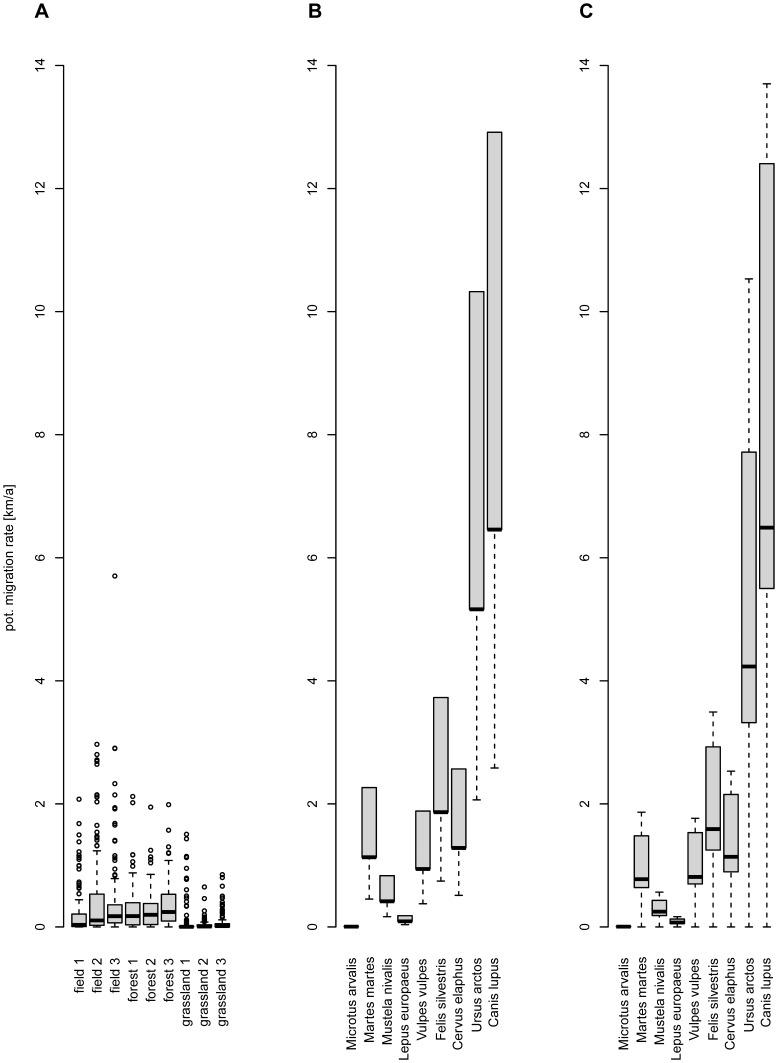
Predicted migration rates for A) dispersal by wind (anemochory) for nine different meteorological scenarios B) dispersal by animals (endozoochory) for nine different animal species C) dispersal by animals (epizoochory) for nine different animal species. Each box represents N = 140 plant species.

The average modelled migration rate over all 27 dispersal modes and plant species is 1.6 km/a, but the variation is considerable (fig. 3). Larger animals (e.g. *Canis lupus*, *Ursus arctos*, *Cervus elaphus*, *Felis sylvestris*) allow higher migration rates compared to dispersal by smaller animals and dispersal by wind. Migration rates are closely related to day- and home-range of the animals: Spearman correlation coefficient r_s_ between day-range and average migration rate are r_s_ = 0.77 (endozoochory) and r_s_ = 0.71 (epizoochory) and between home-range and average migration rate: r_s_ = 0.68 (endozoochory) and r_s_ = 0.65 (epizoochory). Remarkably, the distributions of migration rates considering endozoochory and epizoochory are quite similar (fig. 3 B,C).

### Modelled migration rates vs. postglacial migration

The derived annual range shifts during the post glacial for the 140 species is on average 0.12 km/a and varies between 0.01 and 0.44 km/a considering the centroids. Regarding the range margins, it is 0.18 km/a on average and ranges from 0 to 0.87 km/a (fig. S7).

As these range shifts have been realized during the postglacial they can be seen as a coarse estimator for migration rates that can be realized by the species at least. Process-based seed dispersal models should thus result in migration rates that are above these minimum migration rates.

For nine plant species we could not estimate the potential range shift since the LGM as these species were predicted not to occur within the study area in the LGM. For all other species, the migration rates modelled with the process-based seed dispersal models used in this study are higher than modelled annual postglacial range shifts (for both methods centroid and margin) when considering dispersal by large animals (i.e. for at least one of the 18 dispersal modes). Considering dispersal by wind, the process-based modelled migration rate is higher than the modelled past range shift for about 75% of the considered plant species (103 of the 131 species; for both methods centroid and margin).

### Predicted future range shifts vs. modelled migration rates

**Figure 4:**
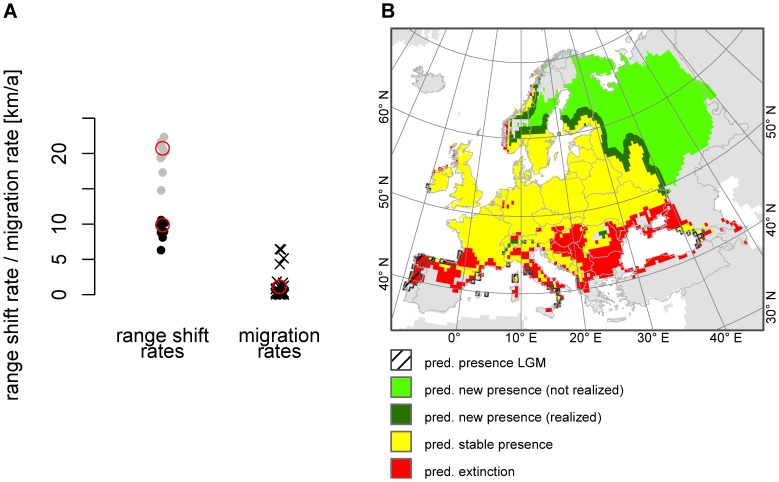
Potential dispersal limitation on the example of *Geum urbanum.* A) Comparison of the potential future range shift rates according to the nine environmental models and the process-based modelled migration rates according to the 27 dispersal modes for *Geum urbanum*. The potential future range shift rates can be considered an estimator for the migration rates required in order to fulfil the potential future range completely. They are displayed as dots (black: centroid method and grey: margins method). The process-based modelled migration rates are displayed as black crosses. The values for the dispersal mode and the environmental model used in the map in fig. 4B (epizoochorous dispersal by *Cervus elaphus* and the A1 CCCMA environmental model for 2080) are marked by red circles. B) Potential range shift and dispersal limitation on the example of *Geum urbanum*. The map is based on a realized migration rate of 1.12 km/a corresponding to epizoochorous dispersal by *Cervus elaphus*. The predicted future range is according to the A1 CCCMA environmental model for 2080. Projection: Europe Albers Equal Area Conic.

The modelled migration rates exceed the modelled future range shift rates (that can be considered an estimation for the required migration rates in order to fulfil the potential future range completely) in about only 8% (centroid method) and 3% (margin method) respectively of the 243 cases (number of cases resulting from the combination of nine environmental models and 27 dispersal modes, see also fig. S8). An example for the mismatch between the modelled potential migration rates (according to the 27 dispersal modes) and the potential range shift rates (according to the nine environmental models) is shown for *Geum urbanum* L. in fig. 4A. Only the modelled migration rates for *Canis lupus* (endo- and epizoochorous) exceed the two lowest predicted range shift rates (B2 CCCMA and B2 CSIRO for the centroid method), while in all other cases modelled migration rates are considerably lower than predicted future range shift rates.

**Figure 5:**
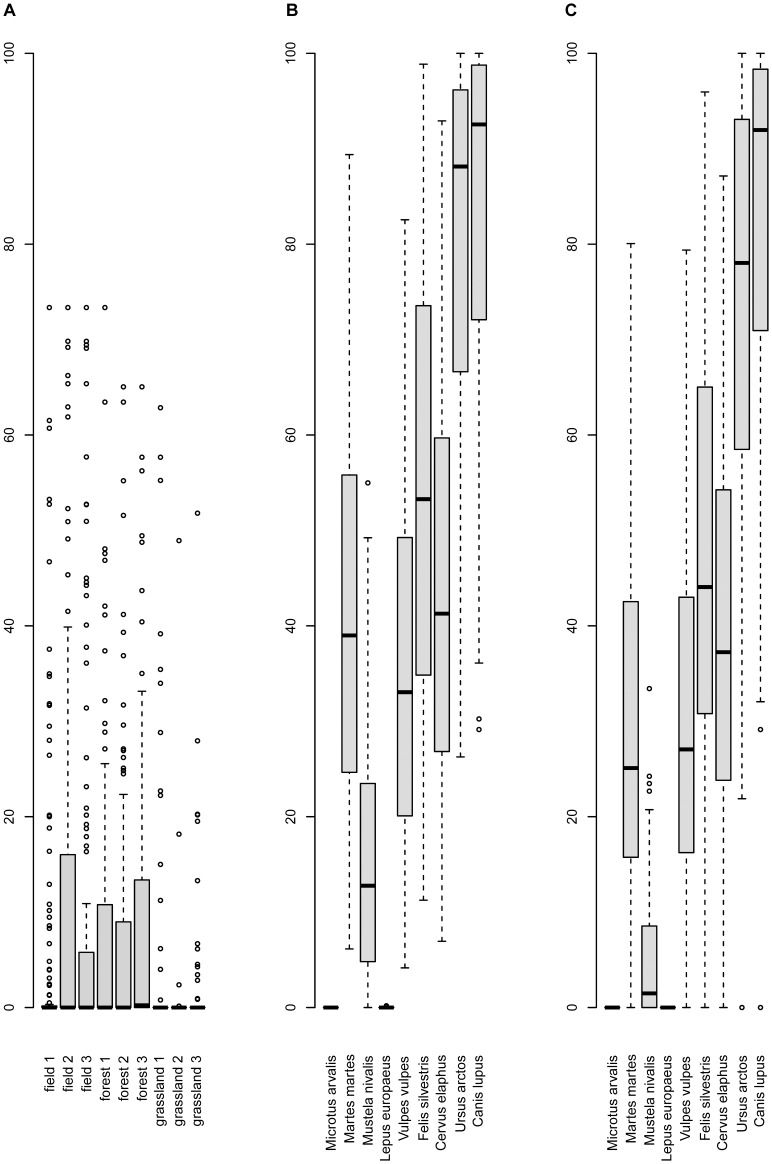
Percentage of the predicted future range that is reached assuming dispersal by wind (A), endozoochory (B) and epizoochory (C) respectively. The potential future range was estimated according to the A1 CCCMA environmental model for 2080. Each boxplot represents N = 140 plant species (see also fig. S6 and table S8).

Only a few species are predicted to be able to fulfil the potential future range completely and this is only the case for dispersal by large animals (fig. 5, fig. S6 and table S8). For dispersal by the animals with the largest day- and home-ranges (*Canis lupus* and *Ursus arctos*), 56 to 76% of the 140 species are predicted to fulfil their future ranges up to 90% on the example of the A1 CCCMA environmental model. For *Cervus elaphus*, an example of a more abundant large herbivore, 16% (for endozoochory) and 12% (for epizoochory) of the species are predicted to fulfil their future ranges up to 90% according to the A1 CCCMA environmental model. Considering anemochory and dispersal by animals with small day- and home-ranges only few species are predicted to fulfil their future ranges up to 90% (table S8).

**Figure 6:**
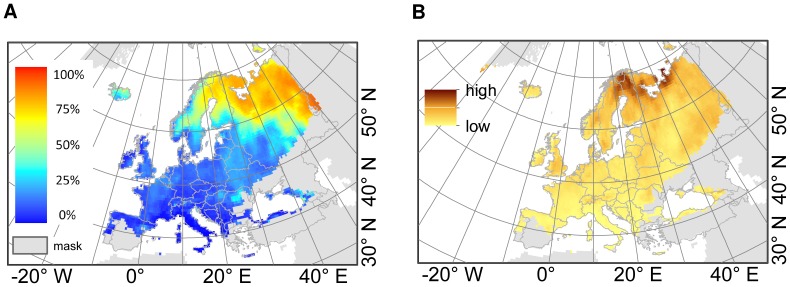
Biodiversity loss due to dispersal limitation in terms of the considered 140 plant species. A) Difference between predicted future distributions (2080) assuming full dispersal and “realistic” dispersal (according to our modelled migration rates taking 27 dispersal modes for migration into account): The differences were calculated for each of the nine environmental models and then averaged. In grey: areas where very few of the 140 species are predicted to occur in 2080 (<10% of the 140 species). B) Uncertainty of the model predictions: Standard deviation of the difference between full dispersal and “realistic” dispersal over the results for the nine environmental models. Projection: Europe Albers Equal Area Conic.

Most of the 140 plant species considered in this study shift their potential range north-eastwards (fig. 6, fig. S9 a,d). Taking our results for the migration rates for the 27 dispersal modes into account; large parts of the potential new range will not be reached. This results in a loss of potential biodiversity in the considered species of up to 100% in the north-east of the study area, compared to the potential (new) biodiversity assuming full dispersal (fig. 6).

## Discussion

During the postglacial period, plant species responded successfully to climate warming by adaptation to new environmental conditions, by migration in order to follow suitable conditions, or both [15]. Future climate change is predicted to bring faster changes than during the postglacial period. Thus migration and dispersal limitation may become a key driver for survival or establishment of species under climate change [32], [15], [4] and should therefore indispensably be considered when estimating plant species' response to climate change. Our process-based modelled migration rates overcome the simplification assuming full or no dispersal in order to provide more realistic and species-specific predictions. Such complex models are among the best approaches to estimate climate-change-induced range shifts that take dispersal limitation into account (cf. [14]). But even complex models inherently involve uncertainty [14], [6]. Taking three IPCC scenarios and three GCMs into account, we were able to analyse uncertainty in required range shifts due to different future developments of the environment. The effect of different dispersal modes and vectors on potential migration rates was addressed in process-based modelling. This issue can - from a slightly different perspective - also be regarded as uncertainty, as we do not know how important the different dispersal modes and vectors are for a certain plant species. On the other hand, it was beyond the scope of this study to address the uncertainty in prediction due to different SDM algorithms. Therefore, we applied only one algorithm, Maxent, that is one of the most commonly [33] and successfully (e.g. [34]) applied algorithms for SDM. We assume that even if the spatial pattern of the SDM results for a certain species may differ subjected to the used algorithm, the predicted range shifts are of the same magnitude [35]. The fact that our results for the future range shifts are in the same order of magnitude as those presented by [36] (fuzzy climatic envelopes) and [37] (climate envelopes) gives a further hint for the robustness of this assumption.

### How fast will plant species ranges shift in the future?

[36] calculated an average annual future range shift for 26 forest herbs between 5.6 km/a (B1 scenario) and 9.3 km/a (A2 scenario) and for 130 North American tree species [37] found annual rates for the predicted northwards shift of 0.06°, i.e. 7.1 km (averages taken over two scenarios and three GCMs). In both studies, range shifts are calculated based on range centroids and are comparable to our results of about 7.8 km/a (average over three scenarios, three GCMs and 140 plant species). Considering the range margins, the predicted range shift averages 18.0 km/a. The future range shifts differed between the environmental models, but the differences were relatively small. In contrast, we found much greater variations within the predicted migration rates due to different dispersal modes (see fig. 2 and 3).

Range shifts are usually calculated based on the centroids (cf. [36], [37], [4]). Our results show that considering the shift of the centroids may underestimate the distances that species have to overcome in order to fulfil the new ranges completely in case of range expansion. Therefore we suggest considering the range margins for this application.

### Does process-based modelling provide realistic estimations of plant migration?

For the postglacial resettlement, we found a modelled range shift that has been realized by migration of up to 440 m/a (centroids) and 870 m/a (range margins) respectively. Based on pollen-based reconstructions postglacial range shifts are denoted for tree species to range from 100 to 1000 m/a [38].

Assuming the migration rates computed with our process-based models, all considered plant species would have been able to track the postglacial range shifts. We thus overcome Reid's paradox, i.e. the observation that dispersal abilities of most herbs and trees are too limited to explain their resettlement of northern latitudes following glacial recession [39]. However, our estimation of the migration rates is based on high R_0_-value resulting in optimistic migration rates. In addition, the modelled postglacial range shift rates have to be considered a minimum-level-estimator as we assumed that the species have moved the whole time during the last 10,000 years. In contrast the modelled postglacial range shift rates (taken as an estimator for the realized postglacial migration rates) may be overestimated as small refugia may have been disregarded due to the relatively coarse spatial resolution of the environmental data in SDM.

Over the past 50 years, a northward range shift of terrestrial plants in the Northern Hemisphere of about 610 m/a was observed that is closely related to climate change [40]. This observed range shift can also easily be explained with the migration rates derived from our process-based models.

However, there are still uncertainties related to process-based modelling of migration rates and many possibilities for improvements. CRWs are surely a major simplification and radio-tracking data would clearly provide a better approximation of animal movement, especially in case of spatial explicit studies and in order to make spatial explicit predictions. But as we are mainly interested in the net distances for animal-types differing in day-ranges and home-ranges, CRWs appear to be a sufficient approach. Our implementation provides a feasible way to model animal movements for several animal species based on a few parameters and explicitly considers animal specific day- and home-ranges.

The fact that we assume the same net reproduction rate R_0_ for all species and all dispersal modes is a simplification due to the lack of better data and the high dependency of fecundity on the local environmental conditions. Using a high value for R_0_ yields higher estimations for migration rates (the relation between R_0_ and the estimated migration rate is shown in fig. S10). In order to assess whether plant species will be able to keep pace with a rapidly changing climate, we decided to consider a maximum-level estimation for the potential migration rates and a minimum-level estimation for the potential future range shift rates (that can be considered an estimation for the required migration rates in order to fulfil the potential future range completely). In addition we get optimistic estimates for the migration rates as we assume dispersal under optimal conditions in a homogeneous, not fragmented landscape. According to these settings, it seems likely that our study even underestimates the effect of dispersal limitation on future range filling.

### Are plant species able to keep pace with a rapidly changing climate?

According to our results many European plant species will hardly be able to keep pace with rapidly changing climate by means of wind or animal dispersal. The predicted annual range shift rates exceed the modelled migration rates in many cases. Hence, it must be assumed that many species will be dispersal limited and therefore will not be able to fulfil their potential future range completely. As displayed on the example of *Geum urbanum* in fig. 4B we distinguished a future new range that is possibly reached by 2080 assuming dispersal by a certain mode (here a frequent large herbivore like *Cervus elaphus*, epizoochor) and a future new range that may not be reached. Note that this is a somewhat simplified perspective based on the modelled potential distribution. Intermediate stages and refuges as well as the fact that it is likely that colonization will be slower on the leading edge than extinction on the trailing edge are not taken into account as this is beyond the means of SDM.

This mismatch between predicted range shift rates and modelled migration rates can have a great impact on ecosystem properties and processes [9] and thus on biodiversity. Most of the species are predicted to be dispersal limited as they are not able to fulfil their future ranges, except via dispersal by large animal species with large day- and home-ranges which rarely occur in many parts of Europe.

Of course there is no need for dispersal to match, year-on-year, changes in climate suitability as it might not matter whether a species reaches its new range as soon as it becomes suitable or a few years later. But the example of *Geum urbanum* (fig. 4) shows that it is not a question of a few years until a species is predicted to reach the northerly range margin, but of decades or even centuries which may result in profound consequences for biodiversity and ecosystem functioning.

The implementation of plant dispersal limitations into projections of future species' distributions clearly yields more realistic estimations than assuming full or no migration. Our results incorporating species-specific migration rates are apparently closer to the results assuming no-migration than to the results assuming full migration (fig. S9), but differ considerably between plant species.

However, the migration capacity of a species is determined not only by its dispersal characteristics but also by the structure of the landscape the species live in [13]. In our approach, we did not take natural dispersal barriers or human-driven habitat fragmentation into account. Because of the high degree of fragmentation of most European landscapes, animals might move over short distances only, sites that are suitable for colonisation might be rare and distinct, and population sizes may be small. These facts may slow down realized migration [14] and our results may therefore be too optimistic.

On the other hand, we decided to consider only “natural” dispersal vectors and did not take dispersal by humans into account, which is also an important vector especially for long distances in cultural landscapes [41], [42], [5]. A challenging task in future studies will be the incorporation of human-mediated dispersal. Human-mediated dispersal is still difficult to measure [5] and we lack data and models in order to simulate migration rates considering human-mediated dispersal. It seems realistic to assume that human-mediated dispersal can considerably diminish dispersal limitation, in particular for species from man-made and disturbed habitats or if seeds are intentionally dispersed.

Birds are another important dispersal vector that we did not take into account. The reason for this is that we did not have parameters of avian movement to model migration rates. Furthermore the plant species considered in this study, are not particularly adapted to dispersal by birds, so that we assume that dispersal by birds is almost not relevant for these species. For future studies considering species that are particularly adapted to dispersal by birds, e.g. fleshy fruit species, it would be desirable to make an effort to take dispersal by birds into account.

Regarding our study we doubt whether dispersal by birds will lead to fundamentally higher migration rates compared to dispersal by the nine model mammals that we considered in this study. According to [43] mammals roam over larger distances compared to seed-dispersing birds, have longer gut passage times and are thus able to provide longer dispersal distances than birds.

Animals (birds or mammals) that overcome large distances in a cyclic annual journey may also be considered an LDD vector for seed dispersal. But it is debatable whether these cyclic annual journeys act as a vector for a northward migration, as diaspores of most plant species ripen in late summer or autumn, when these possible vector species move from north to south.

### Which dispersal modes are most effective in terms of high migration rates?

Our results match the assumption that LDD is typically driven by large mammals and birds of passage [41], [18]. Specifically, we found the highest modelled migration rates for dispersal by large carnivores like *Canis lupus* and omnivores like *Ursus arctos*. As isolated LDD events are crucial for migration, the rareness of a vector animal species does not necessarily change the fact that these species are potentially very effective vectors. But we have to keep in mind that *Canis lupus* and *Ursus arctos* are extremely rare and do not occur at all in parts of Europe. The rareness of these species surely decreases or even precludes their relevance as dispersal vectors for most plant species and in most habitats. Dispersal by large herbivores (e.g. *Cervus elaphus*) was somewhat less effective in terms of computed migration rates, but they represent the only frequent animals with large day- and home-ranges left in many parts of the European man-made landscape dispersing seeds via epi- and endozoochory. We therefore argue that these frequently occurring large herbivores are most important for long distance dispersal of plant species dispersed by mammals.

Dispersal by wind generally yields considerably lower migration rates than dispersal by animals. However, wind is almost universally available and the importance of wind may thus be underestimated in our study, which is based on identical vector densities and net reproduction rates R_0_ per dispersal mode. Net reproduction rates are affected by many other factors: e.g. the probability of a seed to be transported by a certain dispersal mode, survival rate after digestion, the probability for seed germination and seedling establishment. The fact that the results for endo- and epi-zoochory are quite similar may also be partly assigned to the unmodified net reproduction rate, which must be attributed to the lack of suitable data.

### Conservation aspects

Climate change is already affecting the distribution patterns of plant species and clearly poses a severe threat to biodiversity [10]. Many species are predicted to have considerably smaller ranges due to climate change [1] and species with small ranges are particularly endangered [1]. Species that are predicted to expand or shift the range of suitable habitat conditions may not be able to fulfil their potential new ranges due to dispersal limitation. Hence, dispersal limitation leads to serious losses in (potential) biodiversity.

As large animal species are expected to be very effective vectors for seed dispersal, nature conservation means should be taken into consideration to promote the occurrence of large mammals in Europe (e.g. reintroduction of European bison, wolf). A reduction of the landscape fragmentation should also be a primary objective to work against dispersal limitation. A suitable means to promote LDD is to make dispersal corridors available, as biodiversity is thought to be higher in interconnected biotopes [44], [45]. Due to the difficulties in estimating and predicting the effects of the intentional introduction of species, this is a controversial means to counteract dispersal limitation.

## Supporting Information

Figure S1 **Calculation of the shift of the range margins: a) predicted range of** ***Geum urbanum*** **under current climatic conditions, in black margins in 10° steps b) predicted range of** ***Geum urbanum*** **under future climatic conditions according to the A1 IPCC scenario GCM CCCMA for 2080, in black range margins in 10° steps c) predicted range margins for** ***Geum urbanum*** **under current climatic conditions (orange) and under future conditions (light blue).** As the range margin in a certain direction, we defined the 95th percentile of the modelled occurrence probabilities (exceeding the sensitivity = specificity threshold) in the respective direction. The distance between the current range margin and the future range margin is largest in direction north east. This distance is taken as a measure for the maximal range shift.
(TIFF)

Figure S2 **The proportion of seeds still attached to the animals coat after a certain time was modelled as a bi-exponential function (see formula 1 in the main document) and empirically fitted by means of standardized lab-experiments on a coat-shaker (see** [**27**] **for a description of the lab-experiments and the coat shaker).** We standardized the measured values by subtracting the minimum value and dividing by the range of the measured values. For each repetition (displayed as red, blue, green, orange and yellow dots) the c1, c2 and c3 parameters (see formula 1 in the main document) were fitted separately and then averaged (black curve). The displayed R^2^ is the average over the four respectively five R^2^ of each repetition.
(TIFF)

Figure S3 **The proportion of seeds still in the digestive tract after a certain time.** Measured values (mean of the proportion of seeds still in the digestive tract of 20 plant species) for a) sheep and b) cattle (displayed as dots, for each species five repetitions) were taken from [28]. A logistic function (see formula 2 in the main document) was fitted to the measured values with the mean retention time as c4 parameter and c5 = 3.5. As mean retention time we took for each species the average mean retention time of 12 plant species (taken from [28]). The R^2^ for the fitting is 0.96±0.03 for sheep and 0.98±0.02 for cattle (N = 5 repetitions).
(TIFF)

Figure S4 **The proportion of seeds still in the digestive tract after a certain time for the nine model animal species.** The mean retention time (MRT) is displayed as dotted line.
(TIFF)

Figure S5 **Probability distributions of the dispersal distances modelled by the correlated random walks (CRWs) for the three angles (90°, 45° and 22.5°) on the example of three different animal species with different movement patterns.** Colours represent the probability that the animal species has covered the respective (net) distance within the respective time.
(TIFF)

Figure S6 **Percentage of the predicted future potential new range (on the example of the A1 CCCMA environmental model for 2080) that is reached assuming dispersal by wind and animals.** Each boxplot represents 140 plant species (cf. fig. 5 in the main document).
(TIFF)

Figure S7 **Modelled annual range shifts of the N = 140 European plant species since the Last Glacial Maximum (LGM) based on the distance between the centroids respectively on the range margins of the modelled past and current range.** The period in which the species migrated to fulfil their current ranges was set to 10 000 years.
(TIFF)

Figure S8 **Proportion of the modelled migration rates that exceed the modelled annual range shifts based on the nine climatic models.** Each boxplot represents for each of the N = 140 plant species 243 proportions ( = 27 dispersal modes * nine environmental models).
(TIFF)

Figure S9 **Distribution of biodiversity considering 140 species a) under current climatic conditions b) under future climatic conditions according to the A1 Scenario CCCMA for 2080 assuming no migration c) under future climatic conditions according to the A1 Scenario CCCMA for 2080 assuming “realistic” migration d) under future climatic conditions according to the A1 Scenario CCCMA for 2080 assuming full migration.** For the no-migration map we only considered the overlaps between the current and the future ranges, for the realistic migration rate maps we took additionally the overlaps between a buffer of the estimated annual migration rates multiplied with 105 years around the current range and the future range into account. For the full migration map we considered the entire future ranges. a)b)d):100% means that all 140 species are predicted to occur at this place. c): 100% means that all 140 species are predicted to occur at this place and in terms of potentially new areas: the place is predicted to be reached by all 27 dispersal modes. Projection: Europe Albers Equal Area Conic.
(TIFF)

Figure S10 **Sensitivity of the estimation of the migration rate to the net reproduction rate R_0_ on the example of** ***Geum urbanum*** **, dispersal by** ***Cervus elaphus*** **(epizoochorous).** R_0_ used in this study is marked by the dotted line, resulting in a migration rate of 1.12 km/a.
(TIFF)

Table S1 **Compilation of parameters and traits for the 140 plant species considered in this study: Hrel (releasing height) and Vterm (terminal velocity) are parameters used to model the Anemochory kernels (cf. Tackenberg 2003).** Data are taken form the D3-database (www.seed-dispersal.info). Retention potential (rtp.straight.hair), diaspore mass (dia.mass), morphology (dia.morph) and were taken from the D3-database (www.seed-dispersal.info). The age of first flowering (Age of FF) is estimated based on species life forms according to the CloPla data base (Klimešová & de Bello 2009). For eight of the 140 species considered in this study the parameters of the bi-exponential function (formula 1 in the main document) were experimentally determined (these species are marked with *). For the other 132 species the two most similar species (concerning diaspore mass, morphology and retention potential – cf. table S5) with experimentally determined parameters are given in this table (straighthair.spec1 and straighthair.spec2). f1 and f2 are the proportions of the retention times that are sampled from the bi-exponential distributions of the CDF for the prior species for that the CDF was empirically fitted (see table S5).
(DOC)

Table S2 **IPCC scenarios (IPPC third Assessment Report data) and GCMs used for species distribution modelling (SDM).**
(DOC)

Table S3 **List of the 19 environmental variables used for SDM (Hijmans** ***et al.*** **2005).**
(DOC)

Table S4 **Dispersal-relevant animal traits for the nine model mammals.** Given are trophic group, mean retention time of food in the gut (MRT), home-range size, day-ranges (i.e. daily distance travelled) and population density. Abbreviated references (in square brackets) are resolved immediately following this table and refer to MRT (1st number in brackets), home-range size (2nd), day-range size (3rd) and population density (4th). (Data mainly taken from Will 2008).
(DOC)

Table S5 **Parameters and traits for 64 of the 103 plant species for those the bi-exponential function was empirically fitted.** c1, c2 and c3 are the empirically determined parameters of the cumulative density function for epizoochory (formula 1 in the main document, with [t] = 1 min). The c1, c2 and c3 data represent means over several repetitions, all R^2^ with p <0.05. Only the species used for the mixture in this study (see table S1 are listed here. See table S9 for the raw values.
(DOC)

Table S6 **p values for the Wilcoxon tests between the future range shifts of the centroids and the future range shifts of the range margins.** For all environmental models the range shifts of the range margins are significantly higher than the range shifts of the centroids.
(DOC)

Table S7 **p values for the Kruskal-Wallis Rank Sum Test between the future range shifts referring to different environmental models (cf.** **fig. 1** **).**
(DOC)

Table S8 **Percentage of species that are predicted to be able to fulfil their future range up to 90% for the respective dispersal mode.**
(DOC)

Table S9 **Proportions still attached in cattle coat (prop_attach, measured values) after a certain time [min] for up to five repetitions (rep.) for the 64 species in table S10.** We standardized the measured values by subtracting the minimum value and dividing by the range of the measured values.
(TXT)
