# Correction: Are Plant Species Able to Keep Pace with the Rapidly Changing Climate?

**DOI:** 10.1371/journal.pone.0099248

**Published:** 2014-05-27

**Authors:** The PLOS ONE Staff

There are errors in the raw data presented in Table S9. The authors have provided a corrected version and the analysis presented in the published article is based on this corrected data.

## Supporting Information

Table S9 **Proportions still attached in cattle coat (prop_attach, measured values) after a certain time [min] for up to five repetitions (rep.) for the 64 species in table S10.** We standardized the measured values by subtracting the minimum value and dividing by the range of the measured values.
(DOCX)
