# Supplementary figures and images for: Are Plant Species Able to Keep Pace with the Rapidly Changing Climate?
Source: PLoS One. 2013 Jul 24;8(7):e67909. doi: 10.1371/journal.pone.0067909 (PMC3722234; doi:10.1371/journal.pone.0067909)

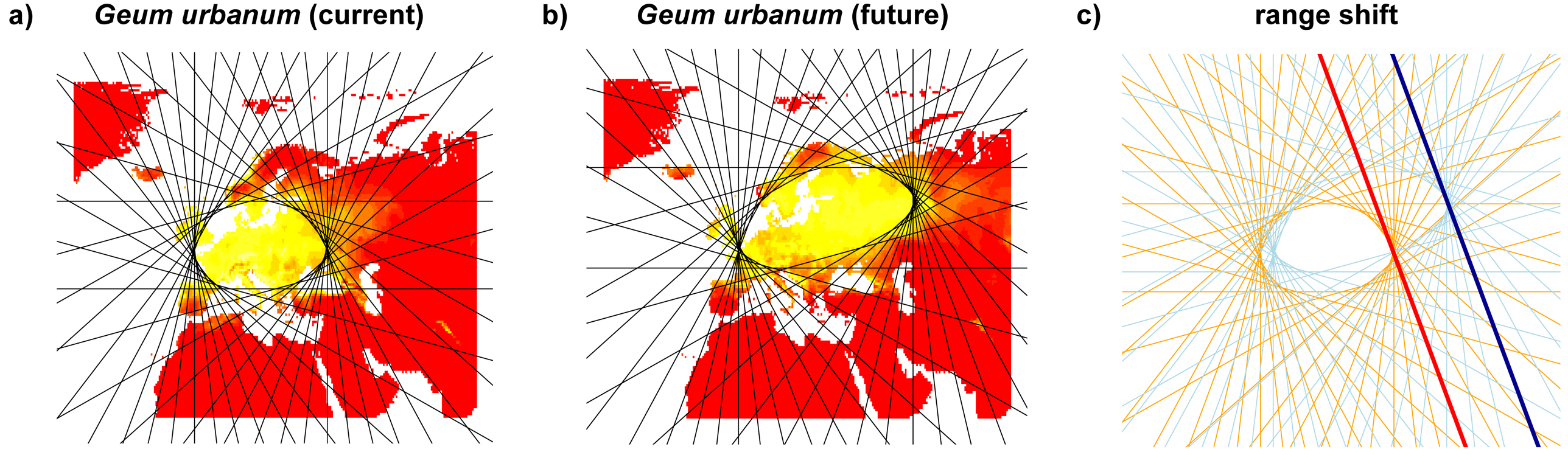

Supplement: Figure S1 — Calculation of the shift of the range margins: a) predicted range of Geum urbanum under current climatic conditions, in black margins in 10° steps b) predicted range of Geum urbanum under future climatic conditions according to the A1 IPCC scenario GCM CCCMA for 2080, in black range margins in 10° steps c) predicted range margins for Geum urbanum under current climatic conditions (orange) and under future conditions (light blue). As the range margin in a certain direction, we defined the 95th percentile of the modelled occurrence probabilities (exceeding the sensitivity = specificity threshold) in the respective direction. The distance between the current range margin and the future range margin is largest in direction north east. This distance is taken as a measure for the maximal range shift. (TIFF) [file pone.0067909.s001.tiff]

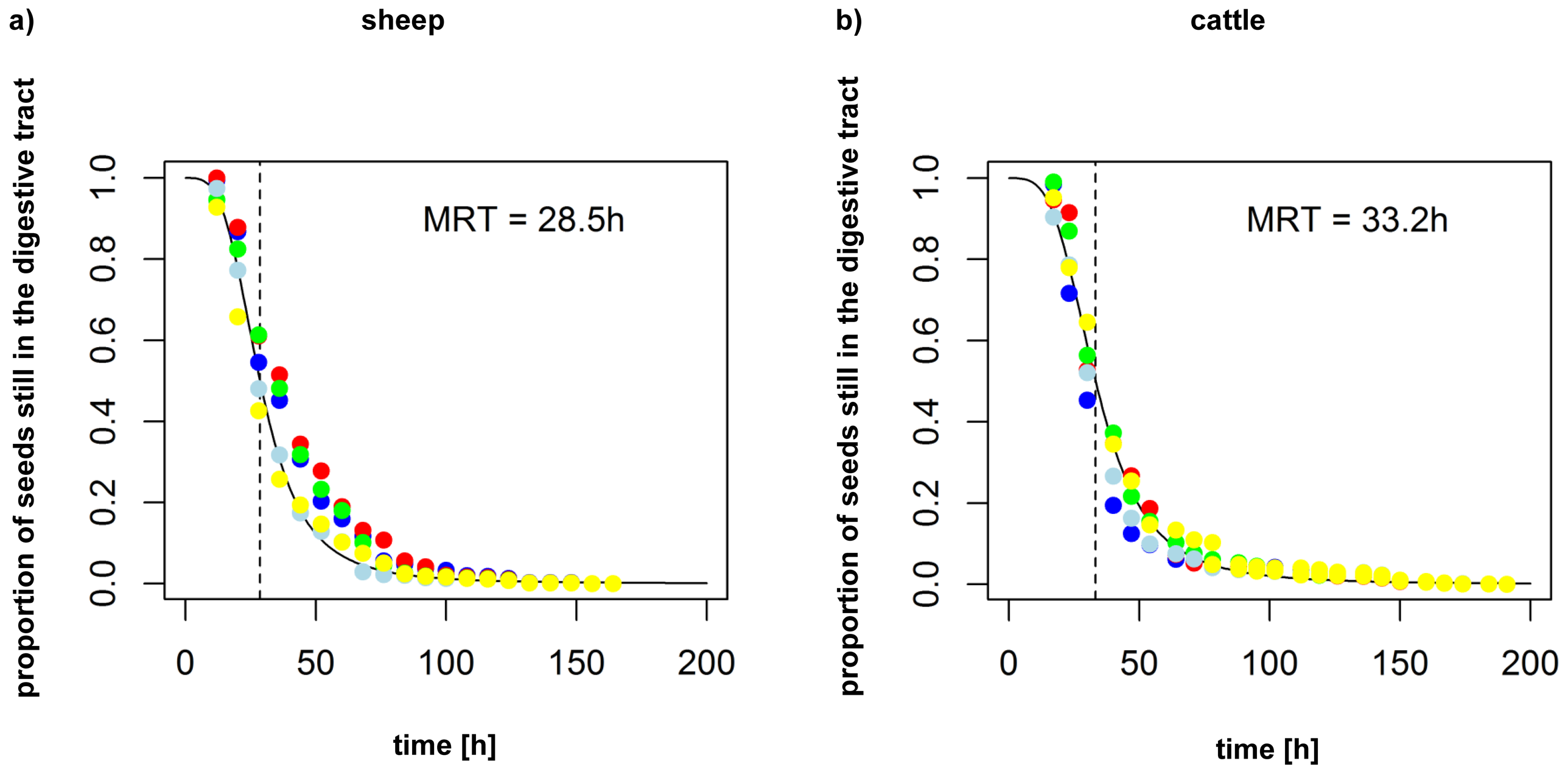

Supplement: Figure S3 — The proportion of seeds still in the digestive tract after a certain time. Measured values (mean of the proportion of seeds still in the digestive tract of 20 plant species) for a) sheep and b) cattle (displayed as dots, for each species five repetitions) were taken from [28]. A logistic function (see formula 2 in the main document) was fitted to the measured values with the mean retention time as c4 parameter and c5 = 3.5. As mean retention time we took for each species the average mean retention time of 12 plant species (taken from [28]). The R2 for the fitting is 0.96±0.03 for sheep and 0.98±0.02 for cattle (N = 5 repetitions). (TIFF) [file pone.0067909.s003.tiff]

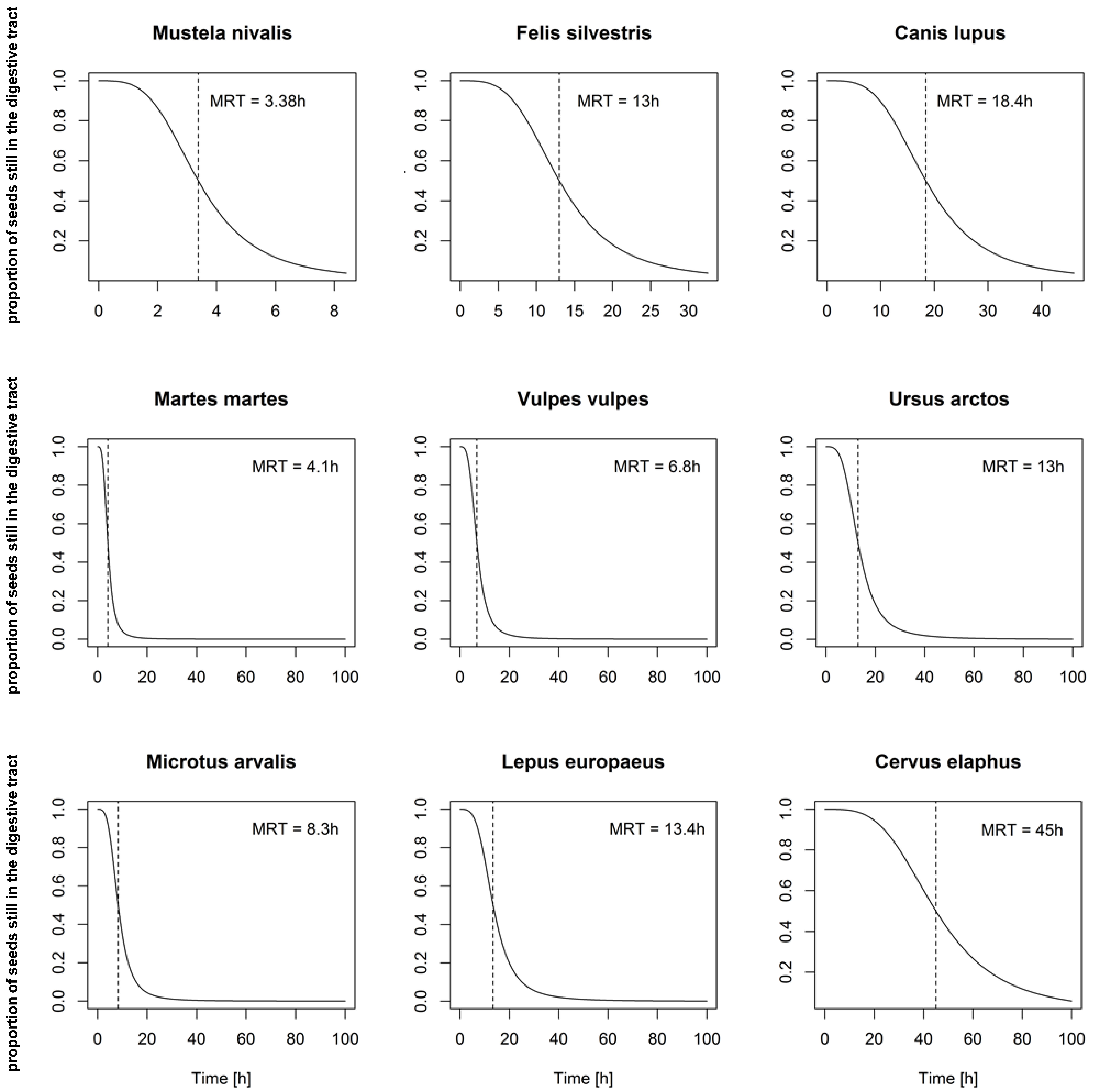

Supplement: Figure S4 — The proportion of seeds still in the digestive tract after a certain time for the nine model animal species. The mean retention time (MRT) is displayed as dotted line. (TIFF) [file pone.0067909.s004.tiff]

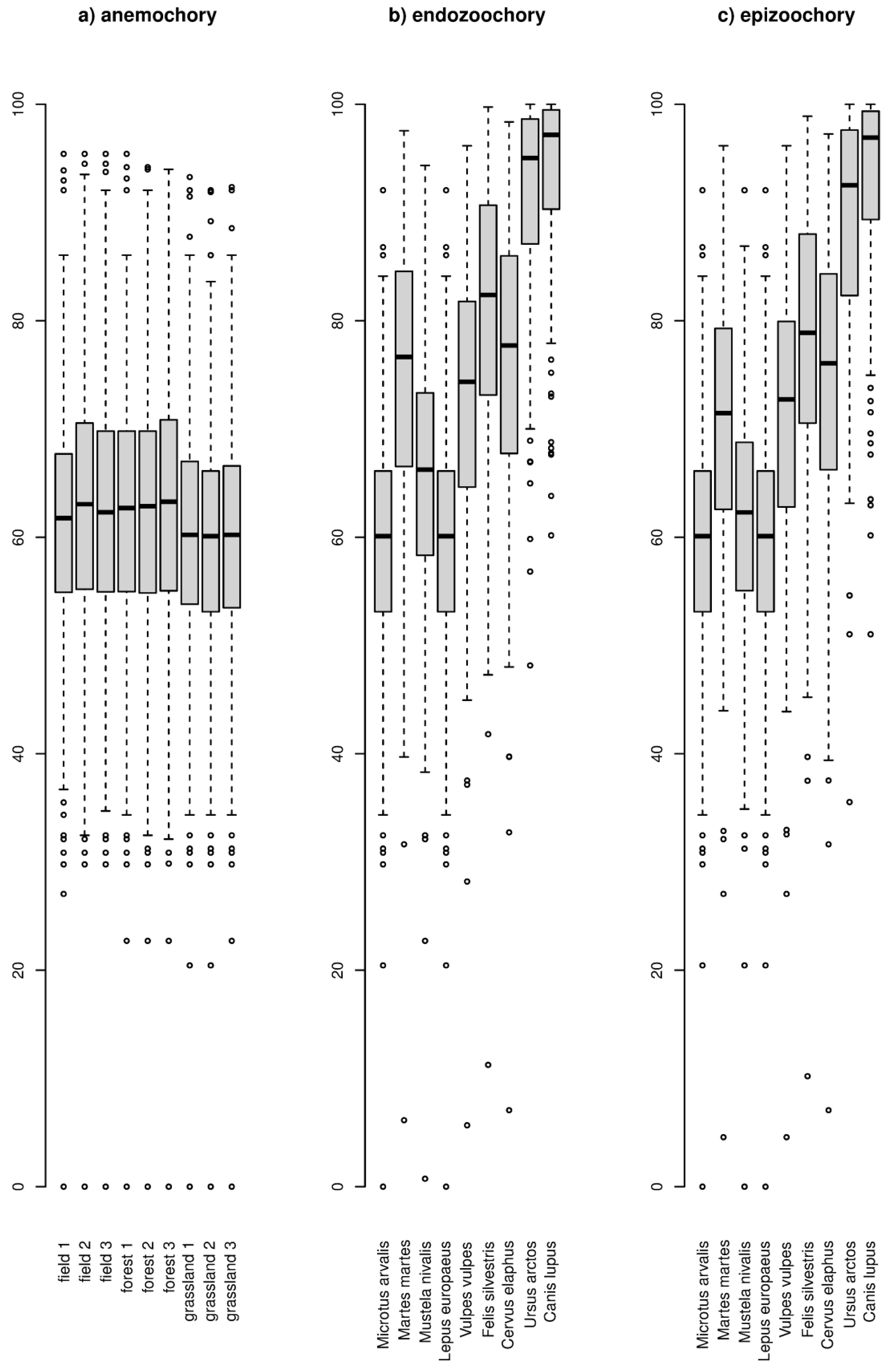

Supplement: Figure S6 — Percentage of the predicted future potential new range (on the example of the A1 CCCMA environmental model for 2080) that is reached assuming dispersal by wind and animals. Each boxplot represents 140 plant species (cf. fig. 5 in the main document). (TIFF) [file pone.0067909.s006.tiff]

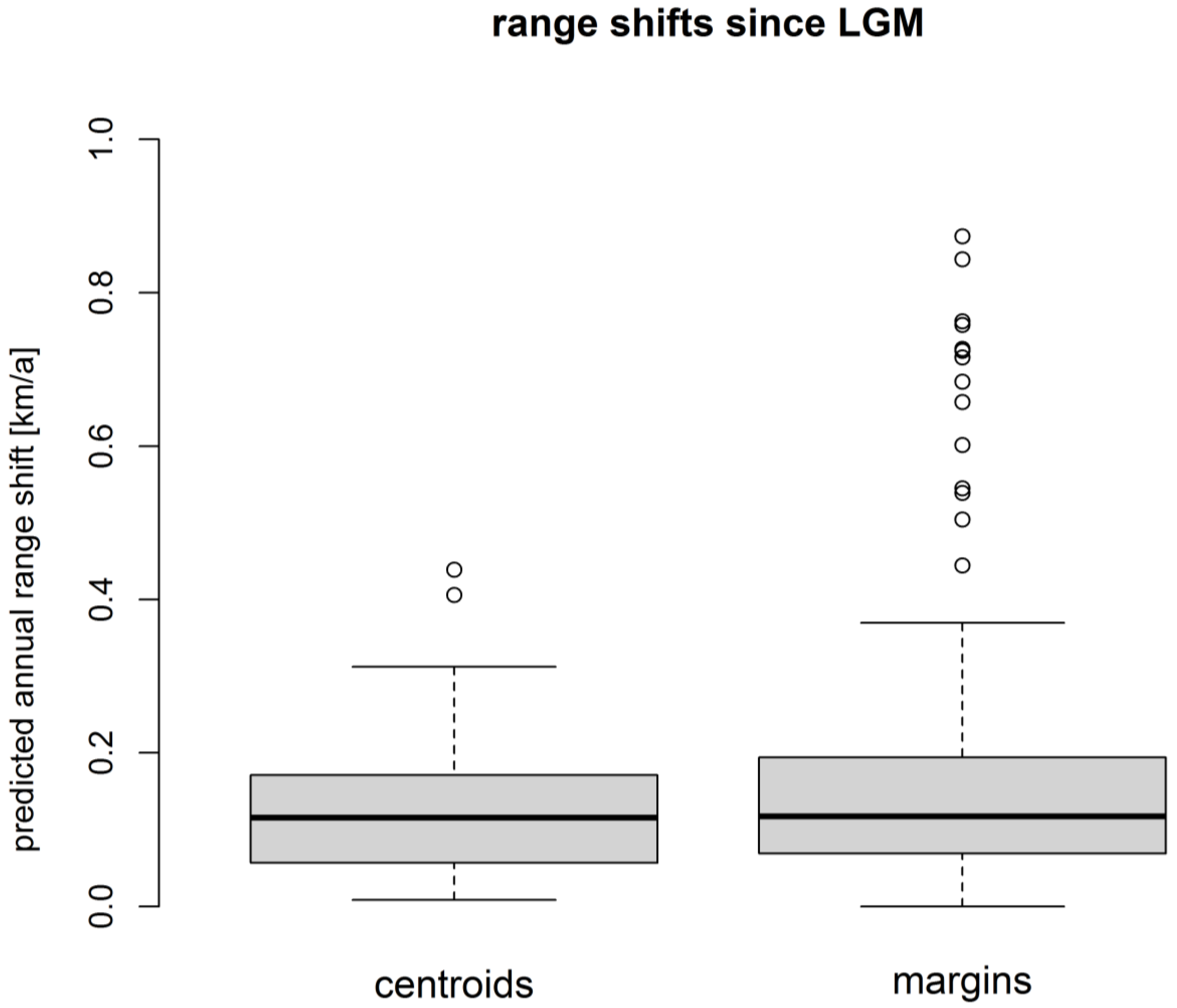

Supplement: Figure S7 — Modelled annual range shifts of the N = 140 European plant species since the Last Glacial Maximum (LGM) based on the distance between the centroids respectively on the range margins of the modelled past and current range. The period in which the species migrated to fulfil their current ranges was set to 10 000 years. (TIFF) [file pone.0067909.s007.tiff]

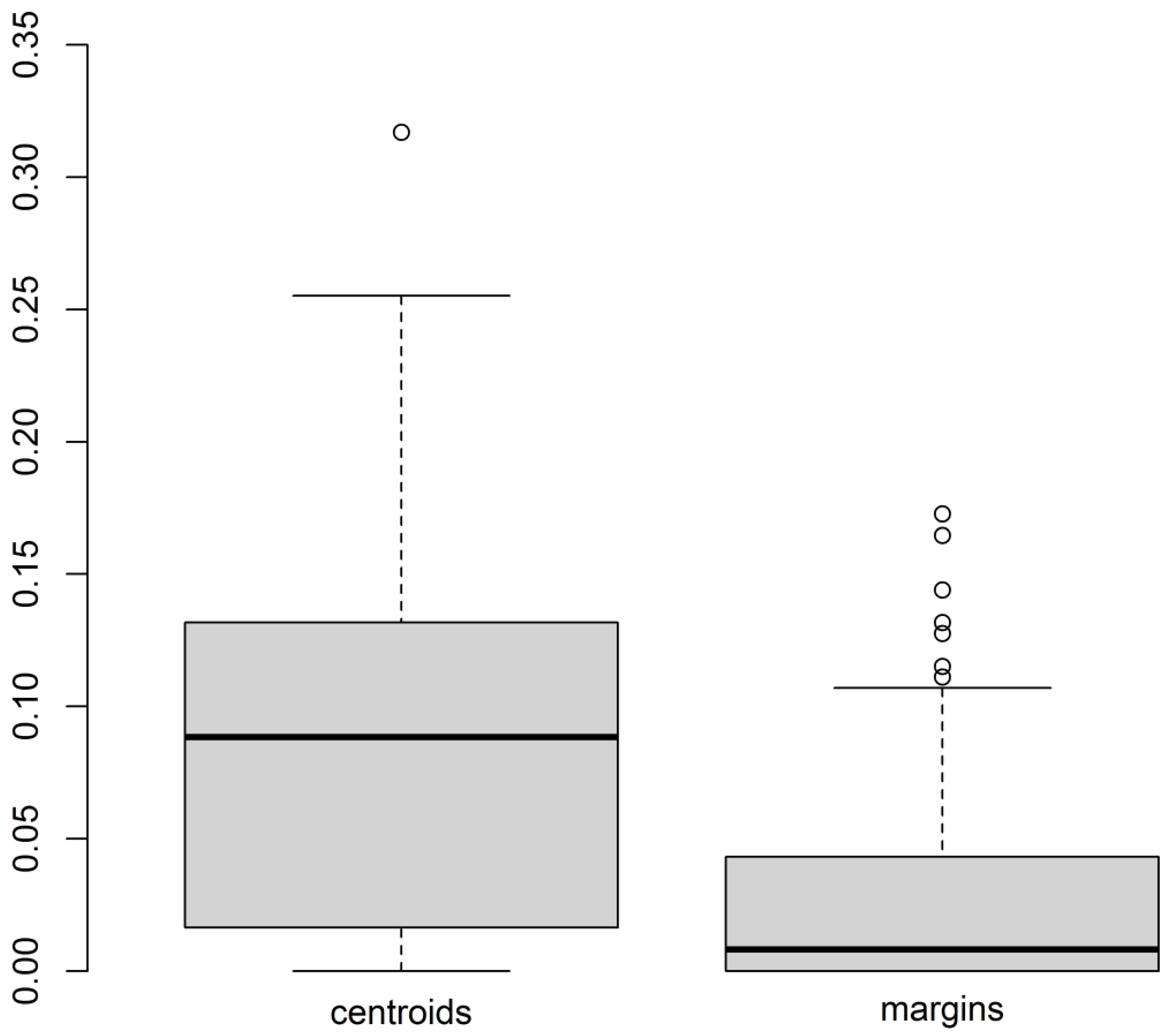

Supplement: Figure S8 — Proportion of the modelled migration rates that exceed the modelled annual range shifts based on the nine climatic models. Each boxplot represents for each of the N = 140 plant species 243 proportions ( = 27 dispersal modes * nine environmental models). (TIFF) [file pone.0067909.s008.tiff]

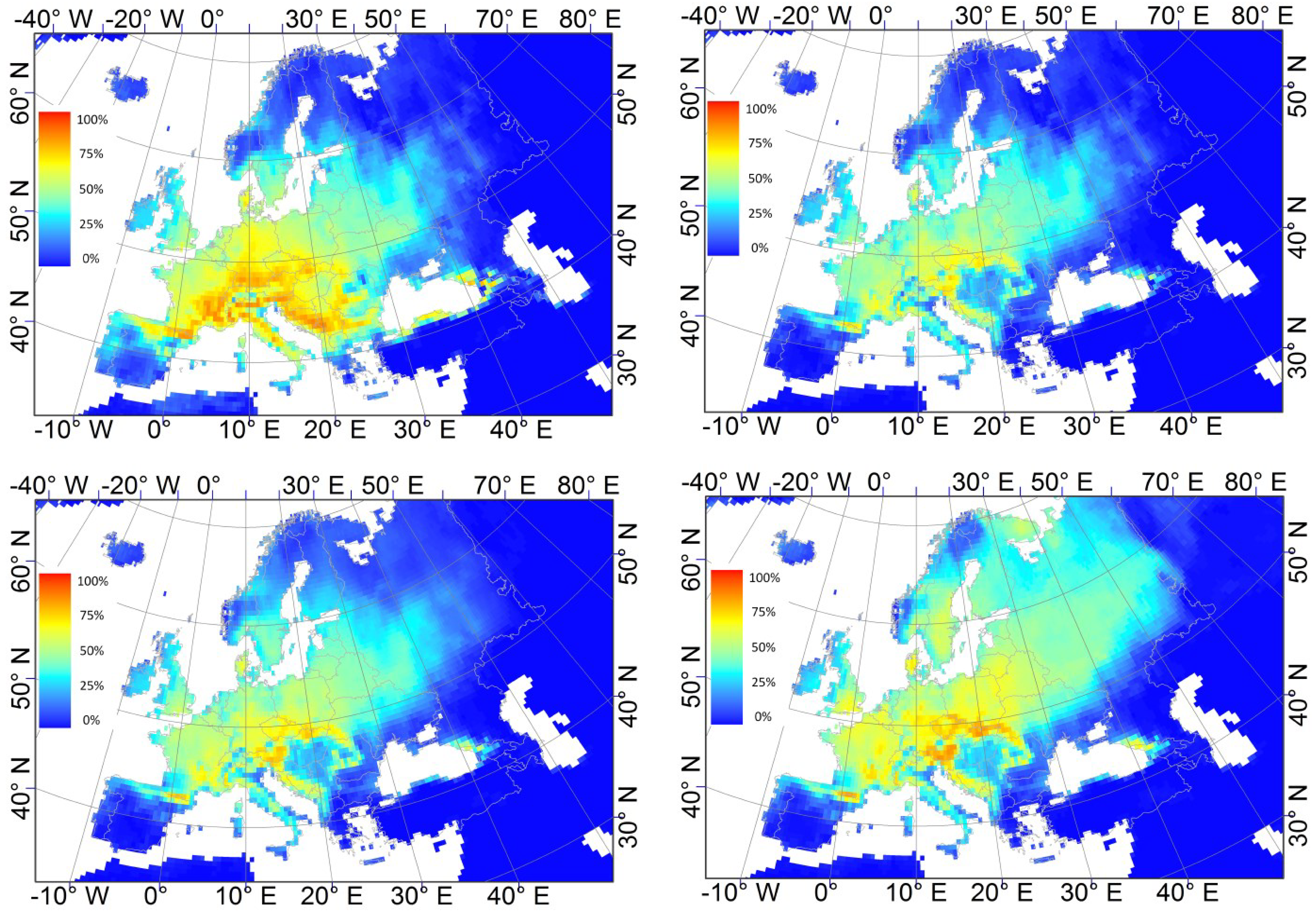

Supplement: Figure S9 — Distribution of biodiversity considering 140 species a) under current climatic conditions b) under future climatic conditions according to the A1 Scenario CCCMA for 2080 assuming no migration c) under future climatic conditions according to the A1 Scenario CCCMA for 2080 assuming “realistic” migration d) under future climatic conditions according to the A1 Scenario CCCMA for 2080 assuming full migration. For the no-migration map we only considered the overlaps between the current and the future ranges, for the realistic migration rate maps we took additionally the overlaps between a buffer of the estimated annual migration rates multiplied with 105 years around the current range and the future range into account. For the full migration map we considered the entire future ranges. a)b)d):100% means that all 140 species are predicted to occur at this place. c): 100% means that all 140 species are predicted to occur at this place and in terms of potentially new areas: the place is predicted to be reached by all 27 dispersal modes. Projection: Europe Albers Equal Area Conic. (TIFF) [file pone.0067909.s009.tiff]

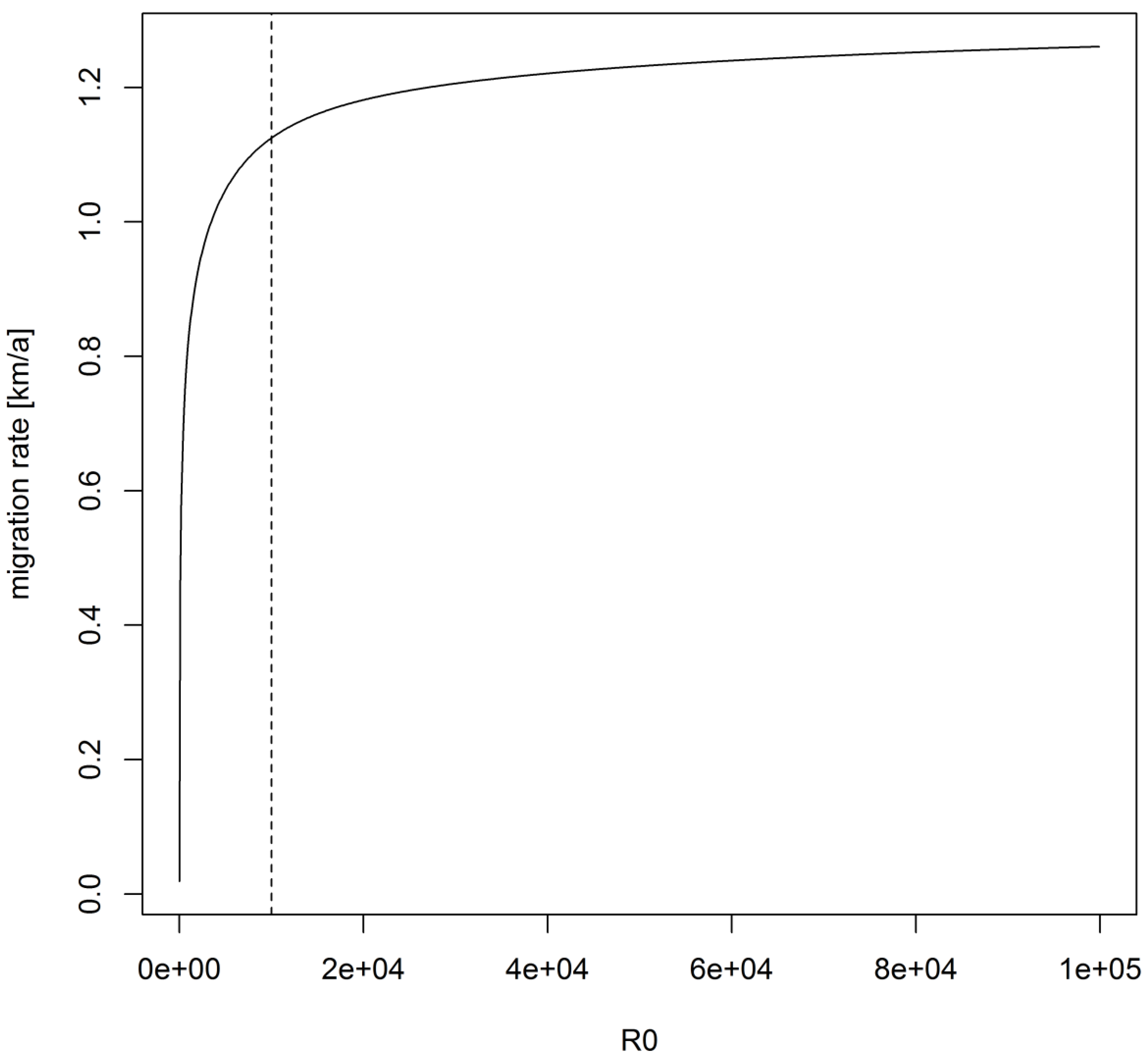

Supplement: Figure S10 — Sensitivity of the estimation of the migration rate to the net reproduction rate R0 on the example of Geum urbanum , dispersal by Cervus elaphus (epizoochorous). R0 used in this study is marked by the dotted line, resulting in a migration rate of 1.12 km/a. (TIFF) [file pone.0067909.s010.tiff]
