# Supplementary material for: Are Plant Species Able to Keep Pace with the Rapidly Changing Climate?
Source: PLoS One. 2013 Jul 24;8(7):e67909. doi: 10.1371/journal.pone.0067909 (PMC3722234; doi:10.1371/journal.pone.0067909)
Supplement: Table S1 — Compilation of parameters and traits for the 140 plant species considered in this study: Hrel (releasing height) and Vterm (terminal velocity) are parameters used to model the Anemochory kernels (cf. Tackenberg 2003). Data are taken form the D3-database (www.seed-dispersal.info). Retention potential (rtp.straight.hair), diaspore mass (dia.mass), morphology (dia.morph) and were taken from the D3-database (www.seed-dispersal.info). The age of first flowering (Age of FF) is estimated based on species life forms according to the CloPla data base (Klimešová & de Bello 2009). For eight of the 140 species considered in this study the parameters of the bi-exponential function (formula 1 in the main document) were experimentally determined (these species are marked with *). For the other 132 species the two most similar species (concerning diaspore mass, morphology and retention potential – cf. table S5) with experimentally determined parameters are given in this table (straighthair.spec1 and straighthair.spec2). f1 and f2 are the proportions of the retention times that are sampled from the bi-exponential distributions of the CDF for the prior species for that the CDF was empirically fitted (see table S5). (DOC) [file pone.0067909.s011.doc]

Table S1: Compilation of parameters and traits for the 140 plant species considered in this study: Hrel (releasing height) and Vterm (terminal velocity) are parameters used to model the Anemochory kernels (cf. Tackenberg 2003). Data are taken form the D3-database (www.seed-dispersal.info). Retention potential (rtp.straight.hair), diaspore mass (dia.mass), morphology (dia.morph) and were taken from the D3-database (www.seed-dispersal.info). The age of first flowering (Age of FF) is estimated based on species life forms according to the CloPla data base (Klimešová & de Bello 2009). For eight of the 140 species considered in this study the parameters of the bi-exponential function (formula 1 in the main document) were experimentally determined (these species are marked with *). For the other 132 species the two most similar species (concerning diaspore mass, morphology and retention potential – cf. table S5) with experimentally determined parameters are given in this table (straighthair.spec1 and straighthair.spec2). f1 and f2 are the proportions of the retention times that are sampled from the bi-exponential distributions of the CDF for the prior species for that the CDF was empirically fitted (see table 5).

| **No.** | Species | Hrel [m] | Vterm [m/s] | rtp.straight.hair | dia.mass | dia.morph | Age of FF [years] | straighthair.spec1 | f1 | straighthair.spec2 | f2 |
| --- | --- | --- | --- | --- | --- | --- | --- | --- | --- | --- | --- |
| **1** | Aconitum napellus L. s.l. | 1 | 3.77 | 0.04 | 3.51016 | no | 2 | Stellaria media agg. | 0.36 | Helianthemum nummularium s.l. (L.) Mill. | 0.64 |
| **2** | Adonis aestivalis L. | 0.43 | 8.12 | 0 | 11.76 | elongated | 1 | Briza media L. | 0.05 | Anthyllis vulneraria ssp. alpestris (Kit. ex Schult.) Asch. & Graebn. | 0.95 |
| **3** | Aethionema saxatile agg. | 0.15 | 3.8 | 0.22 | 0.26107 | elongated | 3 | Poa bulbosa L. | 0.53 | Solidago virgaurea ssp. minuta (L.) Arcang. | 0.47 |
| **4** | Alyssum alyssoides (L.) L. | 0.19 | 2.14 | 0.17 | 0.47 | no | 1 | Veronica officinalis L. | 0.88 | Saxifraga paniculata Mill. | 0.12 |
| **5** | Aquilegia einseleana F. W. Schultz | 0.28 | 2.87 | 0.12 | 0.82 | no | 2 | Thymus pulegioides s.l. L. | 0.79 | Sagina saginoides (L.) H. Karst. | 0.21 |
| **6** | Arabidopsis thaliana (L.) Heynh. | 0.18 | 1.03 | 0.47 | 0.02325 | no | 1 | Cynodon dactylon (L.) Pers. | 0.99 | Festuca valesiaca Schleich. ex Gaudin | 0.01 |
| **7** | Arabis alpina agg.* | 0.23 | 1.76 | 0.06 | 0.1764 | flat | 3 |  |  |  |  |
| **8** | Arabis caerulea All. | 0.07 | 1.29 | 0.23 | 0.24 | no | 3 | Sempervivum montanum L. | 0.66 | Clinopodium vulgare L. | 0.34 |
| **9** | Arabis hirsuta agg. | 0.49 | 1.41 | 0.08 | 0.10696 | flat | 1 | Plantago media L. | 0.9 | Epilobium anagallidifolium Lam. | 0.1 |
| **10** | Arabis sagittata (Bertol.) DC. | 0.58 | 1.41 | 0.08 | 0.0996 | flat | 2 | Plantago media L. | 0.4 | Epilobium anagallidifolium Lam. | 0.6 |
| **11** | Arabis turrita L. | 0.4 | 1.81 | 0.16 | 0.48432 | no | 2 | Soldanella pusilla Baumg. | 0.93 | Veronica officinalis L. | 0.07 |
| **12** | Arenaria serpyllifolia agg.* | 0.14 | 1.71 | 0.27 | 0.15539 | no | 1 |  |  |  |  |
| **13** | Aristolochia clematitis L. | 0.5 | 2.83 | 0.09 | 1.3 | no | 2 | Plantago media L. | 0.14 | Bartsia alpina L. | 0.86 |
| **14** | Atriplex littoralis L. | 0.6 | 3.02 | 0.06 | 2.2936 | elongated | 1 | Ranunculus acris L. | 0.17 | Bromus catharticus M. Vahl | 0.83 |
| **15** | Atriplex micrantha Ledeb. | 0.9 | 3.28 | 0.02 | 1.858 | flat | 1 | Gentiana punctata L. | 0.3 | Oxyria digyna (L.) Hill | 0.7 |
| **16** | Atriplex oblongifolia Waldst. & Kit. | 0.75 | 3.2 | 0.02 | 3.2364 | flat | 1 | Trifolium repens L. | 0.89 | Peucedanum ostruthium (L.) Koch | 0.11 |
| **17** | Atriplex patula L. | 0.55 | 4.11 | 0.12 | 0.8336 | elongated | 1 | Phleum rhaeticum (Humphries) Rauschert | 0.86 | Bromus intermedius Guss. | 0.14 |
| **18** | Barbarea stricta Andrz. | 0.8 | 3.12 | 0.21 | 0.287 | no | 2 | Achillea millefolium L. | 0.63 | Sempervivum montanum L. | 0.37 |
| **19** | Barbarea verna (Mill.) Asch. | 0.45 | 4.59 | 0.11 | 0.9396 | no | 2 | Thymus pulegioides s.l. L. | 0.88 | Aster bellidiastrum (L.) Scop. | 0.12 |
| **No.** | Species | Hrel [m] | Vterm [m/s] | rtp.straight.hair | dia.mass | dia.morph | Age of FF [years] | straighthair.spec1 | f1 | straighthair.spec2 | f2 |
| **20** | Berteroa incana (L.) DC. | 0.48 | 3.22 | 0.13 | 0.74 | no | 1 | Thymus pulegioides s.l. L. | 0.64 | Sagina saginoides (L.) H. Karst. | 0.36 |
| **21** | Biscutella laevigata L.* | 0.28 | 1.37 | 0.02 | 3.28095 | flat | 2 |  |  |  |  |
| **22** | Camelina microcarpa Andrz. | 0.45 | 2.23 | 0.2 | 0.31 | no | 1 | Achillea millefolium L. | 0.92 | Sempervivum montanum L. | 0.08 |
| **23** | Camelina sativa agg. | 0.48 | 4.55 | 0.13 | 0.73296 | no | 1 | Thymus pulegioides s.l. L. | 0.62 | Sagina saginoides (L.) H. Karst. | 0.38 |
| **24** | Capsella bursa-pastoris (L.) Medik. | 0.36 | 1.87 | 0.29 | 0.1276 | no | 1 | Clinopodium vulgare L. | 0.03 | Setaria viridis (L.) P. Beauv. | 0.97 |
| **25** | Cardamine alpina Willd. | 0.07 | 2.76 | 0.25 | 0.1872 | no | 2 | Clinopodium vulgare L. | 0.65 | Setaria viridis (L.) P. Beauv. | 0.35 |
| **26** | Cardamine amara L. | 0.35 | 3.4 | 0.2 | 0.30667 | no | 2 | Achillea millefolium L. | 0.88 | Sempervivum montanum L. | 0.12 |
| **27** | Cardamine hirsuta L. | 0.19 | 1.88 | 0.26 | 0.16 | no | 1 | Clinopodium vulgare L. | 0.4 | Setaria viridis (L.) P. Beauv. | 0.6 |
| **28** | Cardamine impatiens L. | 0.48 | 3 | 0.21 | 0.28096 | no | 2 | Achillea millefolium L. | 0.55 | Sempervivum montanum L. | 0.45 |
| **29** | Cardamine resedifolia L. | 0.09 | 1.96 | 0.29 | 0.1264 | no | 5 | Clinopodium vulgare L. | 0.01 | Setaria viridis (L.) P. Beauv. | 0.99 |
| **30** | Cardaminopsis arenosa (L.) Hayek | 0.28 | 1.44 | 0.31 | 0.1048 | no | 2 | Setaria viridis (L.) P. Beauv. | 0.68 | Calluna vulgaris (L.) Hull | 0.32 |
| **31** | Cardaminopsis petraea (L.) Hiitonen | 0.18 | 2.35 | 0.31 | 0.1 | no | 3 | Setaria viridis (L.) P. Beauv. | 0.6 | Calluna vulgaris (L.) Hull | 0.4 |
| **32** | Cerastium alpinum agg. | 0.1 | 2.44 | 0.22 | 0.26 | no | 3 | Achillea millefolium L. | 0.26 | Sempervivum montanum L. | 0.74 |
| **33** | Cerastium arvense L. | 0.18 | 2.47 | 0.23 | 0.2226 | no | 3 | Clinopodium vulgare L. | 0.92 | Setaria viridis (L.) P. Beauv. | 0.08 |
| **34** | Cerastium latifolium L. | 0.07 | 1.81 | 0.13 | 0.76 | no | 3 | Thymus pulegioides s.l. L. | 0.68 | Sagina saginoides (L.) H. Karst. | 0.32 |
| **35** | Cerastium uniflorum Clairv.* | 0.05 | 1.4 | 0.24 | 0.2156 | no | 3 |  |  |  |  |
| **36** | Chenopodium foliosum Asch. | 0.43 | 5.96 | 0.15 | 0.56 | no | 1 | Thymus pulegioides s.l. L. | 0.2 | Sagina saginoides (L.) H. Karst. | 0.8 |
| **37** | Chenopodium glaucum L. | 0.3 | 2.47 | 0.27 | 0.15 | hooked | 1 | Anthoxanthum alpinum Å. Löve & D. Löve | 0.62 | Anthoxanthum odoratum L. | 0.38 |
| **38** | Chenopodium murale L. | 0.38 | 4.39 | 0.14 | 0.62 | no | 1 | Thymus pulegioides s.l. L. | 0.37 | Sagina saginoides (L.) H. Karst. | 0.63 |
| **39** | Chenopodium polyspermum L. | 0.38 | 2.7 | 0.22 | 0.26 | no | 1 | Achillea millefolium L. | 0.26 | Sempervivum montanum L. | 0.74 |
| **40** | Chenopodium urbicum L. | 0.65 | 3.64 | 0.21 | 0.28 | no | 1 | Achillea millefolium L. | 0.54 | Sempervivum montanum L. | 0.46 |
| **41** | Cochlearia anglica L. | 0.25 | 3.76 | 0.2 | 0.3112 | no | 2 | Achillea millefolium L. | 0.94 | Sempervivum montanum L. | 0.06 |
| **42** | Conringia orientalis (L.) Dumort. | 0.3 | 6.12 | 0.07 | 2 | no | 1 | Hypericum perforatum L. | 0.07 | Silene exscapa All. | 0.93 |
| **43** | Consolida ajacis (L.) Schur | 0.65 | 5.3 | 0.02 | 1.8876 | flat | 1 | Gentiana punctata L. | 0.37 | Oxyria digyna (L.) Hill | 0.63 |
| **44** | Consolida regalis Gray | 0.3 | 5.04 | 0.03 | 1.09 | flat | 1 | Oxyria digyna (L.) Hill | 0.31 | Stellaria media agg. | 0.69 |
| **45** | Corispermum leptopterum (Asch.) Iljin | 0.35 | 3.55 | 0.02 | 2.1474 | flat | 1 | Gentiana punctata L. | 0.78 | Oxyria digyna (L.) Hill | 0.23 |
| **No.** | Species | Hrel [m] | Vterm [m/s] | rtp.straight.hair | dia.mass | dia.morph | Age of FF [years] | straighthair.spec1 | f1 | straighthair.spec2 | f2 |
| **46** | Coronopus squamatus (Forssk.) Asch. | 0.18 | 4.47 | 0.02 | 3.34892 | flat | 1 | Trifolium repens L. | 0.98 | Peucedanum ostruthium (L.) Koch | 0.02 |
| **47** | Corydalis solida (L.) Clairv. | 0.15 | 5 | 0.07 | 1.85607 | no | 5 | Silene exscapa All. | 0.58 | Plantago media L. | 0.42 |
| **48** | Descurainia sophia (L.) Prantl | 0.45 | 2.71 | 0.3 | 0.11293 | no | 1 | Setaria viridis (L.) P. Beauv. | 0.81 | Calluna vulgaris (L.) Hull | 0.19 |
| **49** | Dianthus armeria L. | 0.45 | 2.91 | 0.21 | 0.27 | no | 1 | Achillea millefolium L. | 0.4 | Sempervivum montanum L. | 0.6 |
| **50** | Dianthus carthusianorum L. | 0.3 | 3.3 | 0.13 | 0.774 | no | 2 | Thymus pulegioides s.l. L. | 0.71 | Sagina saginoides (L.) H. Karst. | 0.29 |
| **51** | Dianthus deltoides L. | 0.28 | 1.78 | 0.25 | 0.17815 | no | 3 | Clinopodium vulgare L. | 0.57 | Setaria viridis (L.) P. Beauv. | 0.43 |
| **52** | Dianthus superbus L. | 0.45 | 2.72 | 0.11 | 0.916 | no | 2 | Thymus pulegioides s.l. L. | 0.54 | Aster bellidiastrum (L.) Scop. | 0.46 |
| **53** | Dianthus sylvestris Wulfen | 0.23 | 3.59 | 0.14 | 0.6912 | no | 2 | Thymus pulegioides s.l. L. | 0.54 | Sagina saginoides (L.) H. Karst. | 0.46 |
| **54** | Diplotaxis muralis (L.) DC. | 0.38 | 3.55 | 0.23 | 0.2186 | no | 1 | Clinopodium vulgare L. | 0.89 | Setaria viridis (L.) P. Beauv. | 0.11 |
| **55** | Diplotaxis tenuifolia (L.) DC. | 0.55 | 4.05 | 0.22 | 0.26533 | no | 3 | Achillea millefolium L. | 0.34 | Sempervivum montanum L. | 0.66 |
| **56** | Draba dubia Suter | 0.09 | 2.22 | 0.27 | 0.153 | no | 3 | Clinopodium vulgare L. | 0.33 | Setaria viridis (L.) P. Beauv. | 0.67 |
| **57** | Draba fladnizensis Wulfen | 0.05 | 1.52 | 0.23 | 0.22 | no | 3 | Clinopodium vulgare L. | 0.9 | Setaria viridis (L.) P. Beauv. | 0.1 |
| **58** | Draba muralis L. | 0.2 | 1.64 | 0.31 | 0.09724 | no | 1 | Setaria viridis (L.) P. Beauv. | 0.55 | Calluna vulgaris (L.) Hull | 0.45 |
| **59** | Draba siliquosa M. Bieb. | 0.04 | 2.33 | 0.24 | 0.1984 | no | 3 | Clinopodium vulgare L. | 0.74 | Setaria viridis (L.) P. Beauv. | 0.26 |
| **60** | Drosera intermedia Hayne | 0.09 | 1.21 | 0.11 | 0.028 | flat | 2 | Arenaria serpyllifolia agg. | 0.98 | Veronica chamaedrys s.str. L. | 0.02 |
| **61** | Eranthis hyemalis (L.) Salisb. | 0.1 | 10.04 | 0.13 | 0.75 | no | 5 | Thymus pulegioides s.l. L. | 0.66 | Sagina saginoides (L.) H. Karst. | 0.34 |
| **62** | Eruca sativa Mill. | 0.23 | 6.5 | 0.07 | 1.8628 | no | 1 | Silene exscapa All. | 0.6 | Plantago media L. | 0.4 |
| **63** | Erucastrum gallicum (Willd.) O. E. Schulz | 0.45 | 4.61 | 0.25 | 0.18 | no | 1 | Clinopodium vulgare L. | 0.59 | Setaria viridis (L.) P. Beauv. | 0.41 |
| **64** | Erysimum crepidifolium Rchb. | 0.38 | 2.72 | 0.18 | 0.39 | no | 2 | Phyteuma betonicifolium Vill. | 0.7 | Sedum alpestre Vill. | 0.3 |
| **65** | Filipendula ulmaria (L.) Maxim. | 0.95 | 1.51 | 0.15 | 0.5949 | elongated | 2 | Bromus intermedius Guss. | 0.31 | Homogyne alpina (L.) Cass. | 0.69 |
| **66** | Filipendula vulgaris Moench | 0.45 | 1.7 | 0.13 | 0.69989 | elongated | 2 | Phleum rhaeticum (Humphries) Rauschert | 0.08 | Bromus intermedius Guss. | 0.92 |
| **67** | Geum montanum L. | 0.23 | 0.98 | 0.07 | 1.72907 | hooked | 2 | Festuca ovina agg. | 0.59 | Holcus lanatus L. | 0.41 |
| **68** | Geum rivale L. | 0.5 | 1.79 | 0.1 | 1.11557 | hooked | 2 | Armeria maritima ssp. elongata (Hoffm.) Bonnier | 0.35 | Cerastium uniflorum Clairv. | 0.65 |
| **69** | Geum urbanum L. | 0.55 | 2.09 | 0.06 | 2.07618 | hooked | 2 | Festuca ovina agg. | 0.69 | Silene exscapa All. | 0.31 |
| **70** | Glaucium flavum Crantz | 0.5 | 5.98 | 0.1 | 1.119 | no | 1 | Bartsia alpina L. | 0.11 | Cerastium uniflorum Clairv. | 0.89 |
| **71** | Herniaria glabra L. | 0.18 | 1.32 | 0.37 | 0.055 | no | 2 | Calluna vulgaris (L.) Hull | 0.47 | Bromus diandrus Roth | 0.53 |
| **No.** | Species | Hrel [m] | Vterm [m/s] | rtp.straight.hair | dia.mass | dia.morph | Age of FF [years] | straighthair.spec1 | f1 | straighthair.spec2 | f2 |
| **72** | Hornungia petraea (L.) Rchb. | 0.09 | 2 | 0.32 | 0.0896 | no | 1 | Setaria viridis (L.) P. Beauv. | 0.4 | Calluna vulgaris (L.) Hull | 0.6 |
| **73** | Iberis amara L. | 0.2 | 5.06 | 0.04 | 3.17 | no | 1 | Helianthemum nummularium s.l. (L.) Mill. | 0.74 | Myosotis alpestris F. W. Schmidt | 0.26 |
| **74** | Iberis umbellata L. | 0.28 | 5.49 | 0.07 | 1.74 | no | 1 | Silene exscapa All. | 0.17 | Plantago media L. | 0.83 |
| **75** | Lepidium graminifolium L. | 0.55 | 2.92 | 0.16 | 0.4976 | no | 2 | Arabis alpina agg. | 0.06 | Sagina saginoides (L.) H. Karst. | 0.94 |
| **76** | Lepidium latifolium L. | 0.75 | 2.27 | 0.09 | 1.27717 | no | 2 | Poa alpina L. | 0.79 | Bartsia alpina L. | 0.21 |
| **77** | Lepidium ruderale L. | 0.2 | 2.86 | 0.09 | 1.25 | no | 1 | Poa alpina L. | 0.04 | Bartsia alpina L. | 0.96 |
| **78** | Lychnis flos-cuculi L. | 0.55 | 2 | 0.28 | 0.14 | no | 2 | Clinopodium vulgare L. | 0.18 | Setaria viridis (L.) P. Beauv. | 0.82 |
| **79** | Lychnis viscaria L. | 0.45 | 1.55 | 0.36 | 0.06333 | no | 3 | Calluna vulgaris (L.) Hull | 0.4 | Festuca pratensis Huds. | 0.6 |
| **80** | Minuartia fastigiata (Sm.) Rchb. | 0.22 | 1.77 | 0.33 | 0.08 | no | 1 | Setaria viridis (L.) P. Beauv. | 0.19 | Calluna vulgaris (L.) Hull | 0.81 |
| **81** | Moehringia trinervia (L.) Clairv. | 0.2 | 3.74 | 0.22 | 0.264 | no | 1 | Achillea millefolium L. | 0.32 | Sempervivum montanum L. | 0.68 |
| **82** | Neslia paniculata s.l. (L.) Desv. | 0.48 | 8.32 | 0.04 | 3.02 | no | 1 | Helianthemum nummularium s.l. (L.) Mill. | 0.52 | Myosotis alpestris F. W. Schmidt | 0.48 |
| **83** | Nigella damascena L. | 0.23 | 5.34 | 0.05 | 2.83 | no | 1 | Helianthemum nummularium s.l. (L.) Mill. | 0.22 | Myosotis alpestris F. W. Schmidt | 0.78 |
| **84** | Papaver hybridum L. | 0.35 | 3.27 | 0.3 | 0.11 | no | 1 | Setaria viridis (L.) P. Beauv. | 0.77 | Calluna vulgaris (L.) Hull | 0.23 |
| **85** | Petrorhagia prolifera agg. | 0.25 | 2.85 | 0.18 | 0.38586 | no | 1 | Phyteuma betonicifolium Vill. | 0.55 | Sedum alpestre Vill. | 0.45 |
| **86** | Petrorhagia saxifraga (L.) Link | 0.23 | 2.35 | 0.28 | 0.135 | no | 3 | Clinopodium vulgare L. | 0.12 | Setaria viridis (L.) P. Beauv. | 0.88 |
| **87** | Polycnemum majus A. Braun | 0.15 | 3.81 | 0.12 | 0.9014 | elongated | 1 | Veronica chamaedrys s.str. L. | 0.29 | Aster bellidiastrum (L.) Scop. | 0.71 |
| **88** | Polygonum bistorta L. | 0.65 | 3.86 | 0.01 | 10 | flat | 5 | Briza media L. | 0.31 | Trifolium badium Schreb. | 0.69 |
| **89** | Polygonum hydropiper L. | 0.43 | 3.74 | 0.06 | 2.2791 | no | 1 | Myosotis alpestris F. W. Schmidt | 0.14 | Hypericum perforatum L. | 0.86 |
| **90** | Polygonum lapathifolium s.l. L. | 0.5 | 4.9 | 0.05 | 2.69533 | no | 1 | Myosotis alpestris F. W. Schmidt | 0.98 | Hypericum perforatum L. | 0.02 |
| **91** | Polygonum viviparum L. | 0.15 | 2.48 | 0.05 | 2.91081 | no | 2 | Helianthemum nummularium s.l. (L.) Mill. | 0.35 | Myosotis alpestris F. W. Schmidt | 0.65 |
| **92** | Potentilla aurea L. | 0.13 | 4.27 | 0.2 | 0.33 | no | 2 | Sedum alpestre Vill. | 0.26 | Achillea millefolium L. | 0.74 |
| **93** | Potentilla erecta (L.) Räusch. | 0.2 | 1.99 | 0.18 | 0.3795 | no | 2 | Phyteuma betonicifolium Vill. | 0.3 | Sedum alpestre Vill. | 0.7 |
| **94** | Potentilla palustris (L.) Scop. | 0.65 | 2.23 | 0.19 | 0.34 | no | 3 | Sedum alpestre Vill. | 0.45 | Achillea millefolium L. | 0.55 |
| **95** | Pulsatilla alpina agg. | 0.3 | 1.64 | 0.04 | 3.78297 | hooked | 2 | Ranunculus bulbosus L. | 0.5 | Ranunculus bulbosus L. | 0.5 |
| **96** | Pulsatilla pratensis (L.) Mill. | 0.25 | 1.69 | 0.05 | 2.8 | hooked | 2 | Ranunculus bulbosus L. | 0.51 | Festuca ovina agg. | 0.49 |
| **97** | Ranunculus ficaria L. | 0.15 | 4.8 | 0.07 | 1.94667 | no | 5 | Silene exscapa All. | 0.88 | Plantago media L. | 0.12 |
| **No.** | Species | Hrel [m] | Vterm [m/s] | rtp.straight.hair | dia.mass | dia.morph | Age of FF [years] | straighthair.spec1 | f1 | straighthair.spec2 | f2 |
| **98** | Ranunculus flammula L. | 0.43 | 2.98 | 0.2 | 0.3204 | hooked | 2 | Saxifraga bryoides L. | 0.62 | Sorghum halepense (L.) Pers. | 0.38 |
| **99** | Ranunculus glacialis L. | 0.1 | 1.27 | 0.2 | 0.33 | hooked | 2 | Saxifraga bryoides L. | 0.96 | Sorghum halepense (L.) Pers. | 0.04 |
| **100** | Ranunculus lanuginosus L. | 0.5 | 4.48 | 0.09 | 1.3 | hooked | 2 | Holcus lanatus L. | 0.87 | Doronicum clusii agg. | 0.13 |
| **101** | Ranunculus lingua L. | 1 | 4.75 | 0.06 | 2.4 | hooked | 2 | Ranunculus acris L. | 0.52 | Festuca ovina agg. | 0.48 |
| **102** | Ranunculus polyanthemos agg. | 0.48 | 3.53 | 0.06 | 2.06233 | hooked | 2 | Festuca ovina agg. | 0.6 | Silene exscapa All. | 0.4 |
| **103** | Ranunculus repens L. | 0.23 | 2.75 | 0.06 | 2.12777 | hooked | 2 | Hypericum perforatum L. | 0.08 | Festuca ovina agg. | 0.92 |
| **104** | Ranunculus sardous Crantz | 0.2 | 3.37 | 0.08 | 1.6145 | hooked | 1 | Festuca ovina agg. | 0.44 | Holcus lanatus L. | 0.56 |
| **105** | Rorippa amphibia (L.) Besser | 0.8 | 2.8 | 0.05 | 2.8326 | no | 2 | Helianthemum nummularium s.l. (L.) Mill. | 0.22 | Myosotis alpestris F. W. Schmidt | 0.78 |
| **106** | Rumex crispus L. | 0.9 | 1.75 | 0.02 | 2.16273 | flat | 2 | Gentiana punctata L. | 0.8 | Oxyria digyna (L.) Hill | 0.2 |
| **107** | Rumex obtusifolius L. | 0.85 | 1.9 | 0.02 | 2.266 | flat | 2 | Gentiana punctata L. | 0.95 | Oxyria digyna (L.) Hill | 0.05 |
| **108** | Salix hastata L.* | 0.75 | 0.13 | 0.27 | 0.14515 | elongated | 5 |  |  |  |  |
| **109** | Salix retusa agg. | 0.18 | 0.26 | 0.18 | 0.3936 | elongated | 3 | Agrostis capillaris L. | 0.76 | Melica transsilvanica Schur | 0.24 |
| **110** | Sanguisorba officinalis L. | 0.9 | 2.84 | 0.04 | 3.51456 | no | 2 | Stellaria media agg. | 0.38 | Helianthemum nummularium s.l. (L.) Mill. | 0.62 |
| **111** | Saxifraga biflora All. | 0.14 | 1.41 | 0.33 | 0.0812 | no | 3 | Setaria viridis (L.) P. Beauv. | 0.22 | Calluna vulgaris (L.) Hull | 0.78 |
| **112** | Saxifraga bryoides L.* | 0.05 | 1.08 | 0.45 | 0.0276 | hooked | 3 |  |  |  |  |
| **113** | Saxifraga granulata L. | 0.28 | 0.82 | 0.45 | 0.02675 | no | 2 | Calluna vulgaris (L.) Hull | 0.13 | Cynodon dactylon (L.) Pers. | 0.87 |
| **114** | Saxifraga paniculata Mill.* | 0.23 | 1.62 | 0.37 | 0.05507 | no | 3 |  |  |  |  |
| **115** | Scleranthus annuus agg. | 0.11 | 3.47 | 0.08 | 1.60667 | elongated | 1 | Epilobium anagallidifolium Lam. | 0.98 | Salix hastata L. | 0.02 |
| **116** | Scleranthus polycarpos L. | 0.09 | 2.55 | 0.07 | 1.87 | elongated | 1 | Bromus catharticus M. Vahl | 0.48 | Epilobium anagallidifolium Lam. | 0.52 |
| **117** | Sedum acre L. | 0.09 | 1.45 | 0.43 | 0.0325 | no | 3 | Calluna vulgaris (L.) Hull | 0.16 | Sclerochloa dura (L.) P. Beauv. | 0.84 |
| **118** | Sedum album L. | 0.14 | 0.96 | 0.46 | 0.025 | no | 3 | Calluna vulgaris (L.) Hull | 0.06 | Cynodon dactylon (L.) Pers. | 0.94 |
| **119** | Sedum alpestre Vill.* | 0.06 | 1.3 | 0.5 | 0.01808 | no | 3 |  |  |  |  |
| **120** | Sedum ochroleucum Chaix | 0.23 | 1.46 | 0.52 | 0.015 | no | 3 | Festuca guestfalica Boenn. ex Rchb. | 0.68 | Festuca puccinellii Parl. | 0.32 |
| **121** | Sedum telephium L. s.str. | 0.45 | 0.88 | 0.35 | 0.07 | no | 2 | Calluna vulgaris (L.) Hull | 0.86 | Festuca pratensis Huds. | 0.14 |
| **122** | Silene acaulis agg. | 0.02 | 3.34 | 0.2 | 0.3342 | no | 3 | Sedum alpestre Vill. | 0.34 | Achillea millefolium L. | 0.66 |
| **123** | Silene armeria L. | 0.38 | 1.64 | 0.28 | 0.13 | no | 1 | Clinopodium vulgare L. | 0.06 | Setaria viridis (L.) P. Beauv. | 0.94 |
| **No.** | Species | Hrel [m] | Vterm [m/s] | rtp.straight.hair | dia.mass | dia.morph | Age of FF [years] | straighthair.spec1 | f1 | straighthair.spec2 | f2 |
| **124** | Silene cretica L. | 0.4 | 6.03 | 0.12 | 0.9 | no | 1 | Thymus pulegioides s.l. L. | 0.27 | Aster bellidiastrum (L.) Scop. | 0.73 |
| **125** | Silene dichotoma Ehrh. | 0.5 | 4.92 | 0.13 | 0.7454 | no | 1 | Thymus pulegioides s.l. L. | 0.65 | Sagina saginoides (L.) H. Karst. | 0.35 |
| **126** | Silene gallica L. | 0.28 | 4.17 | 0.2 | 0.31 | no | 1 | Achillea millefolium L. | 0.92 | Sempervivum montanum L. | 0.08 |
| **127** | Silene tatarica (L.) Pers. | 0.45 | 3.78 | 0.26 | 0.17 | no | 2 | Clinopodium vulgare L. | 0.5 | Setaria viridis (L.) P. Beauv. | 0.5 |
| **128** | Silene vulgaris s.l. (Moench) Garcke | 0.33 | 3.71 | 0.16 | 0.49502 | no | 2 | Arabis alpina agg. | 0.01 | Sagina saginoides (L.) H. Karst. | 0.99 |
| **129** | Sisymbrium orientale L. | 0.5 | 2.89 | 0.25 | 0.18 | no | 1 | Clinopodium vulgare L. | 0.59 | Setaria viridis (L.) P. Beauv. | 0.41 |
| **130** | Spergula morisonii Boreau | 0.15 | 0.97 | 0.07 | 0.16 | flat | 1 | Silene exscapa All. | 0.93 | Plantago media L. | 0.07 |
| **131** | Spergula pentandra agg. | 0.14 | 0.97 | 0.07 | 0.16 | flat | 1 | Silene exscapa All. | 0.93 | Plantago media L. | 0.07 |
| **132** | Spergularia maritima (All.) Chiov. | 0.23 | 1.22 | 0.1 | 0.04373 | flat | 2 | Danthonia decumbens (L.) DC. | 0.88 | Epilobium fleischeri Hochst. | 0.12 |
| **133** | Spergularia salina J. Presl & C. Presl | 0.13 | 1.18 | 0.35 | 0.07083 | no | 2 | Calluna vulgaris (L.) Hull | 0.91 | Festuca pratensis Huds. | 0.09 |
| **134** | Stellaria graminea L. | 0.3 | 2.11 | 0.22 | 0.25037 | no | 2 | Achillea millefolium L. | 0.11 | Sempervivum montanum L. | 0.89 |
| **135** | Thalictrum aquilegiifolium L. | 0.8 | 3.66 | 0.02 | 2.5 | flat | 2 | Echium vulgare L. | 0.49 | Peucedanum ostruthium (L.) Koch | 0.51 |
| **136** | Thalictrum flavum L. | 0.7 | 3.92 | 0.03 | 1.178 | flat | 2 | Oxyria digyna (L.) Hill | 0.44 | Stellaria media agg. | 0.56 |
| **137** | Thalictrum lucidum L. | 0.9 | 3.55 | 0.04 | 0.767 | flat | 2 | Stellaria media agg. | 0.79 | Rumex acetosella s.l. L. | 0.21 |
| **138** | Thlaspi alliaceum L. | 0.4 | 5.63 | 0.14 | 0.67 | no | 1 | Thymus pulegioides s.l. L. | 0.49 | Sagina saginoides (L.) H. Karst. | 0.51 |
| **139** | Thlaspi arvense L. | 0.3 | 2.71 | 0.08 | 1.465 | no | 1 | Plantago media L. | 0.53 | Bartsia alpina L. | 0.47 |
| **140** | Thlaspi montanum L. | 0.15 | 4.74 | 0.14 | 0.67933 | no | 3 | Thymus pulegioides s.l. L. | 0.51 | Sagina saginoides (L.) H. Karst. | 0.49 |

**Rerferences for table S1:**

Klimešová, J. & Bello, F. de (2009). CLO-PLA: the database of clonal and bud bank traits of Central European flora§. *Journal of Vegetation Science*, 20, 511–516.

Tackenberg, O. (2003). Modeling Long-Distance Dispersal of Plant Diaspores by Wind. *Ecological Monographs*, 73, 173–189.
