# Supplementary material for: Are Plant Species Able to Keep Pace with the Rapidly Changing Climate?
Source: PLoS One. 2013 Jul 24;8(7):e67909. doi: 10.1371/journal.pone.0067909 (PMC3722234; doi:10.1371/journal.pone.0067909)
Supplement: Table S2 — IPCC scenarios (IPPC third Assessment Report data) and GCMs used for species distribution modelling (SDM). (DOC) [file pone.0067909.s012.doc]

**Table S2: IPCC scenarios (IPPC third Assessment Report data) and GCMs used for species distribution modelling (SDM).**

| Description | time | Scenario | GCM | Spatial resolution (download) | Provided by | Reference |
| --- | --- | --- | --- | --- | --- | --- |
| current | 1950-2000 | - | - | 5 Minutes | WorldClim global climate database www.worldclim.org | Hijmans *et al.* 2005 |
| past | ~21000 years BP | - | Paleoclimate Modelling Intercomparison Project Phase II CCSM | 2.5 Minutes | WorldClim global climate database www.worldclim.org | Otto-Bliesner *et al.* 2006 |
| A1 CCCMA | 2080 | A1 | the Coupled Global Climate Model CGCM (version 2 resp. 3.1) provided by the Canadian Centre for Climate Modelling and Analysis CCCMA | 5 Minutes | International Centre for Tropical Agriculture – CIAT (http://gisweb.ciat.cgiar.org/GCMPage/#). | e.g. Flato *et al.* 2000 |
| A1 CSIRO | 2080 | A1 | the CSIRO Atmospheric Research (MK 2 resp. MK 30) climate model | 5 Minutes | International Centre for Tropical Agriculture – CIAT (http://gisweb.ciat.cgiar.org/GCMPage/#). | Hirst *et al.* 1996 |
| A1 HadCM3 | 2080 | A1 | the Hadley Centre Coupled Model (version 3) HadCM3 developed at the Hadley Centre in the United Kingdom. | 5 Minutes | International Centre for Tropical Agriculture – CIAT (http://gisweb.ciat.cgiar.org/GCMPage/#). | Gordon *et al.* 2000; Pope *et al.* 2000 |
| A2 CCCMA | 2080 | A2 | the Coupled Global Climate Model CGCM (version 2 resp. 3.1) provided by the Canadian Centre for Climate Modelling and Analysis CCCMA | 5 Minutes | International Centre for Tropical Agriculture – CIAT (http://gisweb.ciat.cgiar.org/GCMPage/#). | e.g. Flato *et al.* 2000 |
| A2 CSIRO | 2080 | A2 | the CSIRO Atmospheric Research (MK 2 resp. MK 30) climate model | 5 Minutes | International Centre for Tropical Agriculture – CIAT (http://gisweb.ciat.cgiar.org/GCMPage/#). | Hirst *et al.* 1996 |
| A2 HadCM3 | 2080 | A2 | the Hadley Centre Coupled Model (version 3) HadCM3 developed at the Hadley Centre in the United Kingdom. | 5 Minutes | International Centre for Tropical Agriculture – CIAT (http://gisweb.ciat.cgiar.org/GCMPage/#). | Gordon *et al.* 2000; Pope *et al.* 2000 |
| B2 CCCMA | 2080 | B2 | the Coupled Global Climate Model CGCM (version 2 resp. 3.1) provided by the Canadian Centre for Climate Modelling and Analysis CCCMA | 5 Minutes | International Centre for Tropical Agriculture – CIAT (http://gisweb.ciat.cgiar.org/GCMPage/#). | e.g. Flato *et al.* 2000 |
| B2 CSIRO | 2080 | B2 | the CSIRO Atmospheric Research (MK 2 resp. MK 30) climate model | 5 Minutes | International Centre for Tropical Agriculture – CIAT (http://gisweb.ciat.cgiar.org/GCMPage/#). | Hirst *et al.* 1996 |
| B2 HadCM3 | 2080 | B2 | the Hadley Centre Coupled Model (version 3) HadCM3 developed at the Hadley Centre in the United Kingdom. | 5 Minutes | International Centre for Tropical Agriculture – CIAT (http://gisweb.ciat.cgiar.org/GCMPage/#). | Gordon *et al.* 2000; Pope *et al.* 2000 |

**Rerferences for table S2:**

Gordon, C., Cooper, C., Senior, C.A., Banks, H. & Gregory, J.M. (2000). The simulation of SST, sea ice extents and ocean heat transports in a version of the Hadley Centre coupled model without flux adjustments. *Climate Dynamics*, 16, 147–168.

Hijmans, R.J., Cameron, S.E., Parra, J.L., Jones, P.G. & Jarvis, A. (2005). Very high resolution interpolated climate surfaces for global land areas. *Int. J. Climatol*, 25, 1965–1978.

Hirst, A.C., Gordon, H.B. & O'Farrell, S.P. (1996). Global warming in a coupled climate model including oceanic eddy-induced advection. *Geophysical Research Letters*, 23, 3361–3364.

Flato, G.M., Boer, G.J., Lee, W.G., McFarlane, N.A., Ramsden, D. & Reader, M.C. *et al.* (2000). The Canadian Centre for Climate Modelling and Analysis Global Coupled Model and its Climate. *Climate Dynamics*, 16, 451–467.

Pope, V.D., Gallani, M.L., Rowntree, P.R. & Stratton, R.A. (2000). The impact of new physical parametrizations in the Hadley Centre climate model: HadAM3. *Climate Dynamics*, 16, 123–146.

Otto-Bliesner, B.L., Brady, E.C., Clauzet, G., Tomas, R., Levis, S. & Kothavala, Z. (2006). Last Glacial Maximum and Holocene Climate in CCSM3. *J. Climate*, 19, 2526–2544.
