# Supplementary material for: Are Plant Species Able to Keep Pace with the Rapidly Changing Climate?
Source: PLoS One. 2013 Jul 24;8(7):e67909. doi: 10.1371/journal.pone.0067909 (PMC3722234; doi:10.1371/journal.pone.0067909)
Supplement: Table S3 — List of the 19 environmental variables used for SDM (Hijmans et al. 2005). (DOC) [file pone.0067909.s013.doc]

Table S3: List of the 19 environmental variables used for SDM (Hijmans *et al.* 2005).

| Annual Mean Temperature |
| --- |
| Mean Diurnal Range (=Mean of monthly difference between maximum temperature and minimum temperature) |
| Isothermality (=Mean Diurnal Range / Temperature Annual Range * 100) |
| Temperature Seasonality (standard deviation *100) |
| Max Temperature of Warmest Month |
| Min Temperature of Coldest Month |
| Temperature Annual Range (Max Temperature of Warmest Month - Min Temperature of Coldest Month) |
| Mean Temperature of Wettest Quarter |
| Mean Temperature of Driest Quarter |
| Mean Temperature of Warmest Quarter |
| Mean Temperature of Coldest Quarter |
| Annual Precipitation |
| Precipitation of Wettest Month |
| Precipitation of Driest Month |
| Precipitation Seasonality (Coefficient of Variation) |
| Precipitation of Wettest Quarter |
| Precipitation of Driest Quarter |
| Precipitation of Warmest Quarter |
| Precipitation of Coldest Quarter |

**Rerferences for table S3:**

Hijmans, R.J., Cameron, S.E., Parra, J.L., Jones, P.G. & Jarvis, A. (2005). Very high resolution interpolated climate surfaces for global land areas. *Int. J. Climatol*, 25, 1965–1978.
