# Supplementary material for: Are Plant Species Able to Keep Pace with the Rapidly Changing Climate?
Source: PLoS One. 2013 Jul 24;8(7):e67909. doi: 10.1371/journal.pone.0067909 (PMC3722234; doi:10.1371/journal.pone.0067909)
Supplement: Table S4 — Dispersal-relevant animal traits for the nine model mammals. Given are trophic group, mean retention time of food in the gut (MRT), home-range size, day-ranges (i.e. daily distance travelled) and population density. Abbreviated references (in square brackets) are resolved immediately following this table and refer to MRT (1st number in brackets), home-range size (2nd), day-range size (3rd) and population density (4th). (Data mainly taken from Will 2008). (DOC) [file pone.0067909.s014.doc]

Table S4: Dispersal-relevant animal traits for the nine model mammals. Given are trophic group, mean retention time of food in the gut (MRT), home-range size, day-ranges (i.e. daily distance travelled) and population density. Abbreviated references (in square brackets) are resolved immediately following this table and refer to MRT (1st number in brackets), home-range size (2nd), day-range size (3rd) and population density (4th). (Data mainly taken from Will 2008).

| species name | trophic group | MRT [h] | home-range [ha] | day-range [km] | density [ha-1] | references |
| --- | --- | --- | --- | --- | --- | --- |
| Mustela nivalis | carnivore | 3.38 | 65 | 0.613 | 0.045 | [01][02][02][03] |
| Felis silvestris | carnivore | 13.00 | 2,127 | 4.11 | 0.0005 | [04][05][05][06] |
| Canis lupus | carnivore | 18.40 | 18,500 | 22.6 | 0.0003 | [07][08][08][09] |
| Microtus arvalis | herbivore | 8.30 | 0.02 | 0.02 | 65 | [10][11][11][12] |
| Lepus europaeus | herbivore | 13.40 | 21 | 0.172 | 0.146 | [04][13][14][13] |
| Cervus elaphus | herbivore | 45.00 | 666 | 3.4 | 0.05 | [15][16][16][17] |
| Martes martes | omnivore | 4.10 | 486 | 2.1 | 0.0054 | [18][19][20][21] |
| Vulpes vulpes | omnivore | 6.80 | 356 | 2.5 | 0.0118 | [22][23][24][17] |
| Ursus arctos | omnivore | 13.00 | 60,236 | 9.75 | 0.002 | [25][26][26][21] |

[01] - Short 1961

[02] - Jędrzejewski *et al.* 1995

[03] - Brandt & Lambin 2007; Gittleman 1985

[04] - Illius & Gordon 1992

[05] - Wittmer 2001

[06] - Steffen 2003; Hötzel 2005

[07] - Childs-Sanford & Angel 2006; Banta & Krinsky 1979

[08] - Jędrzejewski *et al.* 2007

[09] - Jędrzejewski *et al.* 2001; Gittleman 1985; Carbone *et al.* 2005

[10] - Warner 1981

[11] - Mackin-Rogalska 1981

[12] - Briner *et al.* 2005

[13] - Rühe & Hohmann 2004

[14] - Rühe *et al.* 2004

[15] - Milne *et al.* 1978 and Baker & Hobbs 1987

[16] - Licoppe 2006

[17] - Carbone *et al.* 2005

[18] - Hickey *et al.* 1999

[19] - Storch 1988

[20] - Zalewski & Jędrzejewski 2006

[21] - Carbone *et al.* 2005; Gittleman 1985

[22] - Varela & Bucher 2006

[23] - Goszczyński 2002

[24] - Vos 1995

[25] - Pritchard & Robbins 1990

[26] - Preatoni *et al.* 2005

**Rerferences for table S4:**

Baker, D.L. & Hobbs, N.T. (1987). Strategies of digestion: Digestive efficiency and retention time of forage diets in montane ungulates. *Canadian Journal of Zoology*, 65, 1978–1984.

Banta, C. & Krinsky, S. (1979). Sites of organic acid production and pattern of digesta movement in the gastrointestinal tract of dogs. *Journal of Nutrition*, 109, 1592–1600.

Brandt, M.J. & Lambin, X. (2007). Movement patterns of a specialist predator, the weasel *Mustela nivalis* exploiting asynchronous cyclic field vole Microtus agrestis populations. *Acta Theriol*, 52, 13–25.

Briner, T., Nentwig, W. & Airoldi, J.-P. (2005). Habitat quality of wildflower strips for common voles (Microtus arvalis) and its relevance for agriculture. *Agriculture, Ecosystems & Environment*, 105, 173–179.

Carbone, C., Cowlishaw, G., Isaac, N.J.B. & Rowcliffe, J.M. (2005). How Far Do Animals Go? Determinants of Day Range in Mammals. *Am Nat*, 165, 290–297.

Childs-Sanford, S.E. & Angel, C.R. (2006). Transit time and digestibility of two experimental diets in the maned wolf (*Chrysocyon brachyurus*) and domestic dog (*Canis lupus*). *Zoo Biol*, 25, 369–381.

Gittleman, J.L. (1985). Carnivore body size: Ecological and taxonomic correlates. *Oecologia*, 67, 540–554.

Goszczyński, J. (2002). Home ranges in red fox: territoriality diminishes with increasing area. *Acta Theriologica*, 47, 103–114.

Hickey, J.R., Flynn, R.W., Buskirk, S.W., Gerow, K.G. & Willson, M.F. (1999). An Evaluation of a Mammalian Predator, *Martes americana*, as a Disperser of Seeds. *Oikos*, 87, 499.

Hötzel, M. (2005). *Diploma Thesis,* University Bielefeld, Germany.

Illius, A.W. & Gordon, I.J. (1992). Modelling the nutritional ecology of ungulate herbivores: evolution of body size and competitive interactions. *Oecologia*, 89, 428–434.

Jędrzejewski, W., Schmidt, K., Theuerkauf, J., Jędrzejewska, B. & Kowalczyk, R. (2007). Territory size of wolves *Canis lupus*: linking local (Białowieża Primeval Forest, Poland) and Holarctic-scale patterns. *Ecography*, 30, 66–76.

Jędrzejewski, W., Schmidt, K., Theuerkauf, J., Jędrzejewska, B. & Okarma, H. (2001). Daily movements and territory use by radio-collared wolves (*Canis lupus*) in Bialowieza Primeval Forest in Poland. *Can. J. Zool*, 79, 1993–2004.

Jędrzejewski, W., Jędrzejewska, B. & Szymura, L. (1995). Weasel Population Response, Home Range, and Predation on Rodents in a Deciduous Forest in Poland. Ecology. *Ecology*, 76, 179–195.

Licoppe, A. (2006). The diurnal habitat used by red deer (*Cervus elaphus* L.) in the Haute Ardenne. *European Journal of Wildlife Research*, 52, 164–170.

Mackin-Rogalska, R. (1981). Spatial structure of rodent populations co-occurring in different crop fields. *Polish Ecological Studies*, 7, 213–227.

Milne, J.A., Macrae, J.C., Spence, A.M. & Wilson S. (1978). A comparison of the voluntary intake and digestion of a range of forages at different times of the year by the sheep and the red deer (*Cervus elaphus*). *British Journal of Nutrition*, 40, 347–357.

Preatoni, D., Mustoni, A., Martinoli, A., Carlini, E., Chiarenzi, B. & Chiozzini, S. *et al.* (2005). Conservation of brown bear in the Alps: space use and settlement behavior of reintroduced bears. *Acta Oecologica*, 28, 189–197.

Pritchard, G.T. & Robbins, C.T. (1990). Digestive and metabolic efficiencies of grizzly and black bears. *Can. J. Zool*, 68, 1645–1651.

Rühe, F., Fischbeck, I. & Rieger, A. (2004). Zum Einfluss von Habitatmerkmalen auf die Populations-dichte von Feldhasen (*Lepus europeus* PALLAS) in Agrargebieten Norddeutschlands. *Beiträge zur Jagd- und Wildforschung*, 29, 333–342.

Rühe, F. & Hohmann, U. (2004). Seasonal locomotion and home-range characteristics of European hares (*Lepus europaeus*) in an arable region in central Germany. *European Journal of Wildlife Research*, 50, 101–111.

Short, H.L. (1961). Food habits of a captive least weasel. *Journal of Mammalogy*, 42, 273–274.

Steffen, C. (2003). *Diploma Thesis,* Universty Kaiserslautern, Germany.

Storch, I. (1988). Zur Raumnutzung von Baummardern. *Zeitschrift für Jagdwissenschaft*, 34, 115–119.

Varela, O. & Bucher, E. (2006). Passage time, viability, and germination of seeds ingested by foxes. *Journal of Arid Environments*, 67, 566–578.

Vos, A. (1995). Population dynamics of the red fox (*Vulpes vulpes*) after the disappearance of rabies in county Garmisch-Partenkirchen, Germany 1987-1992. *Annales Zoologici Fennici*, 32, 93–97.

Warner, A. (1981). Rate of passage of digesta through the gut of mammals and birds. *Nutrition Abstracts and Reviews Series B*, 51, 789–820.

Wittmer, H.U. (2001). Home range size, movements, and habitat utilization of three male European wildcats (Felis silvestris Schreber, 1777) in Saarland and Rheinland-Pfalz (Germany). *Zeitschrift für Säugetierkunde - Mammalian Biology*, 66, 365–370.

Zalewski, A. & Jędrzejewski, W. (2006). Spatial organisation and dynamics of the pine marten *Martes martes* population in Białowieza Forest (E Poland) compared with other European woodlands. *Ecography*, 29, 31–43.
