# Supplementary material for: Are Plant Species Able to Keep Pace with the Rapidly Changing Climate?
Source: PLoS One. 2013 Jul 24;8(7):e67909. doi: 10.1371/journal.pone.0067909 (PMC3722234; doi:10.1371/journal.pone.0067909)
Supplement: Table S5 — Parameters and traits for 64 of the 103 plant species for those the bi-exponential function was empirically fitted. c1, c2 and c3 are the empirically determined parameters of the cumulative density function for epizoochory (formula 1 in the main document, with [t] = 1 min). The c1, c2 and c3 data represent means over several repetitions, all R2 with p <0.05. Only the species used for the mixture in this study (see table S1 are listed here. See table S9 for the raw values. (DOC) [file pone.0067909.s015.doc]

Table S5: Parameters and traits for 64 of the 103 plant species for those the bi-exponential function was empirically fitted. c1, c2 and c3 are the empirically determined parameters of the cumulative density function for epizoochory (formula 1 in the main document, with [t] = 1 min). The c1, c2 and c3 data represent means over several repetitions, all R^2^ with p <0.05. Only the species used for the mixture in this study (see table S1 are listed here. See table S9 for the raw values.

| Exp.Species* | rtp.straighthair | dia.mass | dia.morph | c1 | c2 | c3 | R^2^ |
| --- | --- | --- | --- | --- | --- | --- | --- |
| Achillea millefolium L. | 0.2 | 0.2818 | no | 0.192 | -0.009 | -2.416 | 0.99 |
| Agrostis capillaris L. | 0.1775 | 0.2833 | elongated | 0.274 | -0.007 | -2.088 | 0.96 |
| Anthoxanthum alpinum Å. Löve & D. Löve | 0.246 | 0.1414 | hooked | 0.246 | -0.009 | -2.297 | 0.97 |
| Anthoxanthum odoratum L. | 0.31 | 0.1519 | hooked | 0.326 | -0.007 | -2.154 | 0.98 |
| Anthyllis vulneraria ssp. alpestris (Kit. ex Schult.) Asch. & Graebn. | 0.00398 | 6.9644 | elongated | 0.015 | -0.037 | -5.781 | 0.87 |
| Arabis alpina agg. | 0.152 | 0.0645 | flat | 0.225 | -0.01 | -2.338 | 0.98 |
| Arenaria serpyllifolia agg. | 0.1075 | 0.15539 | no | 0.158 | -0.008 | -2.284 | 0.99 |
| Armeria maritima ssp. elongata (Hoffm.) Bonnier | 0.1 | 1.48256 | hooked | 0.26 | -0.012 | -2.716 | 0.97 |
| Aster bellidiastrum (L.) Scop. | 0.11746 | 0.2266 | elongated | 0.171 | -0.009 | -3.409 | 0.97 |
| Bartsia alpina L. | 0.09388 | 0.29597 | no | 0.079 | -0.009 | -3.168 | 0.98 |
| Briza media L. | 0.0025 | 0.80003 | flat | 0.04 | -0.055 | -3.587 | 0.94 |
| Bromus catharticus M. Vahl | 0.06 | 5.934 | elongated | 0.045 | -0.004 | -3.545 | 0.94 |
| Bromus diandrus Roth | 0.4 | 14.45 | elongated | 0.522 | -0.007 | -10.778 | 0.72 |
| Bromus intermedius Guss. | 0.136 | 4.28 | elongated | 0.233 | -0.009 | -4.078 | 0.98 |
| Calluna vulgaris (L.) Hull | 0.345 | 0.4262 | no | 0.389 | -0.011 | -2.038 | 0.94 |
| Cerastium uniflorum Clairv. | 0.102 | 0.2156 | no | 0.152 | -0.011 | -3.196 | 0.99 |
| Clinopodium vulgare L. | 0.2275 | 0.1722 | no | 0.239 | -0.007 | -2.691 | 0.93 |
| Cynodon dactylon (L.) Pers. | 0.468 | 0.2178 | elongated | 0.559 | -0.008 | -8.063 | 0.77 |
| Danthonia decumbens (L.) DC. | 0.096 | 1.53941 | elongated | 0.233 | -0.01 | -2.903 | 0.85 |
| Doronicum clusii agg. | 0.0999 | 0.8061 | hooked | 0.157 | -0.014 | -3.084 | 0.99 |
| Echium vulgare L. | 0.02 | 3.04359 | no | 0.06 | -0.001 | -4.703 | 0.8 |
| Epilobium anagallidifolium Lam. | 0.078 | 0.0706 | elongated | 0.108 | -0.008 | -2.738 | 0.97 |
| Epilobium fleischeri Hochst. | 0.09767 | 0.1638 | elongated | 0.136 | -0.013 | -3.103 | 0.99 |
| Festuca duvalii (St.-Yves) Stohr | 0.304 | 0.4712 | elongated | 0.488 | -0.01 | -8.048 | 0.87 |
| Festuca guestfalica Boenn. ex Rchb. | 0.5 | 0.4712 | elongated | 0.543 | -0.012 | -3.145 | 0.88 |
| Festuca ovina agg. | 0.0625 | 0.56738 | hooked | 0.116 | -0.006 | -3.17 | 0.98 |
| Festuca pratensis Huds. | 0.368 | 0.0631 | elongated | 0.536 | -0.01 | -10.831 | 0.87 |
| Festuca puccinellii Parl. | 0.552 | 0.9052 | elongated | 0.689 | -0.011 | -2.326 | 0.92 |
| Festuca valesiaca Schleich. ex Gaudin | 0.48 | 0.1194 | elongated | 0.599 | -0.006 | -3.099 | 0.94 |
| Gentiana punctata L. | 0.022 | 1.2988 | flat | 0.031 | -0.005 | -9.209 | 0.98 |
| Helianthemum nummularium s.l. (L.) Mill. | 0.04 | 0.98177 | no | 0.06 | -0.007 | -2.977 | 0.98 |
| Holcus lanatus L. | 0.09 | 0.38381 | hooked | 0.151 | -0.006 | -3.311 | 0.95 |
| Homogyne alpina (L.) Cass. | 0.152 | 0.1099 | elongated | 0.359 | -0.013 | -8.51 | 0.95 |
| Hypericum perforatum L. | 0.06 | 0.10678 | no | 0.167 | -0.01 | -2.781 | 0.97 |
| Melica transsilvanica Schur | 0.192 | 0.0784 | elongated | 0.232 | -0.004 | -2.245 | 0.43 |
| Myosotis alpestris F. W. Schmidt | 0.05 | 0.485 | no | 0.061 | -0.005 | -9.454 | 0.99 |
| Oxyria digyna (L.) Hill | 0.026 | 0.6107 | flat | 0.051 | -0.011 | -4.126 | 0.96 |
| Exp.Species* | rtp.straighthair | dia.mass | dia.morph | c1 | c2 | c3 | R^2^ |
| Peucedanum ostruthium (L.) Koch | 0.02194 | 1.54087 | flat | 0.098 | -0.009 | -3.708 | 0.93 |
| Phleum rhaeticum (Humphries) Rauschert | 0.11933 | 0.3648 | elongated | 0.167 | -0.011 | -5.961 | 0.99 |
| Phyteuma betonicifolium Vill. | 0.18 | 0.4184 | no | 0.209 | -0.006 | -2.993 | 0.95 |
| Plantago media L. | 0.075 | 0.34713 | no | 0.13 | -0.008 | -2.38 | 0.98 |
| Poa alpina L. | 0.092 | 0.4803 | elongated | 0.095 | -0.007 | -4.018 | 0.99 |
| Poa bulbosa L. | 0.208 | 3.3496 | elongated | 0.299 | -0.01 | -20 | 0.33 |
| Ranunculus acris L. | 0.05 | 1.47279 | elongated | 0.089 | -0.005 | -8.669 | 0.95 |
| Ranunculus bulbosus L. | 0.035 | 3.21361 | hooked | 0.056 | -0.007 | -3.578 | 0.98 |
| Rumex acetosella s.l. L. | 0.0475 | 0.40879 | flat | 0.127 | -0.008 | -2.542 | 0.98 |
| Sagina saginoides (L.) H. Karst. | 0.162 | 0.3004 | no | 0.209 | -0.009 | -2.28 | 0.99 |
| Salix hastata L. | 0.08776 | 0.14515 | elongated | 0.102 | -0.007 | -2.749 | 0.94 |
| Saxifraga bryoides L. | 0.196 | 0.4492 | hooked | 0.272 | -0.009 | -2.107 | 0.99 |
| Saxifraga paniculata Mill. | 0.174 | 0.3738 | no | 0.242 | -0.013 | -2.262 | 0.99 |
| Sclerochloa dura (L.) P. Beauv. | 0.448 | 0.85 | flat | 0.564 | -0.014 | -2.526 | 0.85 |
| Sedum alpestre Vill. | 0.186 | 0.496 | no | 0.216 | -0.011 | -2.468 | 1 |
| Sempervivum montanum L. | 0.224 | 0.4805 | no | 0.315 | -0.01 | -5.697 | 0.99 |
| Setaria viridis (L.) P. Beauv. | 0.288 | 0.1198 | no | 0.503 | -0.008 | -12.165 | 0.74 |
| Silene exscapa All. | 0.066 | 0.2026 | no | 0.125 | -0.013 | -2.533 | 0.98 |
| Soldanella pusilla Baumg. | 0.1636 | 0.3033 | no | 0.218 | -0.008 | -2.711 | 0.97 |
| Solidago virgaurea ssp. minuta (L.) Arcang. | 0.228 | 0.1566 | elongated | 0.243 | -0.009 | -1.86 | 0.98 |
| Sorghum halepense (L.) Pers. | 0.204 | 1.16 | hooked | 0.383 | 0 | -1.596 | 0.2 |
| Stellaria media agg. | 0.035 | 0.40647 | no | 0.089 | -0.009 | -3.743 | 0.99 |
| Thymus pulegioides s.l. L. | 0.1125 | 0.17267 | no | 0.16 | -0.009 | -2.979 | 0.98 |
| Trifolium badium Schreb. | 0.00992 | 0.9041 | no | 0.041 | -0.016 | -3.725 | 0.98 |
| Trifolium repens L. | 0.0175 | 0.64732 | no | 0.035 | -0.003 | -4.762 | 0.99 |
| Veronica chamaedrys s.str. L. | 0.1125 | 0.20827 | flat | 0.154 | -0.007 | -2.666 | 0.99 |
| Veronica officinalis L. | 0.165 | 0.2954 | no | 0.202 | -0.01 | -2.098 | 0.98 |
