# Supplementary material for: Are Plant Species Able to Keep Pace with the Rapidly Changing Climate?
Source: PLoS One. 2013 Jul 24;8(7):e67909. doi: 10.1371/journal.pone.0067909 (PMC3722234; doi:10.1371/journal.pone.0067909)
Supplement: Table S6 — p values for the Wilcoxon tests between the future range shifts of the centroids and the future range shifts of the range margins. For all environmental models the range shifts of the range margins are significantly higher than the range shifts of the centroids. (DOC) [file pone.0067909.s016.doc]

Table S6: p values for the Wilcoxon tests between the future range shifts of the centroids and the future range shifts of the range margins for the 140 species considered in this study. For all environmental models the range shifts of the range margins are significantly higher than the range shifts of the centroids.

| Scenario | GCM | p values |
| --- | --- | --- |
| A2 | CCCMA | 2.20E-16 |
| A2 | CSIRO | 2.68E-16 |
| A2 | HADCM3 | 2.20E-16 |
| A1 | CCCMA | 2.20E-16 |
| A1 | CSIRO | 2.20E-16 |
| A1 | HADCM3 | 2.20E-16 |
| B2 | CCCMA | 2.20E-16 |
| B2 | CSIRO | 2.68E-13 |
| B2 | HADCM3 | 2.20E-16 |
