# Supplementary material for: Are Plant Species Able to Keep Pace with the Rapidly Changing Climate?
Source: PLoS One. 2013 Jul 24;8(7):e67909. doi: 10.1371/journal.pone.0067909 (PMC3722234; doi:10.1371/journal.pone.0067909)
Supplement: Table S7 — p values for the Kruskal-Wallis Rank Sum Test between the future range shifts referring to different environmental models (cf. fig. 1 ). (DOC) [file pone.0067909.s017.doc]

Table S7: p values for the Kruskal-Wallis Rank Sum Test between the future range shifts referring to different environmental models (cf. fig. 1).

| Group | centroids | margins | Significant differences between: |
| --- | --- | --- | --- |
| A2 scenario | 1.11 E-04 | 2.55 E-02 | A2 CCCMA, A2 CSIRO and A2 HADCM3 |
| A1 scenario | 1.55E-06 | 2.54 E-02 | A1 CCCMA, A1 CSIRO and A1 HADCM3 |
| B2 scenario | 1.64 E-04 | 2.51 E-02 | B2 CCCMA, B2 CSIRO and B2 HADCM3 |
| CCCMA GCM | 1.34E-08 | 5.28E-06 | A2 CCCMA, A1 CCCMA and B2 CCCMA |
| CSIRO GCM | 7.98E-05 | 7.15E-03 | A2 CSIRO, A1 CSIRO and B2 CSIRO |
| HADCM3 GCM | 1.11E-10 | 4.14E-06 | A2 HADCM3, A1 HADCM3 and B2 HADCM3 |
