# Supplementary material for: Are Plant Species Able to Keep Pace with the Rapidly Changing Climate?
Source: PLoS One. 2013 Jul 24;8(7):e67909. doi: 10.1371/journal.pone.0067909 (PMC3722234; doi:10.1371/journal.pone.0067909)
Supplement: Table S8 — Percentage of species that are predicted to be able to fulfil their future range up to 90% for the respective dispersal mode. (DOC) [file pone.0067909.s018.doc]

Table S8: Percentage of species that are predicted to be able to fulfil their future range up to 90 % for the respective dispersal mode.

| Anemochory | | | | | | | | | |
| --- | --- | --- | --- | --- | --- | --- | --- | --- | --- |
| vector | field 1 | field 2 | field 3 | forest 1 | forest 2 | forest 3 | grassland 1 | grassland 2 | grassland 3 |
| percentage | 2.86 | 2.86 | 2.86 | 2.86 | 2.86 | 2.86 | 2.14 | 1.43 | 1.43 |
| Endozoochory | | | | | | | | | |
| vector | Microtus arvalis | Martes martes | Mustela nivalis | Lepus europaeus | Vulpes vulpes | Felis silvestris | Cervus elaphus | Ursus arctos | Canis  lupus |
| percentage | 0.71 | 12.14 | 0.71 | 0.71 | 7.86 | 28.57 | 15.71 | 67.14 | 75.71 |
| Epizoochory | | | | | | | | | |
| vector | Microtus arvalis | Martes martes | Mustela nivalis | Lepus europaeus | Vulpes vulpes | Felis silvestris | Cervus elaphus | Ursus arctos | Canis  lupus |
| percentage | 0.71 | 5.00 | 0.71 | 0.71 | 5.00 | 20.00 | 12.14 | 56.43 | 73.57 |
