# Supplementary material for: Correction: Are Plant Species Able to Keep Pace with the Rapidly Changing Climate?
Source: PLoS One. 2014 May 27;9(5):e99248. doi: 10.1371/journal.pone.0099248 (PMC4035339; doi:10.1371/journal.pone.0099248)
Supplement: Table S9 — Proportions still attached in cattle coat (prop_attach, measured values) after a certain time [min] for up to five repetitions (rep.) for the 64 species in table S10. We standardized the measured values by subtracting the minimum value and dividing by the range of the measured values. (DOCX) [file pone.0099248.s001.docx]

Table S9: Proportions still attached in cattle coat (prop_attach, measured values) after a certain time [min]

for up to five repetitions (rep.) for the 64 species in table S5. We standardized the measured values by

subtracting the minimum value and dividing by the range of the measured values.

species rep. time [min] prop_att reference

Achillea millefolium L. 1 0 1.000 original measurement

Achillea millefolium L. 1 1 0.114 original measurement

Achillea millefolium L. 1 5 0.071 original measurement

Achillea millefolium L. 1 10 0.057 original measurement

Achillea millefolium L. 1 30 0.057 original measurement

Achillea millefolium L. 1 60 0.029 original measurement

Achillea millefolium L. 1 120 0.014 original measurement

Achillea millefolium L. 1 180 0.014 original measurement

Achillea millefolium L. 1 360 0.000 original measurement

Achillea millefolium L. 1 1440 0.000 original measurement

Achillea millefolium L. 2 0 1.000 original measurement

Achillea millefolium L. 2 1 0.416 original measurement

Achillea millefolium L. 2 5 0.286 original measurement

Achillea millefolium L. 2 10 0.260 original measurement

Achillea millefolium L. 2 30 0.195 original measurement

Achillea millefolium L. 2 60 0.195 original measurement

Achillea millefolium L. 2 120 0.156 original measurement

Achillea millefolium L. 2 180 0.078 original measurement

Achillea millefolium L. 2 360 0.000 original measurement

Achillea millefolium L. 2 1440 0.000 original measurement

Achillea millefolium L. 3 0 1.000 original measurement

Achillea millefolium L. 3 1 0.197 original measurement

Achillea millefolium L. 3 5 0.141 original measurement

Achillea millefolium L. 3 10 0.127 original measurement

Achillea millefolium L. 3 30 0.099 original measurement

Achillea millefolium L. 3 60 0.070 original measurement

Achillea millefolium L. 3 120 0.042 original measurement

Achillea millefolium L. 3 180 0.014 original measurement

Achillea millefolium L. 3 360 0.000 original measurement

Achillea millefolium L. 3 1440 0.000 original measurement

Achillea millefolium L. 4 0 1.000 original measurement

Achillea millefolium L. 4 1 0.347 original measurement

Achillea millefolium L. 4 5 0.240 original measurement

Achillea millefolium L. 4 10 0.227 original measurement

Achillea millefolium L. 4 30 0.213 original measurement

Achillea millefolium L. 4 60 0.187 original measurement

Achillea millefolium L. 4 120 0.133 original measurement

Achillea millefolium L. 4 180 0.027 original measurement

Achillea millefolium L. 4 360 0.000 original measurement

Achillea millefolium L. 4 1440 0.000 original measurement

Agrostis capillaris L. 1 0 1.000 original measurement

Agrostis capillaris L. 1 1 0.233 original measurement

Agrostis capillaris L. 1 5 0.164 original measurement

Agrostis capillaris L. 1 10 0.137 original measurement

Agrostis capillaris L. 1 30 0.110 original measurement

Agrostis capillaris L. 1 60 0.027 original measurement

Agrostis capillaris L. 1 120 0.027 original measurement

Agrostis capillaris L. 1 180 0.014 original measurement

Agrostis capillaris L. 1 360 0.000 original measurement

Agrostis capillaris L. 1 1440 0.000 original measurement

Agrostis capillaris L. 2 0 1.000 original measurement

Agrostis capillaris L. 2 1 0.415 original measurement

Agrostis capillaris L. 2 5 0.287 original measurement

Agrostis capillaris L. 2 10 0.255 original measurement

Agrostis capillaris L. 2 30 0.223 original measurement

Agrostis capillaris L. 2 60 0.223 original measurement

Agrostis capillaris L. 2 120 0.213 original measurement

Agrostis capillaris L. 2 180 0.106 original measurement

Agrostis capillaris L. 2 360 0.000 original measurement

Agrostis capillaris L. 2 1440 0.000 original measurement

Agrostis capillaris L. 3 0 1.000 original measurement

Agrostis capillaris L. 3 1 0.455 original measurement

Agrostis capillaris L. 3 5 0.364 original measurement

Agrostis capillaris L. 3 10 0.338 original measurement

Agrostis capillaris L. 3 30 0.286 original measurement

Agrostis capillaris L. 3 60 0.260 original measurement

Agrostis capillaris L. 3 120 0.208 original measurement

Agrostis capillaris L. 3 180 0.143 original measurement

Agrostis capillaris L. 3 360 0.026 original measurement

Agrostis capillaris L. 3 1440 0.000 original measurement

Agrostis capillaris L. 4 0 1.000 original measurement

Agrostis capillaris L. 4 1 0.183 original measurement

Agrostis capillaris L. 4 5 0.118 original measurement

Agrostis capillaris L. 4 10 0.118 original measurement

Agrostis capillaris L. 4 30 0.043 original measurement

Agrostis capillaris L. 4 60 0.022 original measurement

Agrostis capillaris L. 4 120 0.000 original measurement

Agrostis capillaris L. 4 180 0.000 original measurement

Agrostis capillaris L. 4 360 0.000 original measurement

Agrostis capillaris L. 4 1440 0.000 original measurement

Anthoxanthum alpinum Á. Löve & D. Löve 1 0 1.000 Lutz (2004)

Anthoxanthum alpinum Á. Löve & D. Löv 1 1 0.228 Lutz (2004)

Anthoxanthum alpinum Á. Löve & D. Löve 1 5 0.139 Lutz (2004)

Anthoxanthum alpinum Á. Löve & D. Löve 1 10 0.127 Lutz (2004)

Anthoxanthum alpinum Á. Löve & D. Löve 1 30 0.114 Lutz (2004)

Anthoxanthum alpinum Á. Löve & D. Löve 1 60 0.114 Lutz (2004)

Anthoxanthum alpinum Á. Löve & D. Löve 1 120 0.051 Lutz (2004)

Anthoxanthum alpinum Á. Löve & D. Löve 1 180 0.025 Lutz (2004)

Anthoxanthum alpinum Á. Löve & D. Löve 1 360 0.000 Lutz (2004)

Anthoxanthum alpinum Á. Löve & D. Löve 1 1440 0.000 Lutz (2004)

Anthoxanthum alpinum Á. Löve & D. Löve 2 0 1.000 Lutz (2004)

Anthoxanthum alpinum Á. Löve & D. Löve 2 1 0.449 Lutz (2004)

Anthoxanthum alpinum Á. Löve & D. Löve 2 5 0.377 Lutz (2004)

Anthoxanthum alpinum Á. Löve & D. Löve 2 10 0.362 Lutz (2004)

Anthoxanthum alpinum Á. Löve & D. Löve 2 30 0.362 Lutz (2004)

Anthoxanthum alpinum Á. Löve & D. Löve 2 60 0.362 Lutz (2004)

Anthoxanthum alpinum Á. Löve & D. Löve 2 120 0.145 Lutz (2004)

Anthoxanthum alpinum Á. Löve & D. Löve 2 180 0.087 Lutz (2004)

Anthoxanthum alpinum Á. Löve & D. Löve 2 360 0.014 Lutz (2004)

Anthoxanthum alpinum Á. Löve & D. Löve 2 1440 0.000 Lutz (2004)

Anthoxanthum alpinum Á. Löve & D. Löve 3 0 1.000 Lutz (2004)

Anthoxanthum alpinum Á. Löve & D. Löve 3 1 0.426 Lutz (2004)

Anthoxanthum alpinum Á. Löve & D. Löve 3 5 0.255 Lutz (2004)

Anthoxanthum alpinum Á. Löve & D. Löve 3 10 0.223 Lutz (2004)

Anthoxanthum alpinum Á. Löve & D. Löve 3 30 0.191 Lutz (2004)

Anthoxanthum alpinum Á. Löve & D. Löve 3 60 0.181 Lutz (2004)

Anthoxanthum alpinum Á. Löve & D. Löve 3 120 0.043 Lutz (2004)

Anthoxanthum alpinum Á. Löve & D. Löve 3 180 0.043 Lutz (2004)

Anthoxanthum alpinum Á. Löve & D. Löve 3 360 0.011 Lutz (2004)

Anthoxanthum alpinum Á. Löve & D. Löve 3 1440 0.000 Lutz (2004)

Anthoxanthum alpinum Á. Löve & D. Löve 4 0 1.000 Lutz (2004)

Anthoxanthum alpinum Á. Löve & D. Löve 4 1 0.289 Lutz (2004)

Anthoxanthum alpinum Á. Löve & D. Löve 4 5 0.211 Lutz (2004)

Anthoxanthum alpinum Á. Löve & D. Löve 4 10 0.178 Lutz (2004)

Anthoxanthum alpinum Á. Löve & D. Löve 4 30 0.156 Lutz (2004)

Anthoxanthum alpinum Á. Löve & D. Löve 4 60 0.133 Lutz (2004)

Anthoxanthum alpinum Á. Löve & D. Löve 4 120 0.067 Lutz (2004)

Anthoxanthum alpinum Á. Löve & D. Löve 4 180 0.044 Lutz (2004)

Anthoxanthum alpinum Á. Löve & D. Löve 4 360 0.011 Lutz (2004)

Anthoxanthum alpinum Á. Löve & D. Löve 4 1440 0.000 Lutz (2004)

Anthoxanthum alpinum Á. Löve & D. Löve 5 0 1.000 Lutz (2004)

Anthoxanthum alpinum Á. Löve & D. Löve 5 1 0.212 Lutz (2004)

Anthoxanthum alpinum Á. Löve & D. Löve 5 5 0.141 Lutz (2004)

Anthoxanthum alpinum Á. Löve & D. Löve 5 10 0.129 Lutz (2004)

Anthoxanthum alpinum Á. Löve & D. Löve 5 30 0.129 Lutz (2004)

Anthoxanthum alpinum Á. Löve & D. Löve 5 60 0.118 Lutz (2004)

Anthoxanthum alpinum Á. Löve & D. Löve 5 120 0.047 Lutz (2004)

Anthoxanthum alpinum Á. Löve & D. Löve 5 180 0.035 Lutz (2004)

Anthoxanthum alpinum Á. Löve & D. Löve 5 360 0.024 Lutz (2004)

Anthoxanthum alpinum Á. Löve & D. Löve 5 1440 0.000 Lutz (2004)

Anthoxanthum odoratum L. 1 0 1.000 original measurement

Anthoxanthum odoratum L. 1 1 0.439 original measurement

Anthoxanthum odoratum L. 1 5 0.273 original measurement

Anthoxanthum odoratum L. 1 10 0.258 original measurement

Anthoxanthum odoratum L. 1 30 0.182 original measurement

Anthoxanthum odoratum L. 1 60 0.152 original measurement

Anthoxanthum odoratum L. 1 120 0.000 original measurement

Anthoxanthum odoratum L. 1 180 0.000 original measurement

Anthoxanthum odoratum L. 2 0 1.000 original measurement

Anthoxanthum odoratum L. 2 1 0.366 original measurement

Anthoxanthum odoratum L. 2 5 0.254 original measurement

Anthoxanthum odoratum L. 2 10 0.169 original measurement

Anthoxanthum odoratum L. 2 30 0.127 original measurement

Anthoxanthum odoratum L. 2 60 0.127 original measurement

Anthoxanthum odoratum L. 2 120 0.099 original measurement

Anthoxanthum odoratum L. 2 180 0.056 original measurement

Anthoxanthum odoratum L. 2 360 0.028 original measurement

Anthoxanthum odoratum L. 2 1440 0.000 original measurement

Anthoxanthum odoratum L. 3 0 1.000 original measurement

Anthoxanthum odoratum L. 3 1 0.462 original measurement

Anthoxanthum odoratum L. 3 5 0.436 original measurement

Anthoxanthum odoratum L. 3 10 0.385 original measurement

Anthoxanthum odoratum L. 3 30 0.321 original measurement

Anthoxanthum odoratum L. 3 60 0.269 original measurement

Anthoxanthum odoratum L. 3 120 0.192 original measurement

Anthoxanthum odoratum L. 3 180 0.141 original measurement

Anthoxanthum odoratum L. 3 360 0.051 original measurement

Anthoxanthum odoratum L. 3 1440 0.000 original measurement

Anthoxanthum odoratum L. 4 0 1.000 original measurement

Anthoxanthum odoratum L. 4 1 0.373 original measurement

Anthoxanthum odoratum L. 4 5 0.325 original measurement

Anthoxanthum odoratum L. 4 10 0.313 original measurement

Anthoxanthum odoratum L. 4 30 0.289 original measurement

Anthoxanthum odoratum L. 4 60 0.265 original measurement

Anthoxanthum odoratum L. 4 120 0.133 original measurement

Anthoxanthum odoratum L. 4 180 0.133 original measurement

Anthoxanthum odoratum L. 4 360 0.000 original measurement

Anthoxanthum odoratum L. 4 1440 0.000 original measurement

Anthyllis vulneraria ssp. alpestris (Kit. ex Schult.) Asch. & Graebn. 1 0 1.000 Lutz (2004)

Anthyllis vulneraria ssp. alpestris (Kit. ex Schult.) Asch. & Graebn. 1 1 0.022 Lutz (2004)

Anthyllis vulneraria ssp. alpestris (Kit. ex Schult.) Asch. & Graebn. 1 5 0.022 Lutz (2004)

Anthyllis vulneraria ssp. alpestris (Kit. ex Schult.) Asch. & Graebn. 1 10 0.022 Lutz (2004)

Anthyllis vulneraria ssp. alpestris (Kit. ex Schult.) Asch. & Graebn. 1 30 0.022 Lutz (2004)

Anthyllis vulneraria ssp. alpestris (Kit. ex Schult.) Asch. & Graebn. 1 60 0.022 Lutz (2004)

Anthyllis vulneraria ssp. alpestris (Kit. ex Schult.) Asch. & Graebn. 1 120 0.000 Lutz (2004)

Anthyllis vulneraria ssp. alpestris (Kit. ex Schult.) Asch. & Graebn. 1 180 0.000 Lutz (2004)

Anthyllis vulneraria ssp. alpestris (Kit. ex Schult.) Asch. & Graebn. 1 360 0.000 Lutz (2004)

Anthyllis vulneraria ssp. alpestris (Kit. ex Schult.) Asch. & Graebn. 1 1440 0.000 Lutz (2004)

Anthyllis vulneraria ssp. alpestris (Kit. ex Schult.) Asch. & Graebn. 2 0 1.000 Lutz (2004)

Anthyllis vulneraria ssp. alpestris (Kit. ex Schult.) Asch. & Graebn. 2 1 0.062 Lutz (2004)

Anthyllis vulneraria ssp. alpestris (Kit. ex Schult.) Asch. & Graebn. 2 5 0.050 Lutz (2004)

Anthyllis vulneraria ssp. alpestris (Kit. ex Schult.) Asch. & Graebn. 2 10 0.037 Lutz (2004)

Anthyllis vulneraria ssp. alpestris (Kit. ex Schult.) Asch. & Graebn. 2 30 0.012 Lutz (2004)

Anthyllis vulneraria ssp. alpestris (Kit. ex Schult.) Asch. & Graebn. 2 60 0.012 Lutz (2004)

Anthyllis vulneraria ssp. alpestris (Kit. ex Schult.) Asch. & Graebn. 2 120 0.000 Lutz (2004)

Anthyllis vulneraria ssp. alpestris (Kit. ex Schult.) Asch. & Graebn. 2 180 0.000 Lutz (2004)

Anthyllis vulneraria ssp. alpestris (Kit. ex Schult.) Asch. & Graebn. 2 360 0.000 Lutz (2004)

Anthyllis vulneraria ssp. alpestris (Kit. ex Schult.) Asch. & Graebn. 2 1440 0.000 Lutz (2004)

Anthyllis vulneraria ssp. alpestris (Kit. ex Schult.) Asch. & Graebn. 3 0 1.000 Lutz (2004)

Anthyllis vulneraria ssp. alpestris (Kit. ex Schult.) Asch. & Graebn. 3 1 0.014 Lutz (2004)

Anthyllis vulneraria ssp. alpestris (Kit. ex Schult.) Asch. & Graebn. 3 5 0.000 Lutz (2004)

Anthyllis vulneraria ssp. alpestris (Kit. ex Schult.) Asch. & Graebn. 3 10 0.000 Lutz (2004)

Anthyllis vulneraria ssp. alpestris (Kit. ex Schult.) Asch. & Graebn. 3 30 0.000 Lutz (2004)

Anthyllis vulneraria ssp. alpestris (Kit. ex Schult.) Asch. & Graebn. 3 60 0.000 Lutz (2004)

Anthyllis vulneraria ssp. alpestris (Kit. ex Schult.) Asch. & Graebn. 3 120 0.000 Lutz (2004)

Anthyllis vulneraria ssp. alpestris (Kit. ex Schult.) Asch. & Graebn. 3 180 0.000 Lutz (2004)

Anthyllis vulneraria ssp. alpestris (Kit. ex Schult.) Asch. & Graebn. 3 360 0.000 Lutz (2004)

Anthyllis vulneraria ssp. alpestris (Kit. ex Schult.) Asch. & Graebn. 3 1440 0.000 Lutz (2004)

Anthyllis vulneraria ssp. alpestris (Kit. ex Schult.) Asch. & Graebn. 4 0 1.000 Lutz (2004)

Anthyllis vulneraria ssp. alpestris (Kit. ex Schult.) Asch. & Graebn. 4 1 0.000 Lutz (2004)

Anthyllis vulneraria ssp. alpestris (Kit. ex Schult.) Asch. & Graebn. 4 5 0.000 Lutz (2004)

Anthyllis vulneraria ssp. alpestris (Kit. ex Schult.) Asch. & Graebn. 4 10 0.000 Lutz (2004)

Anthyllis vulneraria ssp. alpestris (Kit. ex Schult.) Asch. & Graebn. 4 30 0.000 Lutz (2004)

Anthyllis vulneraria ssp. alpestris (Kit. ex Schult.) Asch. & Graebn. 4 60 0.000 Lutz (2004)

Anthyllis vulneraria ssp. alpestris (Kit. ex Schult.) Asch. & Graebn. 4 120 0.000 Lutz (2004)

Anthyllis vulneraria ssp. alpestris (Kit. ex Schult.) Asch. & Graebn. 4 180 0.000 Lutz (2004)

Anthyllis vulneraria ssp. alpestris (Kit. ex Schult.) Asch. & Graebn. 4 360 0.000 Lutz (2004)

Anthyllis vulneraria ssp. alpestris (Kit. ex Schult.) Asch. & Graebn. 4 1440 0.000 Lutz (2004)

Anthyllis vulneraria ssp. alpestris (Kit. ex Schult.) Asch. & Graebn. 5 0 1.000 Lutz (2004)

Anthyllis vulneraria ssp. alpestris (Kit. ex Schult.) Asch. & Graebn. 5 1 0.000 Lutz (2004)

Anthyllis vulneraria ssp. alpestris (Kit. ex Schult.) Asch. & Graebn. 5 5 0.000 Lutz (2004)

Anthyllis vulneraria ssp. alpestris (Kit. ex Schult.) Asch. & Graebn. 5 10 0.000 Lutz (2004)

Anthyllis vulneraria ssp. alpestris (Kit. ex Schult.) Asch. & Graebn. 5 30 0.000 Lutz (2004)

Anthyllis vulneraria ssp. alpestris (Kit. ex Schult.) Asch. & Graebn. 5 60 0.000 Lutz (2004)

Anthyllis vulneraria ssp. alpestris (Kit. ex Schult.) Asch. & Graebn. 5 120 0.000 Lutz (2004)

Anthyllis vulneraria ssp. alpestris (Kit. ex Schult.) Asch. & Graebn. 5 180 0.000 Lutz (2004)

Anthyllis vulneraria ssp. alpestris (Kit. ex Schult.) Asch. & Graebn. 5 360 0.000 Lutz (2004)

Anthyllis vulneraria ssp. alpestris (Kit. ex Schult.) Asch. & Graebn. 5 1440 0.000 Lutz (2004)

Arabis alpina agg. 1 0 1.000 Lutz (2004)

Arabis alpina agg. 1 1 0.446 Lutz (2004)

Arabis alpina agg. 1 5 0.365 Lutz (2004)

Arabis alpina agg. 1 10 0.351 Lutz (2004)

Arabis alpina agg. 1 30 0.338 Lutz (2004)

Arabis alpina agg. 1 60 0.338 Lutz (2004)

Arabis alpina agg. 1 120 0.203 Lutz (2004)

Arabis alpina agg. 1 180 0.149 Lutz (2004)

Arabis alpina agg. 1 360 0.027 Lutz (2004)

Arabis alpina agg. 1 1440 0.000 Lutz (2004)

Arabis alpina agg. 2 0 1.000 Lutz (2004)

Arabis alpina agg. 2 1 0.348 Lutz (2004)

Arabis alpina agg. 2 5 0.228 Lutz (2004)

Arabis alpina agg. 2 10 0.207 Lutz (2004)

Arabis alpina agg. 2 30 0.174 Lutz (2004)

Arabis alpina agg. 2 60 0.174 Lutz (2004)

Arabis alpina agg. 2 120 0.087 Lutz (2004)

Arabis alpina agg. 2 180 0.054 Lutz (2004)

Arabis alpina agg. 2 360 0.022 Lutz (2004)

Arabis alpina agg. 2 1440 0.000 Lutz (2004)

Arabis alpina agg. 3 0 1.000 Lutz (2004)

Arabis alpina agg. 3 1 0.283 Lutz (2004)

Arabis alpina agg. 3 5 0.185 Lutz (2004)

Arabis alpina agg. 3 10 0.185 Lutz (2004)

Arabis alpina agg. 3 30 0.152 Lutz (2004)

Arabis alpina agg. 3 60 0.130 Lutz (2004)

Arabis alpina agg. 3 120 0.022 Lutz (2004)

Arabis alpina agg. 3 180 0.000 Lutz (2004)

Arabis alpina agg. 3 360 0.000 Lutz (2004)

Arabis alpina agg. 3 1440 0.000 Lutz (2004)

Arabis alpina agg. 4 0 1.000 Lutz (2004)

Arabis alpina agg. 4 1 0.141 Lutz (2004)

Arabis alpina agg. 4 5 0.064 Lutz (2004)

Arabis alpina agg. 4 10 0.051 Lutz (2004)

Arabis alpina agg. 4 30 0.051 Lutz (2004)

Arabis alpina agg. 4 60 0.051 Lutz (2004)

Arabis alpina agg. 4 120 0.000 Lutz (2004)

Arabis alpina agg. 4 180 0.000 Lutz (2004)

Arabis alpina agg. 4 360 0.000 Lutz (2004)

Arabis alpina agg. 4 1440 0.000 Lutz (2004)

Arabis alpina agg. 5 0 1.000 Lutz (2004)

Arabis alpina agg. 5 1 0.155 Lutz (2004)

Arabis alpina agg. 5 5 0.083 Lutz (2004)

Arabis alpina agg. 5 10 0.083 Lutz (2004)

Arabis alpina agg. 5 30 0.083 Lutz (2004)

Arabis alpina agg. 5 60 0.048 Lutz (2004)

Arabis alpina agg. 5 120 0.012 Lutz (2004)

Arabis alpina agg. 5 180 0.000 Lutz (2004)

Arabis alpina agg. 5 360 0.000 Lutz (2004)

Arabis alpina agg. 5 1440 0.000 Lutz (2004)

Arenaria serpyllifolia agg. 1 0 1.000 original measurement

Arenaria serpyllifolia agg. 1 1 0.083 original measurement

Arenaria serpyllifolia agg. 1 5 0.028 original measurement

Arenaria serpyllifolia agg. 1 10 0.028 original measurement

Arenaria serpyllifolia agg. 1 30 0.014 original measurement

Arenaria serpyllifolia agg. 1 60 0.014 original measurement

Arenaria serpyllifolia agg. 1 120 0.014 original measurement

Arenaria serpyllifolia agg. 1 180 0.014 original measurement

Arenaria serpyllifolia agg. 1 360 0.000 original measurement

Arenaria serpyllifolia agg. 1 1440 0.000 original measurement

Arenaria serpyllifolia agg. 2 0 1.000 original measurement

Arenaria serpyllifolia agg. 2 1 0.223 original measurement

Arenaria serpyllifolia agg. 2 5 0.149 original measurement

Arenaria serpyllifolia agg. 2 10 0.149 original measurement

Arenaria serpyllifolia agg. 2 30 0.117 original measurement

Arenaria serpyllifolia agg. 2 60 0.096 original measurement

Arenaria serpyllifolia agg. 2 120 0.096 original measurement

Arenaria serpyllifolia agg. 2 180 0.011 original measurement

Arenaria serpyllifolia agg. 2 360 0.011 original measurement

Arenaria serpyllifolia agg. 2 1440 0.000 original measurement

Arenaria serpyllifolia agg. 3 0 1.000 original measurement

Arenaria serpyllifolia agg. 3 1 0.250 original measurement

Arenaria serpyllifolia agg. 3 5 0.167 original measurement

Arenaria serpyllifolia agg. 3 10 0.107 original measurement

Arenaria serpyllifolia agg. 3 30 0.071 original measurement

Arenaria serpyllifolia agg. 3 60 0.060 original measurement

Arenaria serpyllifolia agg. 3 120 0.048 original measurement

Arenaria serpyllifolia agg. 3 180 0.036 original measurement

Arenaria serpyllifolia agg. 3 360 0.012 original measurement

Arenaria serpyllifolia agg. 3 1440 0.000 original measurement

Arenaria serpyllifolia agg. 4 0 1.000 original measurement

Arenaria serpyllifolia agg. 4 1 0.415 original measurement

Arenaria serpyllifolia agg. 4 5 0.287 original measurement

Arenaria serpyllifolia agg. 4 10 0.266 original measurement

Arenaria serpyllifolia agg. 4 30 0.213 original measurement

Arenaria serpyllifolia agg. 4 60 0.170 original measurement

Arenaria serpyllifolia agg. 4 120 0.106 original measurement

Arenaria serpyllifolia agg. 4 180 0.064 original measurement

Arenaria serpyllifolia agg. 4 360 0.011 original measurement

Arenaria serpyllifolia agg. 4 1440 0.000 original measurement

Armeria maritima ssp. elongata (Hoffm.) Bonnier 1 0 1.000 original measurement

Armeria maritima ssp. elongata (Hoffm.) Bonnier 1 1 0.370 original measurement

Armeria maritima ssp. elongata (Hoffm.) Bonnier 1 5 0.278 original measurement

Armeria maritima ssp. elongata (Hoffm.) Bonnier 1 10 0.222 original measurement

Armeria maritima ssp. elongata (Hoffm.) Bonnier 1 30 0.185 original measurement

Armeria maritima ssp. elongata (Hoffm.) Bonnier 1 60 0.148 original measurement

Armeria maritima ssp. elongata (Hoffm.) Bonnier 1 120 0.019 original measurement

Armeria maritima ssp. elongata (Hoffm.) Bonnier 1 180 0.000 original measurement

Armeria maritima ssp. elongata (Hoffm.) Bonnier 2 0 1.000 original measurement

Armeria maritima ssp. elongata (Hoffm.) Bonnier 2 1 0.036 original measurement

Armeria maritima ssp. elongata (Hoffm.) Bonnier 2 5 0.018 original measurement

Armeria maritima ssp. elongata (Hoffm.) Bonnier 2 10 0.018 original measurement

Armeria maritima ssp. elongata (Hoffm.) Bonnier 2 30 0.018 original measurement

Armeria maritima ssp. elongata (Hoffm.) Bonnier 2 60 0.018 original measurement

Armeria maritima ssp. elongata (Hoffm.) Bonnier 2 120 0.000 original measurement

Armeria maritima ssp. elongata (Hoffm.) Bonnier 2 180 0.000 original measurement

Armeria maritima ssp. elongata (Hoffm.) Bonnier 2 360 0.000 original measurement

Armeria maritima ssp. elongata (Hoffm.) Bonnier 2 1440 0.000 original measurement

Armeria maritima ssp. elongata (Hoffm.) Bonnier 3 0 1.000 original measurement

Armeria maritima ssp. elongata (Hoffm.) Bonnier 3 1 0.377 original measurement

Armeria maritima ssp. elongata (Hoffm.) Bonnier 3 5 0.283 original measurement

Armeria maritima ssp. elongata (Hoffm.) Bonnier 3 10 0.283 original measurement

Armeria maritima ssp. elongata (Hoffm.) Bonnier 3 30 0.226 original measurement

Armeria maritima ssp. elongata (Hoffm.) Bonnier 3 60 0.208 original measurement

Armeria maritima ssp. elongata (Hoffm.) Bonnier 3 120 0.075 original measurement

Armeria maritima ssp. elongata (Hoffm.) Bonnier 3 180 0.057 original measurement

Armeria maritima ssp. elongata (Hoffm.) Bonnier 3 360 0.038 original measurement

Armeria maritima ssp. elongata (Hoffm.) Bonnier 3 1440 0.000 original measurement

Armeria maritima ssp. elongata (Hoffm.) Bonnier 4 0 1.000 original measurement

Armeria maritima ssp. elongata (Hoffm.) Bonnier 4 1 0.269 original measurement

Armeria maritima ssp. elongata (Hoffm.) Bonnier 4 5 0.231 original measurement

Armeria maritima ssp. elongata (Hoffm.) Bonnier 4 10 0.218 original measurement

Armeria maritima ssp. elongata (Hoffm.) Bonnier 4 30 0.179 original measurement

Armeria maritima ssp. elongata (Hoffm.) Bonnier 4 60 0.167 original measurement

Armeria maritima ssp. elongata (Hoffm.) Bonnier 4 120 0.064 original measurement

Armeria maritima ssp. elongata (Hoffm.) Bonnier 4 180 0.038 original measurement

Armeria maritima ssp. elongata (Hoffm.) Bonnier 4 360 0.000 original measurement

Armeria maritima ssp. elongata (Hoffm.) Bonnier 4 1440 0.000 original measurement

Aster bellidiastrum (L.) Scop. 1 0 1.000 Lutz (2004)

Aster bellidiastrum (L.) Scop. 1 1 0.148 Lutz (2004)

Aster bellidiastrum (L.) Scop. 1 5 0.123 Lutz (2004)

Aster bellidiastrum (L.) Scop. 1 10 0.123 Lutz (2004)

Aster bellidiastrum (L.) Scop. 1 30 0.123 Lutz (2004)

Aster bellidiastrum (L.) Scop. 1 60 0.111 Lutz (2004)

Aster bellidiastrum (L.) Scop. 1 120 0.086 Lutz (2004)

Aster bellidiastrum (L.) Scop. 1 180 0.012 Lutz (2004)

Aster bellidiastrum (L.) Scop. 1 360 0.000 Lutz (2004)

Aster bellidiastrum (L.) Scop. 1 1440 0.000 Lutz (2004)

Aster bellidiastrum (L.) Scop. 2 0 1.000 Lutz (2004)

Aster bellidiastrum (L.) Scop. 2 1 0.422 Lutz (2004)

Aster bellidiastrum (L.) Scop. 2 5 0.378 Lutz (2004)

Aster bellidiastrum (L.) Scop. 2 10 0.378 Lutz (2004)

Aster bellidiastrum (L.) Scop. 2 30 0.367 Lutz (2004)

Aster bellidiastrum (L.) Scop. 2 60 0.356 Lutz (2004)

Aster bellidiastrum (L.) Scop. 2 120 0.233 Lutz (2004)

Aster bellidiastrum (L.) Scop. 2 180 0.133 Lutz (2004)

Aster bellidiastrum (L.) Scop. 2 360 0.000 Lutz (2004)

Aster bellidiastrum (L.) Scop. 2 1440 0.000 Lutz (2004)

Aster bellidiastrum (L.) Scop. 3 0 1.000 Lutz (2004)

Aster bellidiastrum (L.) Scop. 3 1 0.170 Lutz (2004)

Aster bellidiastrum (L.) Scop. 3 5 0.080 Lutz (2004)

Aster bellidiastrum (L.) Scop. 3 10 0.057 Lutz (2004)

Aster bellidiastrum (L.) Scop. 3 30 0.045 Lutz (2004)

Aster bellidiastrum (L.) Scop. 3 60 0.045 Lutz (2004)

Aster bellidiastrum (L.) Scop. 3 120 0.023 Lutz (2004)

Aster bellidiastrum (L.) Scop. 3 180 0.011 Lutz (2004)

Aster bellidiastrum (L.) Scop. 3 360 0.011 Lutz (2004)

Aster bellidiastrum (L.) Scop. 3 1440 0.000 Lutz (2004)

Aster bellidiastrum (L.) Scop. 4 0 1.000 Lutz (2004)

Aster bellidiastrum (L.) Scop. 4 1 0.061 Lutz (2004)

Aster bellidiastrum (L.) Scop. 4 5 0.037 Lutz (2004)

Aster bellidiastrum (L.) Scop. 4 10 0.037 Lutz (2004)

Aster bellidiastrum (L.) Scop. 4 30 0.024 Lutz (2004)

Aster bellidiastrum (L.) Scop. 4 60 0.024 Lutz (2004)

Aster bellidiastrum (L.) Scop. 4 120 0.012 Lutz (2004)

Aster bellidiastrum (L.) Scop. 4 180 0.000 Lutz (2004)

Aster bellidiastrum (L.) Scop. 4 360 0.000 Lutz (2004)

Aster bellidiastrum (L.) Scop. 4 1440 0.000 Lutz (2004)

Aster bellidiastrum (L.) Scop. 5 0 1.000 Lutz (2004)

Aster bellidiastrum (L.) Scop. 5 1 0.141 Lutz (2004)

Aster bellidiastrum (L.) Scop. 5 5 0.125 Lutz (2004)

Aster bellidiastrum (L.) Scop. 5 10 0.125 Lutz (2004)

Aster bellidiastrum (L.) Scop. 5 30 0.109 Lutz (2004)

Aster bellidiastrum (L.) Scop. 5 60 0.109 Lutz (2004)

Aster bellidiastrum (L.) Scop. 5 120 0.000 Lutz (2004)

Aster bellidiastrum (L.) Scop. 5 180 0.000 Lutz (2004)

Aster bellidiastrum (L.) Scop. 5 360 0.000 Lutz (2004)

Aster bellidiastrum (L.) Scop. 5 1440 0.000 Lutz (2004)

Bartsia alpina L. 1 0 1.000 Lutz (2004)

Bartsia alpina L. 1 1 0.247 Lutz (2004)

Bartsia alpina L. 1 5 0.247 Lutz (2004)

Bartsia alpina L. 1 10 0.233 Lutz (2004)

Bartsia alpina L. 1 30 0.219 Lutz (2004)

Bartsia alpina L. 1 60 0.192 Lutz (2004)

Bartsia alpina L. 1 120 0.096 Lutz (2004)

Bartsia alpina L. 1 180 0.096 Lutz (2004)

Bartsia alpina L. 1 360 0.014 Lutz (2004)

Bartsia alpina L. 1 1440 0.000 Lutz (2004)

Bartsia alpina L. 2 0 1.000 Lutz (2004)

Bartsia alpina L. 2 1 0.217 Lutz (2004)

Bartsia alpina L. 2 5 0.181 Lutz (2004)

Bartsia alpina L. 2 10 0.181 Lutz (2004)

Bartsia alpina L. 2 30 0.181 Lutz (2004)

Bartsia alpina L. 2 60 0.169 Lutz (2004)

Bartsia alpina L. 2 120 0.108 Lutz (2004)

Bartsia alpina L. 2 180 0.072 Lutz (2004)

Bartsia alpina L. 2 360 0.012 Lutz (2004)

Bartsia alpina L. 2 1440 0.000 Lutz (2004)

Bartsia alpina L. 3 0 1.000 Lutz (2004)

Bartsia alpina L. 3 1 0.152 Lutz (2004)

Bartsia alpina L. 3 5 0.120 Lutz (2004)

Bartsia alpina L. 3 10 0.087 Lutz (2004)

Bartsia alpina L. 3 30 0.065 Lutz (2004)

Bartsia alpina L. 3 60 0.065 Lutz (2004)

Bartsia alpina L. 3 120 0.043 Lutz (2004)

Bartsia alpina L. 3 180 0.000 Lutz (2004)

Bartsia alpina L. 3 360 0.000 Lutz (2004)

Bartsia alpina L. 3 1440 0.000 Lutz (2004)

Bartsia alpina L. 4 0 1.000 Lutz (2004)

Bartsia alpina L. 4 1 0.063 Lutz (2004)

Bartsia alpina L. 4 5 0.013 Lutz (2004)

Bartsia alpina L. 4 10 0.013 Lutz (2004)

Bartsia alpina L. 4 30 0.013 Lutz (2004)

Bartsia alpina L. 4 60 0.013 Lutz (2004)

Bartsia alpina L. 4 120 0.013 Lutz (2004)

Bartsia alpina L. 4 180 0.000 Lutz (2004)

Bartsia alpina L. 4 360 0.000 Lutz (2004)

Bartsia alpina L. 4 1440 0.000 Lutz (2004)

Bartsia alpina L. 5 0 1.000 Lutz (2004)

Bartsia alpina L. 5 1 0.097 Lutz (2004)

Bartsia alpina L. 5 5 0.048 Lutz (2004)

Bartsia alpina L. 5 10 0.032 Lutz (2004)

Bartsia alpina L. 5 30 0.032 Lutz (2004)

Bartsia alpina L. 5 60 0.016 Lutz (2004)

Bartsia alpina L. 5 120 0.016 Lutz (2004)

Bartsia alpina L. 5 180 0.000 Lutz (2004)

Bartsia alpina L. 5 360 0.000 Lutz (2004)

Bartsia alpina L. 5 1440 0.000 Lutz (2004)

Briza media L. 1 0 1.000 original measurement

Briza media L. 1 1 0.081 original measurement

Briza media L. 1 5 0.054 original measurement

Briza media L. 1 10 0.027 original measurement

Briza media L. 1 30 0.000 original measurement

Briza media L. 1 60 0.000 original measurement

Briza media L. 1 120 0.000 original measurement

Briza media L. 1 180 0.000 original measurement

Briza media L. 1 360 0.000 original measurement

Briza media L. 1 1440 0.000 original measurement

Briza media L. 2 0 1.000 original measurement

Briza media L. 2 1 0.061 original measurement

Briza media L. 2 5 0.000 original measurement

Briza media L. 2 10 0.000 original measurement

Briza media L. 2 30 0.000 original measurement

Briza media L. 2 60 0.000 original measurement

Briza media L. 2 120 0.000 original measurement

Briza media L. 2 180 0.000 original measurement

Briza media L. 2 360 0.000 original measurement

Briza media L. 2 1440 0.000 original measurement

Briza media L. 3 0 1.000 original measurement

Briza media L. 3 1 0.109 original measurement

Briza media L. 3 5 0.087 original measurement

Briza media L. 3 10 0.043 original measurement

Briza media L. 3 30 0.043 original measurement

Briza media L. 3 60 0.022 original measurement

Briza media L. 3 120 0.000 original measurement

Briza media L. 3 180 0.000 original measurement

Briza media L. 3 360 0.000 original measurement

Briza media L. 3 1440 0.000 original measurement

Briza media L. 4 0 1.000 original measurement

Briza media L. 4 1 0.081 original measurement

Briza media L. 4 5 0.032 original measurement

Briza media L. 4 10 0.016 original measurement

Briza media L. 4 30 0.000 original measurement

Briza media L. 4 60 0.000 original measurement

Briza media L. 4 120 0.000 original measurement

Briza media L. 4 180 0.000 original measurement

Briza media L. 4 360 0.000 original measurement

Briza media L. 4 1440 0.000 original measurement

Bromus catharticus M. Vahl 1 0 1.000 Pirzer (2007)

Bromus catharticus M. Vahl 1 1 0.071 Pirzer (2007)

Bromus catharticus M. Vahl 1 5 0.036 Pirzer (2007)

Bromus catharticus M. Vahl 1 10 0.036 Pirzer (2007)

Bromus catharticus M. Vahl 1 30 0.036 Pirzer (2007)

Bromus catharticus M. Vahl 1 60 0.036 Pirzer (2007)

Bromus catharticus M. Vahl 1 120 0.036 Pirzer (2007)

Bromus catharticus M. Vahl 1 180 0.000 Pirzer (2007)

Bromus catharticus M. Vahl 1 360 0.000 Pirzer (2007)

Bromus catharticus M. Vahl 1 1440 0.000 Pirzer (2007)

Bromus catharticus M. Vahl 2 0 1.000 Pirzer (2007)

Bromus catharticus M. Vahl 2 1 0.065 Pirzer (2007)

Bromus catharticus M. Vahl 2 5 0.043 Pirzer (2007)

Bromus catharticus M. Vahl 2 10 0.022 Pirzer (2007)

Bromus catharticus M. Vahl 2 30 0.022 Pirzer (2007)

Bromus catharticus M. Vahl 2 60 0.022 Pirzer (2007)

Bromus catharticus M. Vahl 2 120 0.022 Pirzer (2007)

Bromus catharticus M. Vahl 2 180 0.022 Pirzer (2007)

Bromus catharticus M. Vahl 2 360 0.000 Pirzer (2007)

Bromus catharticus M. Vahl 2 1440 0.000 Pirzer (2007)

Bromus catharticus M. Vahl 3 0 1.000 Pirzer (2007)

Bromus catharticus M. Vahl 3 1 0.050 Pirzer (2007)

Bromus catharticus M. Vahl 3 5 0.050 Pirzer (2007)

Bromus catharticus M. Vahl 3 10 0.050 Pirzer (2007)

Bromus catharticus M. Vahl 3 30 0.050 Pirzer (2007)

Bromus catharticus M. Vahl 3 60 0.050 Pirzer (2007)

Bromus catharticus M. Vahl 3 120 0.000 Pirzer (2007)

Bromus catharticus M. Vahl 3 180 0.000 Pirzer (2007)

Bromus catharticus M. Vahl 3 360 0.000 Pirzer (2007)

Bromus catharticus M. Vahl 3 1440 0.000 Pirzer (2007)

Bromus catharticus M. Vahl 4 0 1.000 Pirzer (2007)

Bromus catharticus M. Vahl 4 1 0.063 Pirzer (2007)

Bromus catharticus M. Vahl 4 5 0.063 Pirzer (2007)

Bromus catharticus M. Vahl 4 10 0.063 Pirzer (2007)

Bromus catharticus M. Vahl 4 30 0.063 Pirzer (2007)

Bromus catharticus M. Vahl 4 60 0.063 Pirzer (2007)

Bromus catharticus M. Vahl 4 120 0.000 Pirzer (2007)

Bromus catharticus M. Vahl 4 180 0.000 Pirzer (2007)

Bromus catharticus M. Vahl 4 360 0.000 Pirzer (2007)

Bromus catharticus M. Vahl 4 1440 0.000 Pirzer (2007)

Bromus catharticus M. Vahl 5 0 1.000 Pirzer (2007)

Bromus catharticus M. Vahl 5 1 0.200 Pirzer (2007)

Bromus catharticus M. Vahl 5 5 0.200 Pirzer (2007)

Bromus catharticus M. Vahl 5 10 0.200 Pirzer (2007)

Bromus catharticus M. Vahl 5 30 0.150 Pirzer (2007)

Bromus catharticus M. Vahl 5 60 0.150 Pirzer (2007)

Bromus catharticus M. Vahl 5 120 0.000 Pirzer (2007)

Bromus catharticus M. Vahl 5 180 0.000 Pirzer (2007)

Bromus catharticus M. Vahl 5 360 0.000 Pirzer (2007)

Bromus catharticus M. Vahl 5 1440 0.000 Pirzer (2007)

Bromus diandrus Roth 1 0 1.000 Pirzer (2007)

Bromus diandrus Roth 1 1 0.615 Pirzer (2007)

Bromus diandrus Roth 1 5 0.308 Pirzer (2007)

Bromus diandrus Roth 1 10 0.308 Pirzer (2007)

Bromus diandrus Roth 1 30 0.308 Pirzer (2007)

Bromus diandrus Roth 1 60 0.231 Pirzer (2007)

Bromus diandrus Roth 1 120 0.231 Pirzer (2007)

Bromus diandrus Roth 1 180 0.077 Pirzer (2007)

Bromus diandrus Roth 1 360 0.077 Pirzer (2007)

Bromus diandrus Roth 1 1440 0.000 Pirzer (2007)

Bromus diandrus Roth 2 0 1.000 Pirzer (2007)

Bromus diandrus Roth 2 1 0.375 Pirzer (2007)

Bromus diandrus Roth 2 5 0.375 Pirzer (2007)

Bromus diandrus Roth 2 10 0.333 Pirzer (2007)

Bromus diandrus Roth 2 30 0.333 Pirzer (2007)

Bromus diandrus Roth 2 60 0.292 Pirzer (2007)

Bromus diandrus Roth 2 120 0.125 Pirzer (2007)

Bromus diandrus Roth 2 180 0.083 Pirzer (2007)

Bromus diandrus Roth 2 360 0.042 Pirzer (2007)

Bromus diandrus Roth 2 1440 0.000 Pirzer (2007)

Bromus diandrus Roth 3 0 1.000 Pirzer (2007)

Bromus diandrus Roth 3 1 0.429 Pirzer (2007)

Bromus diandrus Roth 3 5 0.333 Pirzer (2007)

Bromus diandrus Roth 3 10 0.333 Pirzer (2007)

Bromus diandrus Roth 3 30 0.143 Pirzer (2007)

Bromus diandrus Roth 3 60 0.143 Pirzer (2007)

Bromus diandrus Roth 3 120 0.143 Pirzer (2007)

Bromus diandrus Roth 3 180 0.095 Pirzer (2007)

Bromus diandrus Roth 3 360 0.000 Pirzer (2007)

Bromus diandrus Roth 3 1440 0.000 Pirzer (2007)

Bromus diandrus Roth 4 0 1.000 Pirzer (2007)

Bromus diandrus Roth 4 1 0.333 Pirzer (2007)

Bromus diandrus Roth 4 5 0.250 Pirzer (2007)

Bromus diandrus Roth 4 10 0.250 Pirzer (2007)

Bromus diandrus Roth 4 30 0.167 Pirzer (2007)

Bromus diandrus Roth 4 60 0.167 Pirzer (2007)

Bromus diandrus Roth 4 120 0.000 Pirzer (2007)

Bromus diandrus Roth 4 180 0.000 Pirzer (2007)

Bromus diandrus Roth 4 360 0.000 Pirzer (2007)

Bromus diandrus Roth 4 1440 0.000 Pirzer (2007)

Bromus diandrus Roth 5 0 1.000 Pirzer (2007)

Bromus diandrus Roth 5 1 0.706 Pirzer (2007)

Bromus diandrus Roth 5 5 0.618 Pirzer (2007)

Bromus diandrus Roth 5 10 0.588 Pirzer (2007)

Bromus diandrus Roth 5 30 0.588 Pirzer (2007)

Bromus diandrus Roth 5 60 0.559 Pirzer (2007)

Bromus diandrus Roth 5 120 0.118 Pirzer (2007)

Bromus diandrus Roth 5 180 0.059 Pirzer (2007)

Bromus diandrus Roth 5 360 0.000 Pirzer (2007)

Bromus diandrus Roth 5 1440 0.000 Pirzer (2007)

Bromus intermedius Guss. 1 0 1.000 Pirzer (2007)

Bromus intermedius Guss. 1 1 0.281 Pirzer (2007)

Bromus intermedius Guss. 1 5 0.219 Pirzer (2007)

Bromus intermedius Guss. 1 10 0.219 Pirzer (2007)

Bromus intermedius Guss. 1 30 0.219 Pirzer (2007)

Bromus intermedius Guss. 1 60 0.188 Pirzer (2007)

Bromus intermedius Guss. 1 120 0.000 Pirzer (2007)

Bromus intermedius Guss. 1 180 0.000 Pirzer (2007)

Bromus intermedius Guss. 1 360 0.000 Pirzer (2007)

Bromus intermedius Guss. 1 1440 0.000 Pirzer (2007)

Bromus intermedius Guss. 2 0 1.000 Pirzer (2007)

Bromus intermedius Guss. 2 1 0.149 Pirzer (2007)

Bromus intermedius Guss. 2 5 0.106 Pirzer (2007)

Bromus intermedius Guss. 2 10 0.106 Pirzer (2007)

Bromus intermedius Guss. 2 30 0.064 Pirzer (2007)

Bromus intermedius Guss. 2 60 0.064 Pirzer (2007)

Bromus intermedius Guss. 2 120 0.000 Pirzer (2007)

Bromus intermedius Guss. 2 180 0.000 Pirzer (2007)

Bromus intermedius Guss. 2 360 0.000 Pirzer (2007)

Bromus intermedius Guss. 2 1440 0.000 Pirzer (2007)

Bromus intermedius Guss. 3 0 1.000 Pirzer (2007)

Bromus intermedius Guss. 3 1 0.209 Pirzer (2007)

Bromus intermedius Guss. 3 5 0.186 Pirzer (2007)

Bromus intermedius Guss. 3 10 0.186 Pirzer (2007)

Bromus intermedius Guss. 3 30 0.163 Pirzer (2007)

Bromus intermedius Guss. 3 60 0.163 Pirzer (2007)

Bromus intermedius Guss. 3 120 0.070 Pirzer (2007)

Bromus intermedius Guss. 3 180 0.023 Pirzer (2007)

Bromus intermedius Guss. 3 360 0.000 Pirzer (2007)

Bromus intermedius Guss. 3 1440 0.000 Pirzer (2007)

Bromus intermedius Guss. 4 0 1.000 Pirzer (2007)

Bromus intermedius Guss. 4 1 0.171 Pirzer (2007)

Bromus intermedius Guss. 4 5 0.122 Pirzer (2007)

Bromus intermedius Guss. 4 10 0.098 Pirzer (2007)

Bromus intermedius Guss. 4 30 0.073 Pirzer (2007)

Bromus intermedius Guss. 4 60 0.073 Pirzer (2007)

Bromus intermedius Guss. 4 120 0.073 Pirzer (2007)

Bromus intermedius Guss. 4 180 0.024 Pirzer (2007)

Bromus intermedius Guss. 4 360 0.000 Pirzer (2007)

Bromus intermedius Guss. 4 1440 0.000 Pirzer (2007)

Bromus intermedius Guss. 5 0 1.000 Pirzer (2007)

Bromus intermedius Guss. 5 1 0.410 Pirzer (2007)

Bromus intermedius Guss. 5 5 0.385 Pirzer (2007)

Bromus intermedius Guss. 5 10 0.385 Pirzer (2007)

Bromus intermedius Guss. 5 30 0.308 Pirzer (2007)

Bromus intermedius Guss. 5 60 0.256 Pirzer (2007)

Bromus intermedius Guss. 5 120 0.154 Pirzer (2007)

Bromus intermedius Guss. 5 180 0.026 Pirzer (2007)

Bromus intermedius Guss. 5 360 0.000 Pirzer (2007)

Bromus intermedius Guss. 5 1440 0.000 Pirzer (2007)

Calluna vulgaris (L.) Hull 1 0 1.000 original measurement

Calluna vulgaris (L.) Hull 1 1 0.384 original measurement

Calluna vulgaris (L.) Hull 1 5 0.302 original measurement

Calluna vulgaris (L.) Hull 1 10 0.267 original measurement

Calluna vulgaris (L.) Hull 1 30 0.209 original measurement

Calluna vulgaris (L.) Hull 1 60 0.186 original measurement

Calluna vulgaris (L.) Hull 1 120 0.070 original measurement

Calluna vulgaris (L.) Hull 1 180 0.000 original measurement

Calluna vulgaris (L.) Hull 2 0 1.000 original measurement

Calluna vulgaris (L.) Hull 2 1 0.341 original measurement

Calluna vulgaris (L.) Hull 2 5 0.271 original measurement

Calluna vulgaris (L.) Hull 2 10 0.235 original measurement

Calluna vulgaris (L.) Hull 2 30 0.165 original measurement

Calluna vulgaris (L.) Hull 2 60 0.141 original measurement

Calluna vulgaris (L.) Hull 2 120 0.082 original measurement

Calluna vulgaris (L.) Hull 2 180 0.059 original measurement

Calluna vulgaris (L.) Hull 2 360 0.012 original measurement

Calluna vulgaris (L.) Hull 2 1440 0.000 original measurement

Calluna vulgaris (L.) Hull 3 0 1.000 original measurement

Calluna vulgaris (L.) Hull 3 1 0.343 original measurement

Calluna vulgaris (L.) Hull 3 5 0.257 original measurement

Calluna vulgaris (L.) Hull 3 10 0.214 original measurement

Calluna vulgaris (L.) Hull 3 30 0.157 original measurement

Calluna vulgaris (L.) Hull 3 60 0.143 original measurement

Calluna vulgaris (L.) Hull 3 120 0.029 original measurement

Calluna vulgaris (L.) Hull 3 180 0.029 original measurement

Calluna vulgaris (L.) Hull 3 360 0.000 original measurement

Calluna vulgaris (L.) Hull 3 1440 0.000 original measurement

Calluna vulgaris (L.) Hull 4 0 1.000 original measurement

Calluna vulgaris (L.) Hull 4 1 0.779 original measurement

Calluna vulgaris (L.) Hull 4 5 0.649 original measurement

Calluna vulgaris (L.) Hull 4 10 0.649 original measurement

Calluna vulgaris (L.) Hull 4 30 0.610 original measurement

Calluna vulgaris (L.) Hull 4 60 0.610 original measurement

Calluna vulgaris (L.) Hull 4 120 0.429 original measurement

Calluna vulgaris (L.) Hull 4 180 0.351 original measurement

Calluna vulgaris (L.) Hull 4 360 0.013 original measurement

Calluna vulgaris (L.) Hull 4 1440 0.000 original measurement

Cerastium uniflorum Clairv. 1 0 1.000 Lutz (2004)

Cerastium uniflorum Clairv. 1 1 0.214 Lutz (2004)

Cerastium uniflorum Clairv. 1 5 0.171 Lutz (2004)

Cerastium uniflorum Clairv. 1 10 0.157 Lutz (2004)

Cerastium uniflorum Clairv. 1 30 0.129 Lutz (2004)

Cerastium uniflorum Clairv. 1 60 0.129 Lutz (2004)

Cerastium uniflorum Clairv. 1 120 0.043 Lutz (2004)

Cerastium uniflorum Clairv. 1 180 0.029 Lutz (2004)

Cerastium uniflorum Clairv. 1 360 0.014 Lutz (2004)

Cerastium uniflorum Clairv. 1 1440 0.000 Lutz (2004)

Cerastium uniflorum Clairv. 2 0 1.000 Lutz (2004)

Cerastium uniflorum Clairv. 2 1 0.284 Lutz (2004)

Cerastium uniflorum Clairv. 2 5 0.227 Lutz (2004)

Cerastium uniflorum Clairv. 2 10 0.193 Lutz (2004)

Cerastium uniflorum Clairv. 2 30 0.182 Lutz (2004)

Cerastium uniflorum Clairv. 2 60 0.159 Lutz (2004)

Cerastium uniflorum Clairv. 2 120 0.080 Lutz (2004)

Cerastium uniflorum Clairv. 2 180 0.080 Lutz (2004)

Cerastium uniflorum Clairv. 2 360 0.011 Lutz (2004)

Cerastium uniflorum Clairv. 2 1440 0.000 Lutz (2004)

Cerastium uniflorum Clairv. 3 0 1.000 Lutz (2004)

Cerastium uniflorum Clairv. 3 1 0.152 Lutz (2004)

Cerastium uniflorum Clairv. 3 5 0.109 Lutz (2004)

Cerastium uniflorum Clairv. 3 10 0.087 Lutz (2004)

Cerastium uniflorum Clairv. 3 30 0.076 Lutz (2004)

Cerastium uniflorum Clairv. 3 60 0.076 Lutz (2004)

Cerastium uniflorum Clairv. 3 120 0.011 Lutz (2004)

Cerastium uniflorum Clairv. 3 180 0.000 Lutz (2004)

Cerastium uniflorum Clairv. 3 360 0.000 Lutz (2004)

Cerastium uniflorum Clairv. 3 1440 0.000 Lutz (2004)

Cerastium uniflorum Clairv. 4 0 1.000 Lutz (2004)

Cerastium uniflorum Clairv. 4 1 0.109 Lutz (2004)

Cerastium uniflorum Clairv. 4 5 0.063 Lutz (2004)

Cerastium uniflorum Clairv. 4 10 0.063 Lutz (2004)

Cerastium uniflorum Clairv. 4 30 0.047 Lutz (2004)

Cerastium uniflorum Clairv. 4 60 0.047 Lutz (2004)

Cerastium uniflorum Clairv. 4 120 0.031 Lutz (2004)

Cerastium uniflorum Clairv. 4 180 0.016 Lutz (2004)

Cerastium uniflorum Clairv. 4 360 0.000 Lutz (2004)

Cerastium uniflorum Clairv. 4 1440 0.000 Lutz (2004)

Cerastium uniflorum Clairv. 5 0 1.000 Lutz (2004)

Cerastium uniflorum Clairv. 5 1 0.179 Lutz (2004)

Cerastium uniflorum Clairv. 5 5 0.149 Lutz (2004)

Cerastium uniflorum Clairv. 5 10 0.119 Lutz (2004)

Cerastium uniflorum Clairv. 5 30 0.104 Lutz (2004)

Cerastium uniflorum Clairv. 5 60 0.090 Lutz (2004)

Cerastium uniflorum Clairv. 5 120 0.015 Lutz (2004)

Cerastium uniflorum Clairv. 5 180 0.000 Lutz (2004)

Cerastium uniflorum Clairv. 5 360 0.000 Lutz (2004)

Cerastium uniflorum Clairv. 5 1440 0.000 Lutz (2004)

Clinopodium vulgare L. 1 0 1.000 original measurement

Clinopodium vulgare L. 1 1 0.255 original measurement

Clinopodium vulgare L. 1 5 0.176 original measurement

Clinopodium vulgare L. 1 10 0.157 original measurement

Clinopodium vulgare L. 1 30 0.098 original measurement

Clinopodium vulgare L. 1 60 0.098 original measurement

Clinopodium vulgare L. 1 120 0.039 original measurement

Clinopodium vulgare L. 1 180 0.000 original measurement

Clinopodium vulgare L. 2 0 1.000 original measurement

Clinopodium vulgare L. 2 1 0.140 original measurement

Clinopodium vulgare L. 2 5 0.140 original measurement

Clinopodium vulgare L. 2 10 0.070 original measurement

Clinopodium vulgare L. 2 30 0.018 original measurement

Clinopodium vulgare L. 2 60 0.018 original measurement

Clinopodium vulgare L. 2 120 0.018 original measurement

Clinopodium vulgare L. 2 180 0.000 original measurement

Clinopodium vulgare L. 2 360 0.000 original measurement

Clinopodium vulgare L. 2 1440 0.000 original measurement

Clinopodium vulgare L. 3 0 1.000 original measurement

Clinopodium vulgare L. 3 1 0.338 original measurement

Clinopodium vulgare L. 3 5 0.312 original measurement

Clinopodium vulgare L. 3 10 0.286 original measurement

Clinopodium vulgare L. 3 30 0.273 original measurement

Clinopodium vulgare L. 3 60 0.273 original measurement

Clinopodium vulgare L. 3 120 0.208 original measurement

Clinopodium vulgare L. 3 180 0.195 original measurement

Clinopodium vulgare L. 3 360 0.104 original measurement

Clinopodium vulgare L. 3 1440 0.000 original measurement

Clinopodium vulgare L. 4 0 1.000 original measurement

Clinopodium vulgare L. 4 1 0.257 original measurement

Clinopodium vulgare L. 4 5 0.186 original measurement

Clinopodium vulgare L. 4 10 0.186 original measurement

Clinopodium vulgare L. 4 30 0.171 original measurement

Clinopodium vulgare L. 4 60 0.171 original measurement

Clinopodium vulgare L. 4 120 0.100 original measurement

Clinopodium vulgare L. 4 180 0.086 original measurement

Clinopodium vulgare L. 4 360 0.000 original measurement

Clinopodium vulgare L. 4 1440 0.000 original measurement

Cynodon dactylon (L.) Pers. 1 0 1.000 Pirzer (2007)

Cynodon dactylon (L.) Pers. 1 1 0.091 Pirzer (2007)

Cynodon dactylon (L.) Pers. 1 5 0.045 Pirzer (2007)

Cynodon dactylon (L.) Pers. 1 10 0.000 Pirzer (2007)

Cynodon dactylon (L.) Pers. 1 30 0.000 Pirzer (2007)

Cynodon dactylon (L.) Pers. 1 60 0.000 Pirzer (2007)

Cynodon dactylon (L.) Pers. 1 120 0.000 Pirzer (2007)

Cynodon dactylon (L.) Pers. 1 180 0.000 Pirzer (2007)

Cynodon dactylon (L.) Pers. 1 360 0.000 Pirzer (2007)

Cynodon dactylon (L.) Pers. 1 1440 0.000 Pirzer (2007)

Cynodon dactylon (L.) Pers. 2 0 1.000 Pirzer (2007)

Cynodon dactylon (L.) Pers. 2 1 0.780 Pirzer (2007)

Cynodon dactylon (L.) Pers. 2 5 0.780 Pirzer (2007)

Cynodon dactylon (L.) Pers. 2 10 0.780 Pirzer (2007)

Cynodon dactylon (L.) Pers. 2 30 0.780 Pirzer (2007)

Cynodon dactylon (L.) Pers. 2 60 0.780 Pirzer (2007)

Cynodon dactylon (L.) Pers. 2 120 0.415 Pirzer (2007)

Cynodon dactylon (L.) Pers. 2 180 0.024 Pirzer (2007)

Cynodon dactylon (L.) Pers. 2 360 0.000 Pirzer (2007)

Cynodon dactylon (L.) Pers. 2 1440 0.000 Pirzer (2007)

Cynodon dactylon (L.) Pers. 3 0 1.000 Pirzer (2007)

Cynodon dactylon (L.) Pers. 3 1 0.950 Pirzer (2007)

Cynodon dactylon (L.) Pers. 3 5 0.850 Pirzer (2007)

Cynodon dactylon (L.) Pers. 3 10 0.850 Pirzer (2007)

Cynodon dactylon (L.) Pers. 3 30 0.850 Pirzer (2007)

Cynodon dactylon (L.) Pers. 3 60 0.850 Pirzer (2007)

Cynodon dactylon (L.) Pers. 3 120 0.650 Pirzer (2007)

Cynodon dactylon (L.) Pers. 3 180 0.600 Pirzer (2007)

Cynodon dactylon (L.) Pers. 3 360 0.150 Pirzer (2007)

Cynodon dactylon (L.) Pers. 3 1440 0.000 Pirzer (2007)

Cynodon dactylon (L.) Pers. 4 0 1.000 Pirzer (2007)

Cynodon dactylon (L.) Pers. 4 1 0.606 Pirzer (2007)

Cynodon dactylon (L.) Pers. 4 5 0.576 Pirzer (2007)

Cynodon dactylon (L.) Pers. 4 10 0.576 Pirzer (2007)

Cynodon dactylon (L.) Pers. 4 30 0.455 Pirzer (2007)

Cynodon dactylon (L.) Pers. 4 60 0.394 Pirzer (2007)

Cynodon dactylon (L.) Pers. 4 120 0.182 Pirzer (2007)

Cynodon dactylon (L.) Pers. 4 180 0.061 Pirzer (2007)

Cynodon dactylon (L.) Pers. 4 360 0.030 Pirzer (2007)

Cynodon dactylon (L.) Pers. 4 1440 0.000 Pirzer (2007)

Cynodon dactylon (L.) Pers. 5 0 1.000 Pirzer (2007)

Cynodon dactylon (L.) Pers. 5 1 0.618 Pirzer (2007)

Cynodon dactylon (L.) Pers. 5 5 0.559 Pirzer (2007)

Cynodon dactylon (L.) Pers. 5 10 0.412 Pirzer (2007)

Cynodon dactylon (L.) Pers. 5 30 0.324 Pirzer (2007)

Cynodon dactylon (L.) Pers. 5 60 0.324 Pirzer (2007)

Cynodon dactylon (L.) Pers. 5 120 0.176 Pirzer (2007)

Cynodon dactylon (L.) Pers. 5 180 0.118 Pirzer (2007)

Cynodon dactylon (L.) Pers. 5 360 0.000 Pirzer (2007)

Cynodon dactylon (L.) Pers. 5 1440 0.000 Pirzer (2007)

Danthonia decumbens (L.) DC. 1 0 1.000 Pirzer (2007)

Danthonia decumbens (L.) DC. 1 1 0.385 Pirzer (2007)

Danthonia decumbens (L.) DC. 1 5 0.308 Pirzer (2007)

Danthonia decumbens (L.) DC. 1 10 0.231 Pirzer (2007)

Danthonia decumbens (L.) DC. 1 30 0.231 Pirzer (2007)

Danthonia decumbens (L.) DC. 1 60 0.231 Pirzer (2007)

Danthonia decumbens (L.) DC. 1 120 0.077 Pirzer (2007)

Danthonia decumbens (L.) DC. 1 180 0.000 Pirzer (2007)

Danthonia decumbens (L.) DC. 1 360 0.000 Pirzer (2007)

Danthonia decumbens (L.) DC. 1 1440 0.000 Pirzer (2007)

Danthonia decumbens (L.) DC. 2 0 1.000 Pirzer (2007)

Danthonia decumbens (L.) DC. 2 1 0.625 Pirzer (2007)

Danthonia decumbens (L.) DC. 2 5 0.375 Pirzer (2007)

Danthonia decumbens (L.) DC. 2 10 0.375 Pirzer (2007)

Danthonia decumbens (L.) DC. 2 30 0.375 Pirzer (2007)

Danthonia decumbens (L.) DC. 2 60 0.375 Pirzer (2007)

Danthonia decumbens (L.) DC. 2 120 0.125 Pirzer (2007)

Danthonia decumbens (L.) DC. 2 180 0.063 Pirzer (2007)

Danthonia decumbens (L.) DC. 2 360 0.000 Pirzer (2007)

Danthonia decumbens (L.) DC. 2 1440 0.000 Pirzer (2007)

Danthonia decumbens (L.) DC. 3 0 1.000 Pirzer (2007)

Danthonia decumbens (L.) DC. 3 1 0.263 Pirzer (2007)

Danthonia decumbens (L.) DC. 3 5 0.211 Pirzer (2007)

Danthonia decumbens (L.) DC. 3 10 0.211 Pirzer (2007)

Danthonia decumbens (L.) DC. 3 30 0.158 Pirzer (2007)

Danthonia decumbens (L.) DC. 3 60 0.105 Pirzer (2007)

Danthonia decumbens (L.) DC. 3 120 0.053 Pirzer (2007)

Danthonia decumbens (L.) DC. 3 180 0.000 Pirzer (2007)

Danthonia decumbens (L.) DC. 3 360 0.000 Pirzer (2007)

Danthonia decumbens (L.) DC. 3 1440 0.000 Pirzer (2007)

Danthonia decumbens (L.) DC. 4 0 1.000 Pirzer (2007)

Danthonia decumbens (L.) DC. 4 1 0.000 Pirzer (2007)

Danthonia decumbens (L.) DC. 4 5 0.000 Pirzer (2007)

Danthonia decumbens (L.) DC. 4 10 0.000 Pirzer (2007)

Danthonia decumbens (L.) DC. 4 30 0.000 Pirzer (2007)

Danthonia decumbens (L.) DC. 4 60 0.000 Pirzer (2007)

Danthonia decumbens (L.) DC. 4 120 0.000 Pirzer (2007)

Danthonia decumbens (L.) DC. 4 180 0.000 Pirzer (2007)

Danthonia decumbens (L.) DC. 4 360 0.000 Pirzer (2007)

Danthonia decumbens (L.) DC. 4 1440 0.000 Pirzer (2007)

Danthonia decumbens (L.) DC. 5 0 1.000 Pirzer (2007)

Danthonia decumbens (L.) DC. 5 1 0.000 Pirzer (2007)

Danthonia decumbens (L.) DC. 5 5 0.000 Pirzer (2007)

Danthonia decumbens (L.) DC. 5 10 0.000 Pirzer (2007)

Danthonia decumbens (L.) DC. 5 30 0.000 Pirzer (2007)

Danthonia decumbens (L.) DC. 5 60 0.000 Pirzer (2007)

Danthonia decumbens (L.) DC. 5 120 0.000 Pirzer (2007)

Danthonia decumbens (L.) DC. 5 180 0.000 Pirzer (2007)

Danthonia decumbens (L.) DC. 5 360 0.000 Pirzer (2007)

Danthonia decumbens (L.) DC. 5 1440 0.000 Pirzer (2007)

Doronicum clusii agg. 1 0 1.000 Lutz (2004)

Doronicum clusii agg. 1 1 0.325 Lutz (2004)

Doronicum clusii agg. 1 5 0.221 Lutz (2004)

Doronicum clusii agg. 1 10 0.195 Lutz (2004)

Doronicum clusii agg. 1 30 0.169 Lutz (2004)

Doronicum clusii agg. 1 60 0.169 Lutz (2004)

Doronicum clusii agg. 1 120 0.065 Lutz (2004)

Doronicum clusii agg. 1 180 0.026 Lutz (2004)

Doronicum clusii agg. 1 360 0.000 Lutz (2004)

Doronicum clusii agg. 1 1440 0.000 Lutz (2004)

Doronicum clusii agg. 2 0 1.000 Lutz (2004)

Doronicum clusii agg. 2 1 0.309 Lutz (2004)

Doronicum clusii agg. 2 5 0.247 Lutz (2004)

Doronicum clusii agg. 2 10 0.247 Lutz (2004)

Doronicum clusii agg. 2 30 0.227 Lutz (2004)

Doronicum clusii agg. 2 60 0.227 Lutz (2004)

Doronicum clusii agg. 2 120 0.144 Lutz (2004)

Doronicum clusii agg. 2 180 0.052 Lutz (2004)

Doronicum clusii agg. 2 360 0.021 Lutz (2004)

Doronicum clusii agg. 2 1440 0.000 Lutz (2004)

Doronicum clusii agg. 3 0 1.000 Lutz (2004)

Doronicum clusii agg. 3 1 0.240 Lutz (2004)

Doronicum clusii agg. 3 5 0.150 Lutz (2004)

Doronicum clusii agg. 3 10 0.120 Lutz (2004)

Doronicum clusii agg. 3 30 0.090 Lutz (2004)

Doronicum clusii agg. 3 60 0.070 Lutz (2004)

Doronicum clusii agg. 3 120 0.000 Lutz (2004)

Doronicum clusii agg. 3 180 0.000 Lutz (2004)

Doronicum clusii agg. 3 360 0.000 Lutz (2004)

Doronicum clusii agg. 3 1440 0.000 Lutz (2004)

Doronicum clusii agg. 4 0 1.000 Lutz (2004)

Doronicum clusii agg. 4 1 0.126 Lutz (2004)

Doronicum clusii agg. 4 5 0.053 Lutz (2004)

Doronicum clusii agg. 4 10 0.032 Lutz (2004)

Doronicum clusii agg. 4 30 0.021 Lutz (2004)

Doronicum clusii agg. 4 60 0.021 Lutz (2004)

Doronicum clusii agg. 4 120 0.011 Lutz (2004)

Doronicum clusii agg. 4 180 0.000 Lutz (2004)

Doronicum clusii agg. 4 360 0.000 Lutz (2004)

Doronicum clusii agg. 4 1440 0.000 Lutz (2004)

Doronicum clusii agg. 5 0 1.000 Lutz (2004)

Doronicum clusii agg. 5 1 0.086 Lutz (2004)

Doronicum clusii agg. 5 5 0.071 Lutz (2004)

Doronicum clusii agg. 5 10 0.071 Lutz (2004)

Doronicum clusii agg. 5 30 0.071 Lutz (2004)

Doronicum clusii agg. 5 60 0.071 Lutz (2004)

Doronicum clusii agg. 5 120 0.029 Lutz (2004)

Doronicum clusii agg. 5 180 0.014 Lutz (2004)

Doronicum clusii agg. 5 360 0.000 Lutz (2004)

Doronicum clusii agg. 5 1440 0.000 Lutz (2004)

Echium vulgare L. 1 0 1.000 original measurement

Echium vulgare L. 1 1 0.000 original measurement

Echium vulgare L. 1 5 0.000 original measurement

Echium vulgare L. 1 10 0.000 original measurement

Echium vulgare L. 1 30 0.000 original measurement

Echium vulgare L. 1 60 0.000 original measurement

Echium vulgare L. 1 120 0.000 original measurement

Echium vulgare L. 1 180 0.000 original measurement

Echium vulgare L. 2 0 1.000 original measurement

Echium vulgare L. 2 1 0.056 original measurement

Echium vulgare L. 2 5 0.056 original measurement

Echium vulgare L. 2 10 0.056 original measurement

Echium vulgare L. 2 30 0.000 original measurement

Echium vulgare L. 2 60 0.000 original measurement

Echium vulgare L. 2 120 0.000 original measurement

Echium vulgare L. 2 180 0.000 original measurement

Echium vulgare L. 2 360 0.000 original measurement

Echium vulgare L. 2 1440 0.000 original measurement

Echium vulgare L. 3 0 1.000 original measurement

Echium vulgare L. 3 1 0.045 original measurement

Echium vulgare L. 3 5 0.045 original measurement

Echium vulgare L. 3 10 0.000 original measurement

Echium vulgare L. 3 30 0.000 original measurement

Echium vulgare L. 3 60 0.000 original measurement

Echium vulgare L. 3 120 0.000 original measurement

Echium vulgare L. 3 180 0.000 original measurement

Echium vulgare L. 3 360 0.000 original measurement

Echium vulgare L. 3 1440 0.000 original measurement

Echium vulgare L. 4 0 1.000 original measurement

Echium vulgare L. 4 1 0.026 original measurement

Echium vulgare L. 4 5 0.026 original measurement

Echium vulgare L. 4 10 0.026 original measurement

Echium vulgare L. 4 30 0.026 original measurement

Echium vulgare L. 4 60 0.000 original measurement

Echium vulgare L. 4 120 0.000 original measurement

Echium vulgare L. 4 180 0.000 original measurement

Echium vulgare L. 4 360 0.000 original measurement

Echium vulgare L. 4 1440 0.000 original measurement

Epilobium anagallidifolium Lam. 1 0 1.000 Lutz (2004)

Epilobium anagallidifolium Lam. 1 1 0.200 Lutz (2004)

Epilobium anagallidifolium Lam. 1 5 0.187 Lutz (2004)

Epilobium anagallidifolium Lam. 1 10 0.187 Lutz (2004)

Epilobium anagallidifolium Lam. 1 30 0.187 Lutz (2004)

Epilobium anagallidifolium Lam. 1 60 0.187 Lutz (2004)

Epilobium anagallidifolium Lam. 1 120 0.080 Lutz (2004)

Epilobium anagallidifolium Lam. 1 180 0.027 Lutz (2004)

Epilobium anagallidifolium Lam. 1 360 0.000 Lutz (2004)

Epilobium anagallidifolium Lam. 1 1440 0.000 Lutz (2004)

Epilobium anagallidifolium Lam. 2 0 1.000 Lutz (2004)

Epilobium anagallidifolium Lam. 2 1 0.230 Lutz (2004)

Epilobium anagallidifolium Lam. 2 5 0.092 Lutz (2004)

Epilobium anagallidifolium Lam. 2 10 0.092 Lutz (2004)

Epilobium anagallidifolium Lam. 2 30 0.092 Lutz (2004)

Epilobium anagallidifolium Lam. 2 60 0.092 Lutz (2004)

Epilobium anagallidifolium Lam. 2 120 0.046 Lutz (2004)

Epilobium anagallidifolium Lam. 2 180 0.023 Lutz (2004)

Epilobium anagallidifolium Lam. 2 360 0.000 Lutz (2004)

Epilobium anagallidifolium Lam. 2 1440 0.000 Lutz (2004)

Epilobium anagallidifolium Lam. 3 0 1.000 Lutz (2004)

Epilobium anagallidifolium Lam. 3 1 0.026 Lutz (2004)

Epilobium anagallidifolium Lam. 3 5 0.026 Lutz (2004)

Epilobium anagallidifolium Lam. 3 10 0.026 Lutz (2004)

Epilobium anagallidifolium Lam. 3 30 0.026 Lutz (2004)

Epilobium anagallidifolium Lam. 3 60 0.013 Lutz (2004)

Epilobium anagallidifolium Lam. 3 120 0.000 Lutz (2004)

Epilobium anagallidifolium Lam. 3 180 0.000 Lutz (2004)

Epilobium anagallidifolium Lam. 3 360 0.000 Lutz (2004)

Epilobium anagallidifolium Lam. 3 1440 0.000 Lutz (2004)

Epilobium anagallidifolium Lam. 4 0 1.000 Lutz (2004)

Epilobium anagallidifolium Lam. 4 1 0.053 Lutz (2004)

Epilobium anagallidifolium Lam. 4 5 0.053 Lutz (2004)

Epilobium anagallidifolium Lam. 4 10 0.053 Lutz (2004)

Epilobium anagallidifolium Lam. 4 30 0.053 Lutz (2004)

Epilobium anagallidifolium Lam. 4 60 0.053 Lutz (2004)

Epilobium anagallidifolium Lam. 4 120 0.000 Lutz (2004)

Epilobium anagallidifolium Lam. 4 180 0.000 Lutz (2004)

Epilobium anagallidifolium Lam. 4 360 0.000 Lutz (2004)

Epilobium anagallidifolium Lam. 4 1440 0.000 Lutz (2004)

Epilobium anagallidifolium Lam. 5 0 1.000 Lutz (2004)

Epilobium anagallidifolium Lam. 5 1 0.255 Lutz (2004)

Epilobium anagallidifolium Lam. 5 5 0.235 Lutz (2004)

Epilobium anagallidifolium Lam. 5 10 0.118 Lutz (2004)

Epilobium anagallidifolium Lam. 5 30 0.118 Lutz (2004)

Epilobium anagallidifolium Lam. 5 60 0.098 Lutz (2004)

Epilobium anagallidifolium Lam. 5 120 0.000 Lutz (2004)

Epilobium anagallidifolium Lam. 5 180 0.000 Lutz (2004)

Epilobium anagallidifolium Lam. 5 360 0.000 Lutz (2004)

Epilobium anagallidifolium Lam. 5 1440 0.000 Lutz (2004)

Epilobium fleischeri Hochst. 1 0 1.000 Lutz (2004)

Epilobium fleischeri Hochst. 1 1 0.153 Lutz (2004)

Epilobium fleischeri Hochst. 1 5 0.082 Lutz (2004)

Epilobium fleischeri Hochst. 1 10 0.071 Lutz (2004)

Epilobium fleischeri Hochst. 1 30 0.071 Lutz (2004)

Epilobium fleischeri Hochst. 1 60 0.071 Lutz (2004)

Epilobium fleischeri Hochst. 1 120 0.010 Lutz (2004)

Epilobium fleischeri Hochst. 1 180 0.000 Lutz (2004)

Epilobium fleischeri Hochst. 1 360 0.000 Lutz (2004)

Epilobium fleischeri Hochst. 1 1440 0.000 Lutz (2004)

Epilobium fleischeri Hochst. 2 0 1.000 Lutz (2004)

Epilobium fleischeri Hochst. 2 1 0.347 Lutz (2004)

Epilobium fleischeri Hochst. 2 5 0.245 Lutz (2004)

Epilobium fleischeri Hochst. 2 10 0.235 Lutz (2004)

Epilobium fleischeri Hochst. 2 30 0.235 Lutz (2004)

Epilobium fleischeri Hochst. 2 60 0.235 Lutz (2004)

Epilobium fleischeri Hochst. 2 120 0.010 Lutz (2004)

Epilobium fleischeri Hochst. 2 180 0.000 Lutz (2004)

Epilobium fleischeri Hochst. 2 360 0.000 Lutz (2004)

Epilobium fleischeri Hochst. 2 1440 0.000 Lutz (2004)

Epilobium fleischeri Hochst. 3 0 1.000 Lutz (2004)

Epilobium fleischeri Hochst. 3 1 0.050 Lutz (2004)

Epilobium fleischeri Hochst. 3 5 0.030 Lutz (2004)

Epilobium fleischeri Hochst. 3 10 0.030 Lutz (2004)

Epilobium fleischeri Hochst. 3 30 0.030 Lutz (2004)

Epilobium fleischeri Hochst. 3 60 0.030 Lutz (2004)

Epilobium fleischeri Hochst. 3 120 0.010 Lutz (2004)

Epilobium fleischeri Hochst. 3 180 0.000 Lutz (2004)

Epilobium fleischeri Hochst. 3 360 0.000 Lutz (2004)

Epilobium fleischeri Hochst. 3 1440 0.000 Lutz (2004)

Epilobium fleischeri Hochst. 4 0 1.000 Lutz (2004)

Epilobium fleischeri Hochst. 4 1 0.079 Lutz (2004)

Epilobium fleischeri Hochst. 4 5 0.056 Lutz (2004)

Epilobium fleischeri Hochst. 4 10 0.056 Lutz (2004)

Epilobium fleischeri Hochst. 4 30 0.056 Lutz (2004)

Epilobium fleischeri Hochst. 4 60 0.056 Lutz (2004)

Epilobium fleischeri Hochst. 4 120 0.000 Lutz (2004)

Epilobium fleischeri Hochst. 4 180 0.000 Lutz (2004)

Epilobium fleischeri Hochst. 4 360 0.000 Lutz (2004)

Epilobium fleischeri Hochst. 4 1440 0.000 Lutz (2004)

Epilobium fleischeri Hochst. 5 0 1.000 Lutz (2004)

Epilobium fleischeri Hochst. 5 1 0.083 Lutz (2004)

Epilobium fleischeri Hochst. 5 5 0.031 Lutz (2004)

Epilobium fleischeri Hochst. 5 10 0.031 Lutz (2004)

Epilobium fleischeri Hochst. 5 30 0.021 Lutz (2004)

Epilobium fleischeri Hochst. 5 60 0.010 Lutz (2004)

Epilobium fleischeri Hochst. 5 120 0.000 Lutz (2004)

Epilobium fleischeri Hochst. 5 180 0.000 Lutz (2004)

Epilobium fleischeri Hochst. 5 360 0.000 Lutz (2004)

Epilobium fleischeri Hochst. 5 1440 0.000 Lutz (2004)

Festuca duvalii (St.-Yves) Stohr 1 0 1.000 Pirzer (2007)

Festuca duvalii (St.-Yves) Stohr 1 1 0.733 Pirzer (2007)

Festuca duvalii (St.-Yves) Stohr 1 5 0.700 Pirzer (2007)

Festuca duvalii (St.-Yves) Stohr 1 10 0.633 Pirzer (2007)

Festuca duvalii (St.-Yves) Stohr 1 30 0.600 Pirzer (2007)

Festuca duvalii (St.-Yves) Stohr 1 60 0.600 Pirzer (2007)

Festuca duvalii (St.-Yves) Stohr 1 120 0.133 Pirzer (2007)

Festuca duvalii (St.-Yves) Stohr 1 180 0.000 Pirzer (2007)

Festuca duvalii (St.-Yves) Stohr 1 360 0.000 Pirzer (2007)

Festuca duvalii (St.-Yves) Stohr 1 1440 0.000 Pirzer (2007)

Festuca duvalii (St.-Yves) Stohr 2 0 1.000 Pirzer (2007)

Festuca duvalii (St.-Yves) Stohr 2 1 0.571 Pirzer (2007)

Festuca duvalii (St.-Yves) Stohr 2 5 0.571 Pirzer (2007)

Festuca duvalii (St.-Yves) Stohr 2 10 0.536 Pirzer (2007)

Festuca duvalii (St.-Yves) Stohr 2 30 0.429 Pirzer (2007)

Festuca duvalii (St.-Yves) Stohr 2 60 0.429 Pirzer (2007)

Festuca duvalii (St.-Yves) Stohr 2 120 0.321 Pirzer (2007)

Festuca duvalii (St.-Yves) Stohr 2 180 0.214 Pirzer (2007)

Festuca duvalii (St.-Yves) Stohr 2 360 0.107 Pirzer (2007)

Festuca duvalii (St.-Yves) Stohr 2 1440 0.000 Pirzer (2007)

Festuca duvalii (St.-Yves) Stohr 3 0 1.000 Pirzer (2007)

Festuca duvalii (St.-Yves) Stohr 3 1 0.800 Pirzer (2007)

Festuca duvalii (St.-Yves) Stohr 3 5 0.800 Pirzer (2007)

Festuca duvalii (St.-Yves) Stohr 3 10 0.800 Pirzer (2007)

Festuca duvalii (St.-Yves) Stohr 3 30 0.800 Pirzer (2007)

Festuca duvalii (St.-Yves) Stohr 3 60 0.800 Pirzer (2007)

Festuca duvalii (St.-Yves) Stohr 3 120 0.800 Pirzer (2007)

Festuca duvalii (St.-Yves) Stohr 3 180 0.200 Pirzer (2007)

Festuca duvalii (St.-Yves) Stohr 3 360 0.000 Pirzer (2007)

Festuca duvalii (St.-Yves) Stohr 3 1440 0.000 Pirzer (2007)

Festuca duvalii (St.-Yves) Stohr 4 0 1.000 Pirzer (2007)

Festuca duvalii (St.-Yves) Stohr 4 1 0.417 Pirzer (2007)

Festuca duvalii (St.-Yves) Stohr 4 5 0.250 Pirzer (2007)

Festuca duvalii (St.-Yves) Stohr 4 10 0.208 Pirzer (2007)

Festuca duvalii (St.-Yves) Stohr 4 30 0.167 Pirzer (2007)

Festuca duvalii (St.-Yves) Stohr 4 60 0.125 Pirzer (2007)

Festuca duvalii (St.-Yves) Stohr 4 120 0.042 Pirzer (2007)

Festuca duvalii (St.-Yves) Stohr 4 180 0.042 Pirzer (2007)

Festuca duvalii (St.-Yves) Stohr 4 360 0.000 Pirzer (2007)

Festuca duvalii (St.-Yves) Stohr 4 1440 0.000 Pirzer (2007)

Festuca duvalii (St.-Yves) Stohr 5 0 1.000 Pirzer (2007)

Festuca duvalii (St.-Yves) Stohr 5 1 0.361 Pirzer (2007)

Festuca duvalii (St.-Yves) Stohr 5 5 0.333 Pirzer (2007)

Festuca duvalii (St.-Yves) Stohr 5 10 0.333 Pirzer (2007)

Festuca duvalii (St.-Yves) Stohr 5 30 0.333 Pirzer (2007)

Festuca duvalii (St.-Yves) Stohr 5 60 0.333 Pirzer (2007)

Festuca duvalii (St.-Yves) Stohr 5 120 0.250 Pirzer (2007)

Festuca duvalii (St.-Yves) Stohr 5 180 0.111 Pirzer (2007)

Festuca duvalii (St.-Yves) Stohr 5 360 0.028 Pirzer (2007)

Festuca duvalii (St.-Yves) Stohr 5 1440 0.000 Pirzer (2007)

Festuca guestfalica Boenn. ex Rchb. 1 0 1.000 Pirzer (2007)

Festuca guestfalica Boenn. ex Rchb. 1 1 0.864 Pirzer (2007)

Festuca guestfalica Boenn. ex Rchb. 1 5 0.864 Pirzer (2007)

Festuca guestfalica Boenn. ex Rchb. 1 10 0.841 Pirzer (2007)

Festuca guestfalica Boenn. ex Rchb. 1 30 0.841 Pirzer (2007)

Festuca guestfalica Boenn. ex Rchb. 1 60 0.841 Pirzer (2007)

Festuca guestfalica Boenn. ex Rchb. 1 120 0.273 Pirzer (2007)

Festuca guestfalica Boenn. ex Rchb. 1 180 0.227 Pirzer (2007)

Festuca guestfalica Boenn. ex Rchb. 1 360 0.023 Pirzer (2007)

Festuca guestfalica Boenn. ex Rchb. 1 1440 0.000 Pirzer (2007)

Festuca guestfalica Boenn. ex Rchb. 2 0 1.000 Pirzer (2007)

Festuca guestfalica Boenn. ex Rchb. 2 1 0.422 Pirzer (2007)

Festuca guestfalica Boenn. ex Rchb. 2 5 0.356 Pirzer (2007)

Festuca guestfalica Boenn. ex Rchb. 2 10 0.311 Pirzer (2007)

Festuca guestfalica Boenn. ex Rchb. 2 30 0.222 Pirzer (2007)

Festuca guestfalica Boenn. ex Rchb. 2 60 0.222 Pirzer (2007)

Festuca guestfalica Boenn. ex Rchb. 2 120 0.044 Pirzer (2007)

Festuca guestfalica Boenn. ex Rchb. 2 180 0.000 Pirzer (2007)

Festuca guestfalica Boenn. ex Rchb. 2 360 0.000 Pirzer (2007)

Festuca guestfalica Boenn. ex Rchb. 2 1440 0.000 Pirzer (2007)

Festuca guestfalica Boenn. ex Rchb. 3 0 1.000 Pirzer (2007)

Festuca guestfalica Boenn. ex Rchb. 3 1 0.654 Pirzer (2007)

Festuca guestfalica Boenn. ex Rchb. 3 5 0.577 Pirzer (2007)

Festuca guestfalica Boenn. ex Rchb. 3 10 0.538 Pirzer (2007)

Festuca guestfalica Boenn. ex Rchb. 3 30 0.500 Pirzer (2007)

Festuca guestfalica Boenn. ex Rchb. 3 60 0.500 Pirzer (2007)

Festuca guestfalica Boenn. ex Rchb. 3 120 0.154 Pirzer (2007)

Festuca guestfalica Boenn. ex Rchb. 3 180 0.077 Pirzer (2007)

Festuca guestfalica Boenn. ex Rchb. 3 360 0.000 Pirzer (2007)

Festuca guestfalica Boenn. ex Rchb. 3 1440 0.000 Pirzer (2007)

Festuca guestfalica Boenn. ex Rchb. 4 0 1.000 Pirzer (2007)

Festuca guestfalica Boenn. ex Rchb. 4 1 0.655 Pirzer (2007)

Festuca guestfalica Boenn. ex Rchb. 4 5 0.655 Pirzer (2007)

Festuca guestfalica Boenn. ex Rchb. 4 10 0.621 Pirzer (2007)

Festuca guestfalica Boenn. ex Rchb. 4 30 0.621 Pirzer (2007)

Festuca guestfalica Boenn. ex Rchb. 4 60 0.517 Pirzer (2007)

Festuca guestfalica Boenn. ex Rchb. 4 120 0.310 Pirzer (2007)

Festuca guestfalica Boenn. ex Rchb. 4 180 0.138 Pirzer (2007)

Festuca guestfalica Boenn. ex Rchb. 4 360 0.000 Pirzer (2007)

Festuca guestfalica Boenn. ex Rchb. 4 1440 0.000 Pirzer (2007)

Festuca guestfalica Boenn. ex Rchb. 5 0 1.000 Pirzer (2007)

Festuca guestfalica Boenn. ex Rchb. 5 1 0.618 Pirzer (2007)

Festuca guestfalica Boenn. ex Rchb. 5 5 0.618 Pirzer (2007)

Festuca guestfalica Boenn. ex Rchb. 5 10 0.588 Pirzer (2007)

Festuca guestfalica Boenn. ex Rchb. 5 30 0.559 Pirzer (2007)

Festuca guestfalica Boenn. ex Rchb. 5 60 0.559 Pirzer (2007)

Festuca guestfalica Boenn. ex Rchb. 5 120 0.206 Pirzer (2007)

Festuca guestfalica Boenn. ex Rchb. 5 180 0.118 Pirzer (2007)

Festuca guestfalica Boenn. ex Rchb. 5 360 0.088 Pirzer (2007)

Festuca guestfalica Boenn. ex Rchb. 5 1440 0.000 Pirzer (2007)

Festuca ovina agg. 1 0 1.000 original measurement

Festuca ovina agg. 1 1 0.071 original measurement

Festuca ovina agg. 1 5 0.071 original measurement

Festuca ovina agg. 1 10 0.048 original measurement

Festuca ovina agg. 1 30 0.000 original measurement

Festuca ovina agg. 1 60 0.000 original measurement

Festuca ovina agg. 1 120 0.000 original measurement

Festuca ovina agg. 1 180 0.000 original measurement

Festuca ovina agg. 2 0 1.000 original measurement

Festuca ovina agg. 2 1 0.077 original measurement

Festuca ovina agg. 2 5 0.038 original measurement

Festuca ovina agg. 2 10 0.038 original measurement

Festuca ovina agg. 2 30 0.038 original measurement

Festuca ovina agg. 2 60 0.038 original measurement

Festuca ovina agg. 2 120 0.038 original measurement

Festuca ovina agg. 2 180 0.000 original measurement

Festuca ovina agg. 2 360 0.000 original measurement

Festuca ovina agg. 2 1440 0.000 original measurement

Festuca ovina agg. 3 0 1.000 original measurement

Festuca ovina agg. 3 1 0.164 original measurement

Festuca ovina agg. 3 5 0.134 original measurement

Festuca ovina agg. 3 10 0.119 original measurement

Festuca ovina agg. 3 30 0.104 original measurement

Festuca ovina agg. 3 60 0.104 original measurement

Festuca ovina agg. 3 120 0.060 original measurement

Festuca ovina agg. 3 180 0.030 original measurement

Festuca ovina agg. 3 360 0.000 original measurement

Festuca ovina agg. 3 1440 0.000 original measurement

Festuca ovina agg. 4 0 1.000 original measurement

Festuca ovina agg. 4 1 0.241 original measurement

Festuca ovina agg. 4 5 0.172 original measurement

Festuca ovina agg. 4 10 0.155 original measurement

Festuca ovina agg. 4 30 0.138 original measurement

Festuca ovina agg. 4 60 0.138 original measurement

Festuca ovina agg. 4 120 0.086 original measurement

Festuca ovina agg. 4 180 0.069 original measurement

Festuca ovina agg. 4 360 0.000 original measurement

Festuca ovina agg. 4 1440 0.000 original measurement

Festuca pratensis Huds. 1 0 1.000 Pirzer (2007)

Festuca pratensis Huds. 1 1 0.923 Pirzer (2007)

Festuca pratensis Huds. 1 5 0.769 Pirzer (2007)

Festuca pratensis Huds. 1 10 0.769 Pirzer (2007)

Festuca pratensis Huds. 1 30 0.769 Pirzer (2007)

Festuca pratensis Huds. 1 60 0.769 Pirzer (2007)

Festuca pratensis Huds. 1 120 0.538 Pirzer (2007)

Festuca pratensis Huds. 1 180 0.154 Pirzer (2007)

Festuca pratensis Huds. 1 360 0.077 Pirzer (2007)

Festuca pratensis Huds. 1 1440 0.000 Pirzer (2007)

Festuca pratensis Huds. 2 0 1.000 Pirzer (2007)

Festuca pratensis Huds. 2 1 0.652 Pirzer (2007)

Festuca pratensis Huds. 2 5 0.609 Pirzer (2007)

Festuca pratensis Huds. 2 10 0.478 Pirzer (2007)

Festuca pratensis Huds. 2 30 0.391 Pirzer (2007)

Festuca pratensis Huds. 2 60 0.348 Pirzer (2007)

Festuca pratensis Huds. 2 120 0.130 Pirzer (2007)

Festuca pratensis Huds. 2 180 0.130 Pirzer (2007)

Festuca pratensis Huds. 2 360 0.000 Pirzer (2007)

Festuca pratensis Huds. 2 1440 0.000 Pirzer (2007)

Festuca pratensis Huds. 3 0 1.000 Pirzer (2007)

Festuca pratensis Huds. 3 1 0.458 Pirzer (2007)

Festuca pratensis Huds. 3 5 0.292 Pirzer (2007)

Festuca pratensis Huds. 3 10 0.292 Pirzer (2007)

Festuca pratensis Huds. 3 30 0.292 Pirzer (2007)

Festuca pratensis Huds. 3 60 0.292 Pirzer (2007)

Festuca pratensis Huds. 3 120 0.083 Pirzer (2007)

Festuca pratensis Huds. 3 180 0.083 Pirzer (2007)

Festuca pratensis Huds. 3 360 0.000 Pirzer (2007)

Festuca pratensis Huds. 3 1440 0.000 Pirzer (2007)

Festuca pratensis Huds. 4 0 1.000 Pirzer (2007)

Festuca pratensis Huds. 4 1 0.625 Pirzer (2007)

Festuca pratensis Huds. 4 5 0.563 Pirzer (2007)

Festuca pratensis Huds. 4 10 0.469 Pirzer (2007)

Festuca pratensis Huds. 4 30 0.469 Pirzer (2007)

Festuca pratensis Huds. 4 60 0.438 Pirzer (2007)

Festuca pratensis Huds. 4 120 0.031 Pirzer (2007)

Festuca pratensis Huds. 4 180 0.031 Pirzer (2007)

Festuca pratensis Huds. 4 360 0.000 Pirzer (2007)

Festuca pratensis Huds. 4 1440 0.000 Pirzer (2007)

Festuca pratensis Huds. 5 0 1.000 Pirzer (2007)

Festuca pratensis Huds. 5 1 0.429 Pirzer (2007)

Festuca pratensis Huds. 5 5 0.095 Pirzer (2007)

Festuca pratensis Huds. 5 10 0.048 Pirzer (2007)

Festuca pratensis Huds. 5 30 0.048 Pirzer (2007)

Festuca pratensis Huds. 5 60 0.048 Pirzer (2007)

Festuca pratensis Huds. 5 120 0.000 Pirzer (2007)

Festuca pratensis Huds. 5 180 0.000 Pirzer (2007)

Festuca pratensis Huds. 5 360 0.000 Pirzer (2007)

Festuca pratensis Huds. 5 1440 0.000 Pirzer (2007)

Festuca puccinellii Parl. 1 0 1.000 Pirzer (2007)

Festuca puccinellii Parl. 1 1 0.400 Pirzer (2007)

Festuca puccinellii Parl. 1 5 0.325 Pirzer (2007)

Festuca puccinellii Parl. 1 10 0.325 Pirzer (2007)

Festuca puccinellii Parl. 1 30 0.300 Pirzer (2007)

Festuca puccinellii Parl. 1 60 0.275 Pirzer (2007)

Festuca puccinellii Parl. 1 120 0.050 Pirzer (2007)

Festuca puccinellii Parl. 1 180 0.000 Pirzer (2007)

Festuca puccinellii Parl. 1 360 0.000 Pirzer (2007)

Festuca puccinellii Parl. 1 1440 0.000 Pirzer (2007)

Festuca puccinellii Parl. 2 0 1.000 Pirzer (2007)

Festuca puccinellii Parl. 2 1 0.882 Pirzer (2007)

Festuca puccinellii Parl. 2 5 0.882 Pirzer (2007)

Festuca puccinellii Parl. 2 10 0.882 Pirzer (2007)

Festuca puccinellii Parl. 2 30 0.882 Pirzer (2007)

Festuca puccinellii Parl. 2 60 0.882 Pirzer (2007)

Festuca puccinellii Parl. 2 120 0.529 Pirzer (2007)

Festuca puccinellii Parl. 2 180 0.176 Pirzer (2007)

Festuca puccinellii Parl. 2 360 0.059 Pirzer (2007)

Festuca puccinellii Parl. 2 1440 0.000 Pirzer (2007)

Festuca puccinellii Parl. 3 0 1.000 Pirzer (2007)

Festuca puccinellii Parl. 3 1 0.943 Pirzer (2007)

Festuca puccinellii Parl. 3 5 0.914 Pirzer (2007)

Festuca puccinellii Parl. 3 10 0.886 Pirzer (2007)

Festuca puccinellii Parl. 3 30 0.829 Pirzer (2007)

Festuca puccinellii Parl. 3 60 0.771 Pirzer (2007)

Festuca puccinellii Parl. 3 120 0.543 Pirzer (2007)

Festuca puccinellii Parl. 3 180 0.371 Pirzer (2007)

Festuca puccinellii Parl. 3 360 0.200 Pirzer (2007)

Festuca puccinellii Parl. 3 1440 0.000 Pirzer (2007)

Festuca puccinellii Parl. 4 0 1.000 Pirzer (2007)

Festuca puccinellii Parl. 4 1 0.750 Pirzer (2007)

Festuca puccinellii Parl. 4 5 0.643 Pirzer (2007)

Festuca puccinellii Parl. 4 10 0.643 Pirzer (2007)

Festuca puccinellii Parl. 4 30 0.429 Pirzer (2007)

Festuca puccinellii Parl. 4 60 0.429 Pirzer (2007)

Festuca puccinellii Parl. 4 120 0.036 Pirzer (2007)

Festuca puccinellii Parl. 4 180 0.036 Pirzer (2007)

Festuca puccinellii Parl. 4 360 0.000 Pirzer (2007)

Festuca puccinellii Parl. 4 1440 0.000 Pirzer (2007)

Festuca puccinellii Parl. 5 0 1.000 Pirzer (2007)

Festuca puccinellii Parl. 5 1 0.795 Pirzer (2007)

Festuca puccinellii Parl. 5 5 0.682 Pirzer (2007)

Festuca puccinellii Parl. 5 10 0.659 Pirzer (2007)

Festuca puccinellii Parl. 5 30 0.659 Pirzer (2007)

Festuca puccinellii Parl. 5 60 0.659 Pirzer (2007)

Festuca puccinellii Parl. 5 120 0.159 Pirzer (2007)

Festuca puccinellii Parl. 5 180 0.091 Pirzer (2007)

Festuca puccinellii Parl. 5 360 0.023 Pirzer (2007)

Festuca puccinellii Parl. 5 1440 0.000 Pirzer (2007)

Festuca valesiaca Schleich. ex Gaudin 1 0 1.000 Pirzer (2007)

Festuca valesiaca Schleich. ex Gaudin 1 1 0.625 Pirzer (2007)

Festuca valesiaca Schleich. ex Gaudin 1 5 0.575 Pirzer (2007)

Festuca valesiaca Schleich. ex Gaudin 1 10 0.550 Pirzer (2007)

Festuca valesiaca Schleich. ex Gaudin 1 30 0.500 Pirzer (2007)

Festuca valesiaca Schleich. ex Gaudin 1 60 0.475 Pirzer (2007)

Festuca valesiaca Schleich. ex Gaudin 1 120 0.275 Pirzer (2007)

Festuca valesiaca Schleich. ex Gaudin 1 180 0.150 Pirzer (2007)

Festuca valesiaca Schleich. ex Gaudin 1 360 0.050 Pirzer (2007)

Festuca valesiaca Schleich. ex Gaudin 1 1440 0.000 Pirzer (2007)

Festuca valesiaca Schleich. ex Gaudin 2 0 1.000 Pirzer (2007)

Festuca valesiaca Schleich. ex Gaudin 2 1 0.488 Pirzer (2007)

Festuca valesiaca Schleich. ex Gaudin 2 5 0.442 Pirzer (2007)

Festuca valesiaca Schleich. ex Gaudin 2 10 0.419 Pirzer (2007)

Festuca valesiaca Schleich. ex Gaudin 2 30 0.395 Pirzer (2007)

Festuca valesiaca Schleich. ex Gaudin 2 60 0.372 Pirzer (2007)

Festuca valesiaca Schleich. ex Gaudin 2 120 0.140 Pirzer (2007)

Festuca valesiaca Schleich. ex Gaudin 2 180 0.047 Pirzer (2007)

Festuca valesiaca Schleich. ex Gaudin 2 360 0.000 Pirzer (2007)

Festuca valesiaca Schleich. ex Gaudin 2 1440 0.000 Pirzer (2007)

Festuca valesiaca Schleich. ex Gaudin 3 0 1.000 Pirzer (2007)

Festuca valesiaca Schleich. ex Gaudin 3 1 0.612 Pirzer (2007)

Festuca valesiaca Schleich. ex Gaudin 3 5 0.551 Pirzer (2007)

Festuca valesiaca Schleich. ex Gaudin 3 10 0.510 Pirzer (2007)

Festuca valesiaca Schleich. ex Gaudin 3 30 0.408 Pirzer (2007)

Festuca valesiaca Schleich. ex Gaudin 3 60 0.388 Pirzer (2007)

Festuca valesiaca Schleich. ex Gaudin 3 120 0.224 Pirzer (2007)

Festuca valesiaca Schleich. ex Gaudin 3 180 0.184 Pirzer (2007)

Festuca valesiaca Schleich. ex Gaudin 3 360 0.102 Pirzer (2007)

Festuca valesiaca Schleich. ex Gaudin 3 1440 0.000 Pirzer (2007)

Festuca valesiaca Schleich. ex Gaudin 4 0 1.000 Pirzer (2007)

Festuca valesiaca Schleich. ex Gaudin 4 1 0.659 Pirzer (2007)

Festuca valesiaca Schleich. ex Gaudin 4 5 0.463 Pirzer (2007)

Festuca valesiaca Schleich. ex Gaudin 4 10 0.463 Pirzer (2007)

Festuca valesiaca Schleich. ex Gaudin 4 30 0.463 Pirzer (2007)

Festuca valesiaca Schleich. ex Gaudin 4 60 0.463 Pirzer (2007)

Festuca valesiaca Schleich. ex Gaudin 4 120 0.293 Pirzer (2007)

Festuca valesiaca Schleich. ex Gaudin 4 180 0.244 Pirzer (2007)

Festuca valesiaca Schleich. ex Gaudin 4 360 0.000 Pirzer (2007)

Festuca valesiaca Schleich. ex Gaudin 4 1440 0.000 Pirzer (2007)

Festuca valesiaca Schleich. ex Gaudin 5 0 1.000 Pirzer (2007)

Festuca valesiaca Schleich. ex Gaudin 5 1 0.815 Pirzer (2007)

Festuca valesiaca Schleich. ex Gaudin 5 5 0.741 Pirzer (2007)

Festuca valesiaca Schleich. ex Gaudin 5 10 0.704 Pirzer (2007)

Festuca valesiaca Schleich. ex Gaudin 5 30 0.704 Pirzer (2007)

Festuca valesiaca Schleich. ex Gaudin 5 60 0.630 Pirzer (2007)

Festuca valesiaca Schleich. ex Gaudin 5 120 0.556 Pirzer (2007)

Festuca valesiaca Schleich. ex Gaudin 5 180 0.407 Pirzer (2007)

Festuca valesiaca Schleich. ex Gaudin 5 360 0.000 Pirzer (2007)

Festuca valesiaca Schleich. ex Gaudin 5 1440 0.000 Pirzer (2007)

Gentiana punctata L. 1 0 1.000 Lutz (2004)

Gentiana punctata L. 1 1 0.031 Lutz (2004)

Gentiana punctata L. 1 5 0.031 Lutz (2004)

Gentiana punctata L. 1 10 0.031 Lutz (2004)

Gentiana punctata L. 1 30 0.031 Lutz (2004)

Gentiana punctata L. 1 60 0.031 Lutz (2004)

Gentiana punctata L. 1 120 0.031 Lutz (2004)

Gentiana punctata L. 1 180 0.015 Lutz (2004)

Gentiana punctata L. 1 360 0.000 Lutz (2004)

Gentiana punctata L. 1 1440 0.000 Lutz (2004)

Gentiana punctata L. 2 0 1.000 Lutz (2004)

Gentiana punctata L. 2 1 0.060 Lutz (2004)

Gentiana punctata L. 2 5 0.020 Lutz (2004)

Gentiana punctata L. 2 10 0.020 Lutz (2004)

Gentiana punctata L. 2 30 0.020 Lutz (2004)

Gentiana punctata L. 2 60 0.020 Lutz (2004)

Gentiana punctata L. 2 120 0.020 Lutz (2004)

Gentiana punctata L. 2 180 0.020 Lutz (2004)

Gentiana punctata L. 2 360 0.000 Lutz (2004)

Gentiana punctata L. 2 1440 0.000 Lutz (2004)

Gentiana punctata L. 3 0 1.000 Lutz (2004)

Gentiana punctata L. 3 1 0.067 Lutz (2004)

Gentiana punctata L. 3 5 0.040 Lutz (2004)

Gentiana punctata L. 3 10 0.040 Lutz (2004)

Gentiana punctata L. 3 30 0.027 Lutz (2004)

Gentiana punctata L. 3 60 0.027 Lutz (2004)

Gentiana punctata L. 3 120 0.000 Lutz (2004)

Gentiana punctata L. 3 180 0.000 Lutz (2004)

Gentiana punctata L. 3 360 0.000 Lutz (2004)

Gentiana punctata L. 3 1440 0.000 Lutz (2004)

Gentiana punctata L. 4 0 1.000 Lutz (2004)

Gentiana punctata L. 4 1 0.079 Lutz (2004)

Gentiana punctata L. 4 5 0.063 Lutz (2004)

Gentiana punctata L. 4 10 0.048 Lutz (2004)

Gentiana punctata L. 4 30 0.048 Lutz (2004)

Gentiana punctata L. 4 60 0.048 Lutz (2004)

Gentiana punctata L. 4 120 0.000 Lutz (2004)

Gentiana punctata L. 4 180 0.000 Lutz (2004)

Gentiana punctata L. 4 360 0.000 Lutz (2004)

Gentiana punctata L. 4 1440 0.000 Lutz (2004)

Gentiana punctata L. 5 0 1.000 Lutz (2004)

Gentiana punctata L. 5 1 0.025 Lutz (2004)

Gentiana punctata L. 5 5 0.025 Lutz (2004)

Gentiana punctata L. 5 10 0.025 Lutz (2004)

Gentiana punctata L. 5 30 0.025 Lutz (2004)

Gentiana punctata L. 5 60 0.025 Lutz (2004)

Gentiana punctata L. 5 120 0.000 Lutz (2004)

Gentiana punctata L. 5 180 0.000 Lutz (2004)

Gentiana punctata L. 5 360 0.000 Lutz (2004)

Gentiana punctata L. 5 1440 0.000 Lutz (2004)

Helianthemum nummularium s.l. (L.) Mill. 1 0 1.000 original measurement

Helianthemum nummularium s.l. (L.) Mill. 1 1 0.067 original measurement

Helianthemum nummularium s.l. (L.) Mill. 1 5 0.067 original measurement

Helianthemum nummularium s.l. (L.) Mill. 1 10 0.067 original measurement

Helianthemum nummularium s.l. (L.) Mill. 1 30 0.067 original measurement

Helianthemum nummularium s.l. (L.) Mill. 1 60 0.044 original measurement

Helianthemum nummularium s.l. (L.) Mill. 1 120 0.000 original measurement

Helianthemum nummularium s.l. (L.) Mill. 1 180 0.000 original measurement

Helianthemum nummularium s.l. (L.) Mill. 1 360 0.000 original measurement

Helianthemum nummularium s.l. (L.) Mill. 1 1440 0.000 original measurement

Helianthemum nummularium s.l. (L.) Mill. 2 0 1.000 original measurement

Helianthemum nummularium s.l. (L.) Mill. 2 1 0.091 original measurement

Helianthemum nummularium s.l. (L.) Mill. 2 5 0.036 original measurement

Helianthemum nummularium s.l. (L.) Mill. 2 10 0.036 original measurement

Helianthemum nummularium s.l. (L.) Mill. 2 30 0.036 original measurement

Helianthemum nummularium s.l. (L.) Mill. 2 60 0.036 original measurement

Helianthemum nummularium s.l. (L.) Mill. 2 120 0.036 original measurement

Helianthemum nummularium s.l. (L.) Mill. 2 180 0.018 original measurement

Helianthemum nummularium s.l. (L.) Mill. 2 360 0.018 original measurement

Helianthemum nummularium s.l. (L.) Mill. 2 1440 0.000 original measurement

Helianthemum nummularium s.l. (L.) Mill. 3 0 1.000 original measurement

Helianthemum nummularium s.l. (L.) Mill. 3 1 0.000 original measurement

Helianthemum nummularium s.l. (L.) Mill. 3 5 0.000 original measurement

Helianthemum nummularium s.l. (L.) Mill. 3 10 0.000 original measurement

Helianthemum nummularium s.l. (L.) Mill. 3 30 0.000 original measurement

Helianthemum nummularium s.l. (L.) Mill. 3 60 0.000 original measurement

Helianthemum nummularium s.l. (L.) Mill. 3 120 0.000 original measurement

Helianthemum nummularium s.l. (L.) Mill. 3 180 0.000 original measurement

Helianthemum nummularium s.l. (L.) Mill. 3 360 0.000 original measurement

Helianthemum nummularium s.l. (L.) Mill. 3 1440 0.000 original measurement

Helianthemum nummularium s.l. (L.) Mill. 4 0 1.000 original measurement

Helianthemum nummularium s.l. (L.) Mill. 4 1 0.123 original measurement

Helianthemum nummularium s.l. (L.) Mill. 4 5 0.070 original measurement

Helianthemum nummularium s.l. (L.) Mill. 4 10 0.070 original measurement

Helianthemum nummularium s.l. (L.) Mill. 4 30 0.035 original measurement

Helianthemum nummularium s.l. (L.) Mill. 4 60 0.018 original measurement

Helianthemum nummularium s.l. (L.) Mill. 4 120 0.018 original measurement

Helianthemum nummularium s.l. (L.) Mill. 4 180 0.000 original measurement

Helianthemum nummularium s.l. (L.) Mill. 4 360 0.000 original measurement

Helianthemum nummularium s.l. (L.) Mill. 4 1440 0.000 original measurement

Holcus lanatus L. 1 0 1.000 original measurement

Holcus lanatus L. 1 1 0.160 original measurement

Holcus lanatus L. 1 5 0.120 original measurement

Holcus lanatus L. 1 10 0.120 original measurement

Holcus lanatus L. 1 30 0.120 original measurement

Holcus lanatus L. 1 60 0.120 original measurement

Holcus lanatus L. 1 120 0.020 original measurement

Holcus lanatus L. 1 180 0.000 original measurement

Holcus lanatus L. 2 0 1.000 original measurement

Holcus lanatus L. 2 1 0.066 original measurement

Holcus lanatus L. 2 5 0.016 original measurement

Holcus lanatus L. 2 10 0.000 original measurement

Holcus lanatus L. 2 30 0.000 original measurement

Holcus lanatus L. 2 60 0.000 original measurement

Holcus lanatus L. 2 120 0.000 original measurement

Holcus lanatus L. 2 180 0.000 original measurement

Holcus lanatus L. 2 360 0.000 original measurement

Holcus lanatus L. 2 1440 0.000 original measurement

Holcus lanatus L. 3 0 1.000 original measurement

Holcus lanatus L. 3 1 0.205 original measurement

Holcus lanatus L. 3 5 0.157 original measurement

Holcus lanatus L. 3 10 0.133 original measurement

Holcus lanatus L. 3 30 0.133 original measurement

Holcus lanatus L. 3 60 0.133 original measurement

Holcus lanatus L. 3 120 0.120 original measurement

Holcus lanatus L. 3 180 0.120 original measurement

Holcus lanatus L. 3 360 0.012 original measurement

Holcus lanatus L. 3 1440 0.000 original measurement

Holcus lanatus L. 4 0 1.000 original measurement

Holcus lanatus L. 4 1 0.211 original measurement

Holcus lanatus L. 4 5 0.167 original measurement

Holcus lanatus L. 4 10 0.167 original measurement

Holcus lanatus L. 4 30 0.156 original measurement

Holcus lanatus L. 4 60 0.144 original measurement

Holcus lanatus L. 4 120 0.078 original measurement

Holcus lanatus L. 4 180 0.033 original measurement

Holcus lanatus L. 4 360 0.000 original measurement

Holcus lanatus L. 4 1440 0.000 original measurement

Homogyne alpina (L.) Cass. 1 0 1.000 Lutz (2004)

Homogyne alpina (L.) Cass. 1 1 0.370 Lutz (2004)

Homogyne alpina (L.) Cass. 1 5 0.304 Lutz (2004)

Homogyne alpina (L.) Cass. 1 10 0.261 Lutz (2004)

Homogyne alpina (L.) Cass. 1 30 0.217 Lutz (2004)

Homogyne alpina (L.) Cass. 1 60 0.217 Lutz (2004)

Homogyne alpina (L.) Cass. 1 120 0.087 Lutz (2004)

Homogyne alpina (L.) Cass. 1 180 0.022 Lutz (2004)

Homogyne alpina (L.) Cass. 1 360 0.000 Lutz (2004)

Homogyne alpina (L.) Cass. 1 1440 0.000 Lutz (2004)

Homogyne alpina (L.) Cass. 2 0 1.000 Lutz (2004)

Homogyne alpina (L.) Cass. 2 1 0.528 Lutz (2004)

Homogyne alpina (L.) Cass. 2 5 0.483 Lutz (2004)

Homogyne alpina (L.) Cass. 2 10 0.438 Lutz (2004)

Homogyne alpina (L.) Cass. 2 30 0.393 Lutz (2004)

Homogyne alpina (L.) Cass. 2 60 0.382 Lutz (2004)

Homogyne alpina (L.) Cass. 2 120 0.079 Lutz (2004)

Homogyne alpina (L.) Cass. 2 180 0.045 Lutz (2004)

Homogyne alpina (L.) Cass. 2 360 0.000 Lutz (2004)

Homogyne alpina (L.) Cass. 2 1440 0.000 Lutz (2004)

Homogyne alpina (L.) Cass. 3 0 1.000 Lutz (2004)

Homogyne alpina (L.) Cass. 3 1 0.268 Lutz (2004)

Homogyne alpina (L.) Cass. 3 5 0.113 Lutz (2004)

Homogyne alpina (L.) Cass. 3 10 0.113 Lutz (2004)

Homogyne alpina (L.) Cass. 3 30 0.103 Lutz (2004)

Homogyne alpina (L.) Cass. 3 60 0.103 Lutz (2004)

Homogyne alpina (L.) Cass. 3 120 0.000 Lutz (2004)

Homogyne alpina (L.) Cass. 3 180 0.000 Lutz (2004)

Homogyne alpina (L.) Cass. 3 360 0.000 Lutz (2004)

Homogyne alpina (L.) Cass. 3 1440 0.000 Lutz (2004)

Homogyne alpina (L.) Cass. 4 0 1.000 Lutz (2004)

Homogyne alpina (L.) Cass. 4 1 0.057 Lutz (2004)

Homogyne alpina (L.) Cass. 4 5 0.043 Lutz (2004)

Homogyne alpina (L.) Cass. 4 10 0.014 Lutz (2004)

Homogyne alpina (L.) Cass. 4 30 0.014 Lutz (2004)

Homogyne alpina (L.) Cass. 4 60 0.014 Lutz (2004)

Homogyne alpina (L.) Cass. 4 120 0.000 Lutz (2004)

Homogyne alpina (L.) Cass. 4 180 0.000 Lutz (2004)

Homogyne alpina (L.) Cass. 4 360 0.000 Lutz (2004)

Homogyne alpina (L.) Cass. 4 1440 0.000 Lutz (2004)

Homogyne alpina (L.) Cass. 5 0 1.000 Lutz (2004)

Homogyne alpina (L.) Cass. 5 1 0.118 Lutz (2004)

Homogyne alpina (L.) Cass. 5 5 0.118 Lutz (2004)

Homogyne alpina (L.) Cass. 5 10 0.103 Lutz (2004)

Homogyne alpina (L.) Cass. 5 30 0.088 Lutz (2004)

Homogyne alpina (L.) Cass. 5 60 0.088 Lutz (2004)

Homogyne alpina (L.) Cass. 5 120 0.059 Lutz (2004)

Homogyne alpina (L.) Cass. 5 180 0.015 Lutz (2004)

Homogyne alpina (L.) Cass. 5 360 0.000 Lutz (2004)

Homogyne alpina (L.) Cass. 5 1440 0.000 Lutz (2004)

Hypericum perforatum L. 1 0 1.000 original measurement

Hypericum perforatum L. 1 1 0.127 original measurement

Hypericum perforatum L. 1 5 0.036 original measurement

Hypericum perforatum L. 1 10 0.018 original measurement

Hypericum perforatum L. 1 30 0.000 original measurement

Hypericum perforatum L. 1 60 0.000 original measurement

Hypericum perforatum L. 1 120 0.000 original measurement

Hypericum perforatum L. 1 180 0.000 original measurement

Hypericum perforatum L. 1 360 0.000 original measurement

Hypericum perforatum L. 1 1440 0.000 original measurement

Hypericum perforatum L. 2 0 1.000 original measurement

Hypericum perforatum L. 2 1 0.151 original measurement

Hypericum perforatum L. 2 5 0.022 original measurement

Hypericum perforatum L. 2 10 0.000 original measurement

Hypericum perforatum L. 2 30 0.000 original measurement

Hypericum perforatum L. 2 60 0.000 original measurement

Hypericum perforatum L. 2 120 0.000 original measurement

Hypericum perforatum L. 2 180 0.000 original measurement

Hypericum perforatum L. 2 360 0.000 original measurement

Hypericum perforatum L. 2 1440 0.000 original measurement

Hypericum perforatum L. 3 0 1.000 original measurement

Hypericum perforatum L. 3 1 0.230 original measurement

Hypericum perforatum L. 3 5 0.189 original measurement

Hypericum perforatum L. 3 10 0.135 original measurement

Hypericum perforatum L. 3 30 0.095 original measurement

Hypericum perforatum L. 3 60 0.081 original measurement

Hypericum perforatum L. 3 120 0.041 original measurement

Hypericum perforatum L. 3 180 0.041 original measurement

Hypericum perforatum L. 3 360 0.000 original measurement

Hypericum perforatum L. 3 1440 0.000 original measurement

Hypericum perforatum L. 4 0 1.000 original measurement

Hypericum perforatum L. 4 1 0.250 original measurement

Hypericum perforatum L. 4 5 0.193 original measurement

Hypericum perforatum L. 4 10 0.182 original measurement

Hypericum perforatum L. 4 30 0.170 original measurement

Hypericum perforatum L. 4 60 0.148 original measurement

Hypericum perforatum L. 4 120 0.068 original measurement

Hypericum perforatum L. 4 180 0.023 original measurement

Hypericum perforatum L. 4 360 0.011 original measurement

Hypericum perforatum L. 4 1440 0.000 original measurement

Melica transsilvanica Schur 1 0 1.000 Pirzer (2007)

Melica transsilvanica Schur 1 1 0.000 Pirzer (2007)

Melica transsilvanica Schur 1 5 0.000 Pirzer (2007)

Melica transsilvanica Schur 1 10 0.000 Pirzer (2007)

Melica transsilvanica Schur 1 30 0.000 Pirzer (2007)

Melica transsilvanica Schur 1 60 0.000 Pirzer (2007)

Melica transsilvanica Schur 1 120 0.000 Pirzer (2007)

Melica transsilvanica Schur 1 180 0.000 Pirzer (2007)

Melica transsilvanica Schur 1 360 0.000 Pirzer (2007)

Melica transsilvanica Schur 1 1440 0.000 Pirzer (2007)

Melica transsilvanica Schur 2 0 1.000 Pirzer (2007)

Melica transsilvanica Schur 2 1 0.104 Pirzer (2007)

Melica transsilvanica Schur 2 5 0.021 Pirzer (2007)

Melica transsilvanica Schur 2 10 0.000 Pirzer (2007)

Melica transsilvanica Schur 2 30 0.000 Pirzer (2007)

Melica transsilvanica Schur 2 60 0.000 Pirzer (2007)

Melica transsilvanica Schur 2 120 0.000 Pirzer (2007)

Melica transsilvanica Schur 2 180 0.000 Pirzer (2007)

Melica transsilvanica Schur 2 360 0.000 Pirzer (2007)

Melica transsilvanica Schur 2 1440 0.000 Pirzer (2007)

Melica transsilvanica Schur 3 0 1.000 Pirzer (2007)

Melica transsilvanica Schur 3 1 0.350 Pirzer (2007)

Melica transsilvanica Schur 3 5 0.350 Pirzer (2007)

Melica transsilvanica Schur 3 10 0.350 Pirzer (2007)

Melica transsilvanica Schur 3 30 0.150 Pirzer (2007)

Melica transsilvanica Schur 3 60 0.150 Pirzer (2007)

Melica transsilvanica Schur 3 120 0.150 Pirzer (2007)

Melica transsilvanica Schur 3 180 0.100 Pirzer (2007)

Melica transsilvanica Schur 3 360 0.000 Pirzer (2007)

Melica transsilvanica Schur 3 1440 0.000 Pirzer (2007)

Melica transsilvanica Schur 4 0 1.000 Pirzer (2007)

Melica transsilvanica Schur 4 1 0.660 Pirzer (2007)

Melica transsilvanica Schur 4 5 0.638 Pirzer (2007)

Melica transsilvanica Schur 4 10 0.511 Pirzer (2007)

Melica transsilvanica Schur 4 30 0.489 Pirzer (2007)

Melica transsilvanica Schur 4 60 0.489 Pirzer (2007)

Melica transsilvanica Schur 4 120 0.000 Pirzer (2007)

Melica transsilvanica Schur 4 180 0.000 Pirzer (2007)

Melica transsilvanica Schur 4 360 0.000 Pirzer (2007)

Melica transsilvanica Schur 4 1440 0.000 Pirzer (2007)

Melica transsilvanica Schur 5 0 1.000 Pirzer (2007)

Melica transsilvanica Schur 5 1 0.303 Pirzer (2007)

Melica transsilvanica Schur 5 5 0.000 Pirzer (2007)

Melica transsilvanica Schur 5 10 0.000 Pirzer (2007)

Melica transsilvanica Schur 5 30 0.000 Pirzer (2007)

Melica transsilvanica Schur 5 60 0.000 Pirzer (2007)

Melica transsilvanica Schur 5 120 0.000 Pirzer (2007)

Melica transsilvanica Schur 5 180 0.000 Pirzer (2007)

Melica transsilvanica Schur 5 360 0.000 Pirzer (2007)

Melica transsilvanica Schur 5 1440 0.000 Pirzer (2007)

Myosotis alpestris F. W. Schmidt 1 0 1.000 Lutz (2004)

Myosotis alpestris F. W. Schmidt 1 1 0.119 Lutz (2004)

Myosotis alpestris F. W. Schmidt 1 5 0.107 Lutz (2004)

Myosotis alpestris F. W. Schmidt 1 10 0.107 Lutz (2004)

Myosotis alpestris F. W. Schmidt 1 30 0.107 Lutz (2004)

Myosotis alpestris F. W. Schmidt 1 60 0.107 Lutz (2004)

Myosotis alpestris F. W. Schmidt 1 120 0.024 Lutz (2004)

Myosotis alpestris F. W. Schmidt 1 180 0.000 Lutz (2004)

Myosotis alpestris F. W. Schmidt 1 360 0.000 Lutz (2004)

Myosotis alpestris F. W. Schmidt 1 1440 0.000 Lutz (2004)

Myosotis alpestris F. W. Schmidt 2 0 1.000 Lutz (2004)

Myosotis alpestris F. W. Schmidt 2 1 0.092 Lutz (2004)

Myosotis alpestris F. W. Schmidt 2 5 0.053 Lutz (2004)

Myosotis alpestris F. W. Schmidt 2 10 0.053 Lutz (2004)

Myosotis alpestris F. W. Schmidt 2 30 0.053 Lutz (2004)

Myosotis alpestris F. W. Schmidt 2 60 0.053 Lutz (2004)

Myosotis alpestris F. W. Schmidt 2 120 0.039 Lutz (2004)

Myosotis alpestris F. W. Schmidt 2 180 0.039 Lutz (2004)

Myosotis alpestris F. W. Schmidt 2 360 0.026 Lutz (2004)

Myosotis alpestris F. W. Schmidt 2 1440 0.000 Lutz (2004)

Myosotis alpestris F. W. Schmidt 3 0 1.000 Lutz (2004)

Myosotis alpestris F. W. Schmidt 3 1 0.056 Lutz (2004)

Myosotis alpestris F. W. Schmidt 3 5 0.056 Lutz (2004)

Myosotis alpestris F. W. Schmidt 3 10 0.056 Lutz (2004)

Myosotis alpestris F. W. Schmidt 3 30 0.056 Lutz (2004)

Myosotis alpestris F. W. Schmidt 3 60 0.042 Lutz (2004)

Myosotis alpestris F. W. Schmidt 3 120 0.028 Lutz (2004)

Myosotis alpestris F. W. Schmidt 3 180 0.014 Lutz (2004)

Myosotis alpestris F. W. Schmidt 3 360 0.000 Lutz (2004)

Myosotis alpestris F. W. Schmidt 3 1440 0.000 Lutz (2004)

Myosotis alpestris F. W. Schmidt 4 0 1.000 Lutz (2004)

Myosotis alpestris F. W. Schmidt 4 1 0.044 Lutz (2004)

Myosotis alpestris F. W. Schmidt 4 5 0.044 Lutz (2004)

Myosotis alpestris F. W. Schmidt 4 10 0.044 Lutz (2004)

Myosotis alpestris F. W. Schmidt 4 30 0.044 Lutz (2004)

Myosotis alpestris F. W. Schmidt 4 60 0.044 Lutz (2004)

Myosotis alpestris F. W. Schmidt 4 120 0.000 Lutz (2004)

Myosotis alpestris F. W. Schmidt 4 180 0.000 Lutz (2004)

Myosotis alpestris F. W. Schmidt 4 360 0.000 Lutz (2004)

Myosotis alpestris F. W. Schmidt 4 1440 0.000 Lutz (2004)

Myosotis alpestris F. W. Schmidt 5 0 1.000 Lutz (2004)

Myosotis alpestris F. W. Schmidt 5 1 0.055 Lutz (2004)

Myosotis alpestris F. W. Schmidt 5 5 0.055 Lutz (2004)

Myosotis alpestris F. W. Schmidt 5 10 0.055 Lutz (2004)

Myosotis alpestris F. W. Schmidt 5 30 0.036 Lutz (2004)

Myosotis alpestris F. W. Schmidt 5 60 0.036 Lutz (2004)

Myosotis alpestris F. W. Schmidt 5 120 0.000 Lutz (2004)

Myosotis alpestris F. W. Schmidt 5 180 0.000 Lutz (2004)

Myosotis alpestris F. W. Schmidt 5 360 0.000 Lutz (2004)

Myosotis alpestris F. W. Schmidt 5 1440 0.000 Lutz (2004)

Oxyria digyna (L.) Hill 1 0 1.000 Lutz (2004)

Oxyria digyna (L.) Hill 1 1 0.054 Lutz (2004)

Oxyria digyna (L.) Hill 1 5 0.036 Lutz (2004)

Oxyria digyna (L.) Hill 1 10 0.036 Lutz (2004)

Oxyria digyna (L.) Hill 1 30 0.018 Lutz (2004)

Oxyria digyna (L.) Hill 1 60 0.018 Lutz (2004)

Oxyria digyna (L.) Hill 1 120 0.000 Lutz (2004)

Oxyria digyna (L.) Hill 1 180 0.000 Lutz (2004)

Oxyria digyna (L.) Hill 1 360 0.000 Lutz (2004)

Oxyria digyna (L.) Hill 1 1440 0.000 Lutz (2004)

Oxyria digyna (L.) Hill 2 0 1.000 Lutz (2004)

Oxyria digyna (L.) Hill 2 1 0.139 Lutz (2004)

Oxyria digyna (L.) Hill 2 5 0.125 Lutz (2004)

Oxyria digyna (L.) Hill 2 10 0.125 Lutz (2004)

Oxyria digyna (L.) Hill 2 30 0.125 Lutz (2004)

Oxyria digyna (L.) Hill 2 60 0.097 Lutz (2004)

Oxyria digyna (L.) Hill 2 120 0.014 Lutz (2004)

Oxyria digyna (L.) Hill 2 180 0.014 Lutz (2004)

Oxyria digyna (L.) Hill 2 360 0.000 Lutz (2004)

Oxyria digyna (L.) Hill 2 1440 0.000 Lutz (2004)

Oxyria digyna (L.) Hill 3 0 1.000 Lutz (2004)

Oxyria digyna (L.) Hill 3 1 0.085 Lutz (2004)

Oxyria digyna (L.) Hill 3 5 0.037 Lutz (2004)

Oxyria digyna (L.) Hill 3 10 0.012 Lutz (2004)

Oxyria digyna (L.) Hill 3 30 0.012 Lutz (2004)

Oxyria digyna (L.) Hill 3 60 0.012 Lutz (2004)

Oxyria digyna (L.) Hill 3 120 0.000 Lutz (2004)

Oxyria digyna (L.) Hill 3 180 0.000 Lutz (2004)

Oxyria digyna (L.) Hill 3 360 0.000 Lutz (2004)

Oxyria digyna (L.) Hill 3 1440 0.000 Lutz (2004)

Oxyria digyna (L.) Hill 4 0 1.000 Lutz (2004)

Oxyria digyna (L.) Hill 4 1 0.029 Lutz (2004)

Oxyria digyna (L.) Hill 4 5 0.014 Lutz (2004)

Oxyria digyna (L.) Hill 4 10 0.000 Lutz (2004)

Oxyria digyna (L.) Hill 4 30 0.000 Lutz (2004)

Oxyria digyna (L.) Hill 4 60 0.000 Lutz (2004)

Oxyria digyna (L.) Hill 4 120 0.000 Lutz (2004)

Oxyria digyna (L.) Hill 4 180 0.000 Lutz (2004)

Oxyria digyna (L.) Hill 4 360 0.000 Lutz (2004)

Oxyria digyna (L.) Hill 4 1440 0.000 Lutz (2004)

Oxyria digyna (L.) Hill 5 0 1.000 Lutz (2004)

Oxyria digyna (L.) Hill 5 1 0.034 Lutz (2004)

Oxyria digyna (L.) Hill 5 5 0.034 Lutz (2004)

Oxyria digyna (L.) Hill 5 10 0.034 Lutz (2004)

Oxyria digyna (L.) Hill 5 30 0.017 Lutz (2004)

Oxyria digyna (L.) Hill 5 60 0.017 Lutz (2004)

Oxyria digyna (L.) Hill 5 120 0.000 Lutz (2004)

Oxyria digyna (L.) Hill 5 180 0.000 Lutz (2004)

Oxyria digyna (L.) Hill 5 360 0.000 Lutz (2004)

Oxyria digyna (L.) Hill 5 1440 0.000 Lutz (2004)

Peucedanum ostruthium (L.) Koch 1 0 1.000 Lutz (2004)

Peucedanum ostruthium (L.) Koch 1 1 0.179 Lutz (2004)

Peucedanum ostruthium (L.) Koch 1 5 0.128 Lutz (2004)

Peucedanum ostruthium (L.) Koch 1 10 0.103 Lutz (2004)

Peucedanum ostruthium (L.) Koch 1 30 0.103 Lutz (2004)

Peucedanum ostruthium (L.) Koch 1 60 0.026 Lutz (2004)

Peucedanum ostruthium (L.) Koch 1 120 0.026 Lutz (2004)

Peucedanum ostruthium (L.) Koch 1 180 0.026 Lutz (2004)

Peucedanum ostruthium (L.) Koch 1 360 0.000 Lutz (2004)

Peucedanum ostruthium (L.) Koch 1 1440 0.000 Lutz (2004)

Peucedanum ostruthium (L.) Koch 2 0 1.000 Lutz (2004)

Peucedanum ostruthium (L.) Koch 2 1 0.133 Lutz (2004)

Peucedanum ostruthium (L.) Koch 2 5 0.108 Lutz (2004)

Peucedanum ostruthium (L.) Koch 2 10 0.084 Lutz (2004)

Peucedanum ostruthium (L.) Koch 2 30 0.084 Lutz (2004)

Peucedanum ostruthium (L.) Koch 2 60 0.084 Lutz (2004)

Peucedanum ostruthium (L.) Koch 2 120 0.036 Lutz (2004)

Peucedanum ostruthium (L.) Koch 2 180 0.024 Lutz (2004)

Peucedanum ostruthium (L.) Koch 2 360 0.000 Lutz (2004)

Peucedanum ostruthium (L.) Koch 2 1440 0.000 Lutz (2004)

Peucedanum ostruthium (L.) Koch 3 0 1.000 Lutz (2004)

Peucedanum ostruthium (L.) Koch 3 1 0.000 Lutz (2004)

Peucedanum ostruthium (L.) Koch 3 5 0.000 Lutz (2004)

Peucedanum ostruthium (L.) Koch 3 10 0.000 Lutz (2004)

Peucedanum ostruthium (L.) Koch 3 30 0.000 Lutz (2004)

Peucedanum ostruthium (L.) Koch 3 60 0.000 Lutz (2004)

Peucedanum ostruthium (L.) Koch 3 120 0.000 Lutz (2004)

Peucedanum ostruthium (L.) Koch 3 180 0.000 Lutz (2004)

Peucedanum ostruthium (L.) Koch 3 360 0.000 Lutz (2004)

Peucedanum ostruthium (L.) Koch 3 1440 0.000 Lutz (2004)

Peucedanum ostruthium (L.) Koch 4 0 1.000 Lutz (2004)

Peucedanum ostruthium (L.) Koch 4 1 0.000 Lutz (2004)

Peucedanum ostruthium (L.) Koch 4 5 0.000 Lutz (2004)

Peucedanum ostruthium (L.) Koch 4 10 0.000 Lutz (2004)

Peucedanum ostruthium (L.) Koch 4 30 0.000 Lutz (2004)

Peucedanum ostruthium (L.) Koch 4 60 0.000 Lutz (2004)

Peucedanum ostruthium (L.) Koch 4 120 0.000 Lutz (2004)

Peucedanum ostruthium (L.) Koch 4 180 0.000 Lutz (2004)

Peucedanum ostruthium (L.) Koch 4 360 0.000 Lutz (2004)

Peucedanum ostruthium (L.) Koch 4 1440 0.000 Lutz (2004)

Peucedanum ostruthium (L.) Koch 5 0 1.000 Lutz (2004)

Peucedanum ostruthium (L.) Koch 5 1 0.040 Lutz (2004)

Peucedanum ostruthium (L.) Koch 5 5 0.000 Lutz (2004)

Peucedanum ostruthium (L.) Koch 5 10 0.000 Lutz (2004)

Peucedanum ostruthium (L.) Koch 5 30 0.000 Lutz (2004)

Peucedanum ostruthium (L.) Koch 5 60 0.000 Lutz (2004)

Peucedanum ostruthium (L.) Koch 5 120 0.000 Lutz (2004)

Peucedanum ostruthium (L.) Koch 5 180 0.000 Lutz (2004)

Peucedanum ostruthium (L.) Koch 5 360 0.000 Lutz (2004)

Peucedanum ostruthium (L.) Koch 5 1440 0.000 Lutz (2004)

Phleum rhaeticum (Humphries) Rauschert 1 0 1.000 Lutz (2004)

Phleum rhaeticum (Humphries) Rauschert 1 1 0.317 Lutz (2004)

Phleum rhaeticum (Humphries) Rauschert 1 5 0.238 Lutz (2004)

Phleum rhaeticum (Humphries) Rauschert 1 10 0.228 Lutz (2004)

Phleum rhaeticum (Humphries) Rauschert 1 30 0.198 Lutz (2004)

Phleum rhaeticum (Humphries) Rauschert 1 60 0.158 Lutz (2004)

Phleum rhaeticum (Humphries) Rauschert 1 120 0.059 Lutz (2004)

Phleum rhaeticum (Humphries) Rauschert 1 180 0.040 Lutz (2004)

Phleum rhaeticum (Humphries) Rauschert 1 360 0.000 Lutz (2004)

Phleum rhaeticum (Humphries) Rauschert 1 1440 0.000 Lutz (2004)

Phleum rhaeticum (Humphries) Rauschert 2 0 1.000 Lutz (2004)

Phleum rhaeticum (Humphries) Rauschert 2 1 0.271 Lutz (2004)

Phleum rhaeticum (Humphries) Rauschert 2 5 0.240 Lutz (2004)

Phleum rhaeticum (Humphries) Rauschert 2 10 0.240 Lutz (2004)

Phleum rhaeticum (Humphries) Rauschert 2 30 0.219 Lutz (2004)

Phleum rhaeticum (Humphries) Rauschert 2 60 0.219 Lutz (2004)

Phleum rhaeticum (Humphries) Rauschert 2 120 0.052 Lutz (2004)

Phleum rhaeticum (Humphries) Rauschert 2 180 0.010 Lutz (2004)

Phleum rhaeticum (Humphries) Rauschert 2 360 0.000 Lutz (2004)

Phleum rhaeticum (Humphries) Rauschert 2 1440 0.000 Lutz (2004)

Phleum rhaeticum (Humphries) Rauschert 3 0 1.000 Lutz (2004)

Phleum rhaeticum (Humphries) Rauschert 3 1 0.144 Lutz (2004)

Phleum rhaeticum (Humphries) Rauschert 3 5 0.082 Lutz (2004)

Phleum rhaeticum (Humphries) Rauschert 3 10 0.072 Lutz (2004)

Phleum rhaeticum (Humphries) Rauschert 3 30 0.052 Lutz (2004)

Phleum rhaeticum (Humphries) Rauschert 3 60 0.052 Lutz (2004)

Phleum rhaeticum (Humphries) Rauschert 3 120 0.041 Lutz (2004)

Phleum rhaeticum (Humphries) Rauschert 3 180 0.010 Lutz (2004)

Phleum rhaeticum (Humphries) Rauschert 3 360 0.000 Lutz (2004)

Phleum rhaeticum (Humphries) Rauschert 3 1440 0.000 Lutz (2004)

Phleum rhaeticum (Humphries) Rauschert 4 0 1.000 Lutz (2004)

Phleum rhaeticum (Humphries) Rauschert 4 1 0.116 Lutz (2004)

Phleum rhaeticum (Humphries) Rauschert 4 5 0.074 Lutz (2004)

Phleum rhaeticum (Humphries) Rauschert 4 10 0.074 Lutz (2004)

Phleum rhaeticum (Humphries) Rauschert 4 30 0.063 Lutz (2004)

Phleum rhaeticum (Humphries) Rauschert 4 60 0.042 Lutz (2004)

Phleum rhaeticum (Humphries) Rauschert 4 120 0.011 Lutz (2004)

Phleum rhaeticum (Humphries) Rauschert 4 180 0.011 Lutz (2004)

Phleum rhaeticum (Humphries) Rauschert 4 360 0.000 Lutz (2004)

Phleum rhaeticum (Humphries) Rauschert 4 1440 0.000 Lutz (2004)

Phleum rhaeticum (Humphries) Rauschert 5 0 1.000 Lutz (2004)

Phleum rhaeticum (Humphries) Rauschert 5 1 0.143 Lutz (2004)

Phleum rhaeticum (Humphries) Rauschert 5 5 0.122 Lutz (2004)

Phleum rhaeticum (Humphries) Rauschert 5 10 0.122 Lutz (2004)

Phleum rhaeticum (Humphries) Rauschert 5 30 0.092 Lutz (2004)

Phleum rhaeticum (Humphries) Rauschert 5 60 0.092 Lutz (2004)

Phleum rhaeticum (Humphries) Rauschert 5 120 0.041 Lutz (2004)

Phleum rhaeticum (Humphries) Rauschert 5 180 0.000 Lutz (2004)

Phleum rhaeticum (Humphries) Rauschert 5 360 0.000 Lutz (2004)

Phleum rhaeticum (Humphries) Rauschert 5 1440 0.000 Lutz (2004)

Phyteuma betonicifolium Vill. 1 0 1.000 Lutz (2004)

Phyteuma betonicifolium Vill. 1 1 0.291 Lutz (2004)

Phyteuma betonicifolium Vill. 1 5 0.256 Lutz (2004)

Phyteuma betonicifolium Vill. 1 10 0.221 Lutz (2004)

Phyteuma betonicifolium Vill. 1 30 0.186 Lutz (2004)

Phyteuma betonicifolium Vill. 1 60 0.186 Lutz (2004)

Phyteuma betonicifolium Vill. 1 120 0.128 Lutz (2004)

Phyteuma betonicifolium Vill. 1 180 0.105 Lutz (2004)

Phyteuma betonicifolium Vill. 1 360 0.012 Lutz (2004)

Phyteuma betonicifolium Vill. 1 1440 0.000 Lutz (2004)

Phyteuma betonicifolium Vill. 2 0 1.000 Lutz (2004)

Phyteuma betonicifolium Vill. 2 1 0.467 Lutz (2004)

Phyteuma betonicifolium Vill. 2 5 0.333 Lutz (2004)

Phyteuma betonicifolium Vill. 2 10 0.293 Lutz (2004)

Phyteuma betonicifolium Vill. 2 30 0.227 Lutz (2004)

Phyteuma betonicifolium Vill. 2 60 0.200 Lutz (2004)

Phyteuma betonicifolium Vill. 2 120 0.107 Lutz (2004)

Phyteuma betonicifolium Vill. 2 180 0.080 Lutz (2004)

Phyteuma betonicifolium Vill. 2 360 0.040 Lutz (2004)

Phyteuma betonicifolium Vill. 2 1440 0.000 Lutz (2004)

Phyteuma betonicifolium Vill. 3 0 1.000 Lutz (2004)

Phyteuma betonicifolium Vill. 3 1 0.155 Lutz (2004)

Phyteuma betonicifolium Vill. 3 5 0.107 Lutz (2004)

Phyteuma betonicifolium Vill. 3 10 0.095 Lutz (2004)

Phyteuma betonicifolium Vill. 3 30 0.095 Lutz (2004)

Phyteuma betonicifolium Vill. 3 60 0.083 Lutz (2004)

Phyteuma betonicifolium Vill. 3 120 0.000 Lutz (2004)

Phyteuma betonicifolium Vill. 3 180 0.000 Lutz (2004)

Phyteuma betonicifolium Vill. 3 360 0.000 Lutz (2004)

Phyteuma betonicifolium Vill. 3 1440 0.000 Lutz (2004)

Phyteuma betonicifolium Vill. 4 0 1.000 Lutz (2004)

Phyteuma betonicifolium Vill. 4 1 0.060 Lutz (2004)

Phyteuma betonicifolium Vill. 4 5 0.048 Lutz (2004)

Phyteuma betonicifolium Vill. 4 10 0.048 Lutz (2004)

Phyteuma betonicifolium Vill. 4 30 0.048 Lutz (2004)

Phyteuma betonicifolium Vill. 4 60 0.048 Lutz (2004)

Phyteuma betonicifolium Vill. 4 120 0.024 Lutz (2004)

Phyteuma betonicifolium Vill. 4 180 0.024 Lutz (2004)

Phyteuma betonicifolium Vill. 4 360 0.000 Lutz (2004)

Phyteuma betonicifolium Vill. 4 1440 0.000 Lutz (2004)

Phyteuma betonicifolium Vill. 5 1 1.000 Lutz (2004)

Phyteuma betonicifolium Vill. 5 5 0.923 Lutz (2004)

Phyteuma betonicifolium Vill. 5 10 0.846 Lutz (2004)

Phyteuma betonicifolium Vill. 5 30 0.769 Lutz (2004)

Phyteuma betonicifolium Vill. 5 60 0.538 Lutz (2004)

Phyteuma betonicifolium Vill. 5 120 0.231 Lutz (2004)

Phyteuma betonicifolium Vill. 5 180 0.000 Lutz (2004)

Phyteuma betonicifolium Vill. 5 360 0.000 Lutz (2004)

Phyteuma betonicifolium Vill. 5 1440 0.000 Lutz (2004)

Plantago media L. 1 0 1.000 original measurement

Plantago media L. 1 1 0.148 original measurement

Plantago media L. 1 5 0.016 original measurement

Plantago media L. 1 10 0.016 original measurement

Plantago media L. 1 30 0.000 original measurement

Plantago media L. 1 60 0.000 original measurement

Plantago media L. 1 120 0.000 original measurement

Plantago media L. 1 180 0.000 original measurement

Plantago media L. 1 360 0.000 original measurement

Plantago media L. 1 1440 0.000 original measurement

Plantago media L. 2 0 1.000 original measurement

Plantago media L. 2 1 0.276 original measurement

Plantago media L. 2 5 0.172 original measurement

Plantago media L. 2 10 0.149 original measurement

Plantago media L. 2 30 0.115 original measurement

Plantago media L. 2 60 0.115 original measurement

Plantago media L. 2 120 0.092 original measurement

Plantago media L. 2 180 0.023 original measurement

Plantago media L. 2 360 0.023 original measurement

Plantago media L. 2 1440 0.000 original measurement

Plantago media L. 3 0 1.000 original measurement

Plantago media L. 3 1 0.188 original measurement

Plantago media L. 3 5 0.125 original measurement

Plantago media L. 3 10 0.094 original measurement

Plantago media L. 3 30 0.078 original measurement

Plantago media L. 3 60 0.063 original measurement

Plantago media L. 3 120 0.031 original measurement

Plantago media L. 3 180 0.031 original measurement

Plantago media L. 3 360 0.031 original measurement

Plantago media L. 3 1440 0.000 original measurement

Plantago media L. 4 0 1.000 original measurement

Plantago media L. 4 1 0.143 original measurement

Plantago media L. 4 5 0.117 original measurement

Plantago media L. 4 10 0.117 original measurement

Plantago media L. 4 30 0.117 original measurement

Plantago media L. 4 60 0.104 original measurement

Plantago media L. 4 120 0.026 original measurement

Plantago media L. 4 180 0.026 original measurement

Plantago media L. 4 360 0.000 original measurement

Plantago media L. 4 1440 0.000 original measurement

Poa alpina L. 1 0 1.000 Lutz (2004)

Poa alpina L. 1 1 0.146 Lutz (2004)

Poa alpina L. 1 5 0.135 Lutz (2004)

Poa alpina L. 1 10 0.124 Lutz (2004)

Poa alpina L. 1 30 0.101 Lutz (2004)

Poa alpina L. 1 60 0.101 Lutz (2004)

Poa alpina L. 1 120 0.056 Lutz (2004)

Poa alpina L. 1 180 0.011 Lutz (2004)

Poa alpina L. 1 360 0.011 Lutz (2004)

Poa alpina L. 1 1440 0.000 Lutz (2004)

Poa alpina L. 2 0 1.000 Lutz (2004)

Poa alpina L. 2 1 0.135 Lutz (2004)

Poa alpina L. 2 5 0.135 Lutz (2004)

Poa alpina L. 2 10 0.101 Lutz (2004)

Poa alpina L. 2 30 0.101 Lutz (2004)

Poa alpina L. 2 60 0.101 Lutz (2004)

Poa alpina L. 2 120 0.011 Lutz (2004)

Poa alpina L. 2 180 0.011 Lutz (2004)

Poa alpina L. 2 360 0.000 Lutz (2004)

Poa alpina L. 2 1440 0.000 Lutz (2004)

Poa alpina L. 3 0 1.000 Lutz (2004)

Poa alpina L. 3 1 0.104 Lutz (2004)

Poa alpina L. 3 5 0.042 Lutz (2004)

Poa alpina L. 3 10 0.042 Lutz (2004)

Poa alpina L. 3 30 0.042 Lutz (2004)

Poa alpina L. 3 60 0.042 Lutz (2004)

Poa alpina L. 3 120 0.031 Lutz (2004)

Poa alpina L. 3 180 0.021 Lutz (2004)

Poa alpina L. 3 360 0.010 Lutz (2004)

Poa alpina L. 3 1440 0.000 Lutz (2004)

Poa alpina L. 4 0 1.000 Lutz (2004)

Poa alpina L. 4 1 0.089 Lutz (2004)

Poa alpina L. 4 5 0.076 Lutz (2004)

Poa alpina L. 4 10 0.063 Lutz (2004)

Poa alpina L. 4 30 0.063 Lutz (2004)

Poa alpina L. 4 60 0.063 Lutz (2004)

Poa alpina L. 4 120 0.025 Lutz (2004)

Poa alpina L. 4 180 0.013 Lutz (2004)

Poa alpina L. 4 360 0.013 Lutz (2004)

Poa alpina L. 4 1440 0.000 Lutz (2004)

Poa alpina L. 5 0 1.000 Lutz (2004)

Poa alpina L. 5 1 0.125 Lutz (2004)

Poa alpina L. 5 5 0.125 Lutz (2004)

Poa alpina L. 5 10 0.114 Lutz (2004)

Poa alpina L. 5 30 0.102 Lutz (2004)

Poa alpina L. 5 60 0.091 Lutz (2004)

Poa alpina L. 5 120 0.045 Lutz (2004)

Poa alpina L. 5 180 0.023 Lutz (2004)

Poa alpina L. 5 360 0.000 Lutz (2004)

Poa alpina L. 5 1440 0.000 Lutz (2004)

Poa bulbosa L. 1 0 1.000 Pirzer (2007)

Poa bulbosa L. 1 1 0.524 Pirzer (2007)

Poa bulbosa L. 1 5 0.524 Pirzer (2007)

Poa bulbosa L. 1 10 0.524 Pirzer (2007)

Poa bulbosa L. 1 30 0.524 Pirzer (2007)

Poa bulbosa L. 1 60 0.476 Pirzer (2007)

Poa bulbosa L. 1 120 0.000 Pirzer (2007)

Poa bulbosa L. 1 180 0.000 Pirzer (2007)

Poa bulbosa L. 1 360 0.000 Pirzer (2007)

Poa bulbosa L. 1 1440 0.000 Pirzer (2007)

Poa bulbosa L. 2 0 1.000 Pirzer (2007)

Poa bulbosa L. 2 1 0.160 Pirzer (2007)

Poa bulbosa L. 2 5 0.160 Pirzer (2007)

Poa bulbosa L. 2 10 0.160 Pirzer (2007)

Poa bulbosa L. 2 30 0.160 Pirzer (2007)

Poa bulbosa L. 2 60 0.160 Pirzer (2007)

Poa bulbosa L. 2 120 0.000 Pirzer (2007)

Poa bulbosa L. 2 180 0.000 Pirzer (2007)

Poa bulbosa L. 2 360 0.000 Pirzer (2007)

Poa bulbosa L. 2 1440 0.000 Pirzer (2007)

Poa bulbosa L. 3 0 1.000 Pirzer (2007)

Poa bulbosa L. 3 1 0.816 Pirzer (2007)

Poa bulbosa L. 3 5 0.789 Pirzer (2007)

Poa bulbosa L. 3 10 0.789 Pirzer (2007)

Poa bulbosa L. 3 30 0.789 Pirzer (2007)

Poa bulbosa L. 3 60 0.763 Pirzer (2007)

Poa bulbosa L. 3 120 0.184 Pirzer (2007)

Poa bulbosa L. 3 180 0.079 Pirzer (2007)

Poa bulbosa L. 3 360 0.000 Pirzer (2007)

Poa bulbosa L. 3 1440 0.000 Pirzer (2007)

Poa bulbosa L. 4 0 1.000 Pirzer (2007)

Poa bulbosa L. 4 1 0.381 Pirzer (2007)

Poa bulbosa L. 4 5 0.143 Pirzer (2007)

Poa bulbosa L. 4 10 0.143 Pirzer (2007)

Poa bulbosa L. 4 30 0.143 Pirzer (2007)

Poa bulbosa L. 4 60 0.143 Pirzer (2007)

Poa bulbosa L. 4 120 0.048 Pirzer (2007)

Poa bulbosa L. 4 180 0.048 Pirzer (2007)

Poa bulbosa L. 4 360 0.000 Pirzer (2007)

Poa bulbosa L. 4 1440 0.000 Pirzer (2007)

Poa bulbosa L. 5 0 1.000 Pirzer (2007)

Poa bulbosa L. 5 1 0.000 Pirzer (2007)

Poa bulbosa L. 5 5 0.000 Pirzer (2007)

Poa bulbosa L. 5 10 0.000 Pirzer (2007)

Poa bulbosa L. 5 30 0.000 Pirzer (2007)

Poa bulbosa L. 5 60 0.000 Pirzer (2007)

Poa bulbosa L. 5 120 0.000 Pirzer (2007)

Poa bulbosa L. 5 180 0.000 Pirzer (2007)

Poa bulbosa L. 5 360 0.000 Pirzer (2007)

Poa bulbosa L. 5 1440 0.000 Pirzer (2007)

Ranunculus acris L. 1 0 1.000 original measurement

Ranunculus acris L. 1 1 0.152 original measurement

Ranunculus acris L. 1 5 0.091 original measurement

Ranunculus acris L. 1 10 0.000 original measurement

Ranunculus acris L. 1 30 0.000 original measurement

Ranunculus acris L. 1 60 0.000 original measurement

Ranunculus acris L. 1 120 0.000 original measurement

Ranunculus acris L. 1 180 0.000 original measurement

Ranunculus acris L. 2 0 1.000 original measurement

Ranunculus acris L. 2 1 0.042 original measurement

Ranunculus acris L. 2 5 0.042 original measurement

Ranunculus acris L. 2 10 0.042 original measurement

Ranunculus acris L. 2 30 0.042 original measurement

Ranunculus acris L. 2 60 0.042 original measurement

Ranunculus acris L. 2 120 0.042 original measurement

Ranunculus acris L. 2 180 0.000 original measurement

Ranunculus acris L. 2 360 0.000 original measurement

Ranunculus acris L. 2 1440 0.000 original measurement

Ranunculus acris L. 3 0 1.000 original measurement

Ranunculus acris L. 3 1 0.256 original measurement

Ranunculus acris L. 3 5 0.231 original measurement

Ranunculus acris L. 3 10 0.231 original measurement

Ranunculus acris L. 3 30 0.231 original measurement

Ranunculus acris L. 3 60 0.231 original measurement

Ranunculus acris L. 3 120 0.154 original measurement

Ranunculus acris L. 3 180 0.128 original measurement

Ranunculus acris L. 3 360 0.026 original measurement

Ranunculus acris L. 3 1440 0.000 original measurement

Ranunculus acris L. 4 0 1.000 original measurement

Ranunculus acris L. 4 1 0.167 original measurement

Ranunculus acris L. 4 5 0.119 original measurement

Ranunculus acris L. 4 10 0.095 original measurement

Ranunculus acris L. 4 30 0.071 original measurement

Ranunculus acris L. 4 60 0.071 original measurement

Ranunculus acris L. 4 120 0.071 original measurement

Ranunculus acris L. 4 180 0.024 original measurement

Ranunculus acris L. 4 360 0.000 original measurement

Ranunculus acris L. 4 1440 0.000 original measurement

Ranunculus bulbosus L. 1 0 1.000 original measurement

Ranunculus bulbosus L. 1 1 0.071 original measurement

Ranunculus bulbosus L. 1 5 0.048 original measurement

Ranunculus bulbosus L. 1 10 0.048 original measurement

Ranunculus bulbosus L. 1 30 0.048 original measurement

Ranunculus bulbosus L. 1 60 0.048 original measurement

Ranunculus bulbosus L. 1 120 0.024 original measurement

Ranunculus bulbosus L. 1 180 0.000 original measurement

Ranunculus bulbosus L. 1 360 0.000 original measurement

Ranunculus bulbosus L. 1 1440 0.000 original measurement

Ranunculus bulbosus L. 2 0 1.000 original measurement

Ranunculus bulbosus L. 2 1 0.094 original measurement

Ranunculus bulbosus L. 2 5 0.063 original measurement

Ranunculus bulbosus L. 2 10 0.063 original measurement

Ranunculus bulbosus L. 2 30 0.047 original measurement

Ranunculus bulbosus L. 2 60 0.047 original measurement

Ranunculus bulbosus L. 2 120 0.031 original measurement

Ranunculus bulbosus L. 2 180 0.000 original measurement

Ranunculus bulbosus L. 2 360 0.000 original measurement

Ranunculus bulbosus L. 2 1440 0.000 original measurement

Ranunculus bulbosus L. 3 0 1.000 original measurement

Ranunculus bulbosus L. 3 1 0.095 original measurement

Ranunculus bulbosus L. 3 5 0.024 original measurement

Ranunculus bulbosus L. 3 10 0.000 original measurement

Ranunculus bulbosus L. 3 30 0.000 original measurement

Ranunculus bulbosus L. 3 60 0.000 original measurement

Ranunculus bulbosus L. 3 120 0.000 original measurement

Ranunculus bulbosus L. 3 180 0.000 original measurement

Ranunculus bulbosus L. 3 360 0.000 original measurement

Ranunculus bulbosus L. 3 1440 0.000 original measurement

Ranunculus bulbosus L. 4 0 1.000 original measurement

Ranunculus bulbosus L. 4 1 0.041 original measurement

Ranunculus bulbosus L. 4 5 0.041 original measurement

Ranunculus bulbosus L. 4 10 0.020 original measurement

Ranunculus bulbosus L. 4 30 0.020 original measurement

Ranunculus bulbosus L. 4 60 0.020 original measurement

Ranunculus bulbosus L. 4 120 0.000 original measurement

Ranunculus bulbosus L. 4 180 0.000 original measurement

Ranunculus bulbosus L. 4 360 0.000 original measurement

Ranunculus bulbosus L. 4 1440 0.000 original measurement

Rumex acetosella s.l. L. 1 0 1.000 original measurement

Rumex acetosella s.l. L. 1 1 0.056 original measurement

Rumex acetosella s.l. L. 1 5 0.028 original measurement

Rumex acetosella s.l. L. 1 10 0.014 original measurement

Rumex acetosella s.l. L. 1 30 0.014 original measurement

Rumex acetosella s.l. L. 1 60 0.000 original measurement

Rumex acetosella s.l. L. 1 120 0.000 original measurement

Rumex acetosella s.l. L. 1 180 0.000 original measurement

Rumex acetosella s.l. L. 1 360 0.000 original measurement

Rumex acetosella s.l. L. 1 1440 0.000 original measurement

Rumex acetosella s.l. L. 2 0 1.000 original measurement

Rumex acetosella s.l. L. 2 1 0.235 original measurement

Rumex acetosella s.l. L. 2 5 0.165 original measurement

Rumex acetosella s.l. L. 2 10 0.153 original measurement

Rumex acetosella s.l. L. 2 30 0.106 original measurement

Rumex acetosella s.l. L. 2 60 0.094 original measurement

Rumex acetosella s.l. L. 2 120 0.082 original measurement

Rumex acetosella s.l. L. 2 180 0.024 original measurement

Rumex acetosella s.l. L. 2 360 0.012 original measurement

Rumex acetosella s.l. L. 2 1440 0.000 original measurement

Rumex acetosella s.l. L. 3 0 1.000 original measurement

Rumex acetosella s.l. L. 3 1 0.224 original measurement

Rumex acetosella s.l. L. 3 5 0.138 original measurement

Rumex acetosella s.l. L. 3 10 0.138 original measurement

Rumex acetosella s.l. L. 3 30 0.138 original measurement

Rumex acetosella s.l. L. 3 60 0.138 original measurement

Rumex acetosella s.l. L. 3 120 0.086 original measurement

Rumex acetosella s.l. L. 3 180 0.052 original measurement

Rumex acetosella s.l. L. 3 360 0.000 original measurement

Rumex acetosella s.l. L. 3 1440 0.000 original measurement

Rumex acetosella s.l. L. 4 0 1.000 original measurement

Rumex acetosella s.l. L. 4 1 0.191 original measurement

Rumex acetosella s.l. L. 4 5 0.088 original measurement

Rumex acetosella s.l. L. 4 10 0.074 original measurement

Rumex acetosella s.l. L. 4 30 0.029 original measurement

Rumex acetosella s.l. L. 4 60 0.000 original measurement

Rumex acetosella s.l. L. 4 120 0.000 original measurement

Rumex acetosella s.l. L. 4 180 0.000 original measurement

Rumex acetosella s.l. L. 4 360 0.000 original measurement

Rumex acetosella s.l. L. 4 1440 0.000 original measurement

Sagina saginoides (L.) H. Karst. 1 0 1.000 Lutz (2004)

Sagina saginoides (L.) H. Karst. 1 1 0.333 Lutz (2004)

Sagina saginoides (L.) H. Karst. 1 5 0.256 Lutz (2004)

Sagina saginoides (L.) H. Karst. 1 10 0.233 Lutz (2004)

Sagina saginoides (L.) H. Karst. 1 30 0.211 Lutz (2004)

Sagina saginoides (L.) H. Karst. 1 60 0.189 Lutz (2004)

Sagina saginoides (L.) H. Karst. 1 120 0.089 Lutz (2004)

Sagina saginoides (L.) H. Karst. 1 180 0.011 Lutz (2004)

Sagina saginoides (L.) H. Karst. 1 360 0.000 Lutz (2004)

Sagina saginoides (L.) H. Karst. 1 1440 0.000 Lutz (2004)

Sagina saginoides (L.) H. Karst. 2 0 1.000 Lutz (2004)

Sagina saginoides (L.) H. Karst. 2 1 0.460 Lutz (2004)

Sagina saginoides (L.) H. Karst. 2 5 0.276 Lutz (2004)

Sagina saginoides (L.) H. Karst. 2 10 0.241 Lutz (2004)

Sagina saginoides (L.) H. Karst. 2 30 0.230 Lutz (2004)

Sagina saginoides (L.) H. Karst. 2 60 0.218 Lutz (2004)

Sagina saginoides (L.) H. Karst. 2 120 0.149 Lutz (2004)

Sagina saginoides (L.) H. Karst. 2 180 0.126 Lutz (2004)

Sagina saginoides (L.) H. Karst. 2 360 0.023 Lutz (2004)

Sagina saginoides (L.) H. Karst. 2 1440 0.000 Lutz (2004)

Sagina saginoides (L.) H. Karst. 3 0 1.000 Lutz (2004)

Sagina saginoides (L.) H. Karst. 3 1 0.333 Lutz (2004)

Sagina saginoides (L.) H. Karst. 3 5 0.219 Lutz (2004)

Sagina saginoides (L.) H. Karst. 3 10 0.188 Lutz (2004)

Sagina saginoides (L.) H. Karst. 3 30 0.167 Lutz (2004)

Sagina saginoides (L.) H. Karst. 3 60 0.156 Lutz (2004)

Sagina saginoides (L.) H. Karst. 3 120 0.073 Lutz (2004)

Sagina saginoides (L.) H. Karst. 3 180 0.031 Lutz (2004)

Sagina saginoides (L.) H. Karst. 3 360 0.021 Lutz (2004)

Sagina saginoides (L.) H. Karst. 3 1440 0.000 Lutz (2004)

Sagina saginoides (L.) H. Karst. 4 0 1.000 Lutz (2004)

Sagina saginoides (L.) H. Karst. 4 1 0.162 Lutz (2004)

Sagina saginoides (L.) H. Karst. 4 5 0.101 Lutz (2004)

Sagina saginoides (L.) H. Karst. 4 10 0.081 Lutz (2004)

Sagina saginoides (L.) H. Karst. 4 30 0.081 Lutz (2004)

Sagina saginoides (L.) H. Karst. 4 60 0.071 Lutz (2004)

Sagina saginoides (L.) H. Karst. 4 120 0.020 Lutz (2004)

Sagina saginoides (L.) H. Karst. 4 180 0.010 Lutz (2004)

Sagina saginoides (L.) H. Karst. 4 360 0.000 Lutz (2004)

Sagina saginoides (L.) H. Karst. 4 1440 0.000 Lutz (2004)

Sagina saginoides (L.) H. Karst. 5 0 1.000 Lutz (2004)

Sagina saginoides (L.) H. Karst. 5 1 0.208 Lutz (2004)

Sagina saginoides (L.) H. Karst. 5 5 0.143 Lutz (2004)

Sagina saginoides (L.) H. Karst. 5 10 0.130 Lutz (2004)

Sagina saginoides (L.) H. Karst. 5 30 0.130 Lutz (2004)

Sagina saginoides (L.) H. Karst. 5 60 0.117 Lutz (2004)

Sagina saginoides (L.) H. Karst. 5 120 0.065 Lutz (2004)

Sagina saginoides (L.) H. Karst. 5 180 0.039 Lutz (2004)

Sagina saginoides (L.) H. Karst. 5 360 0.000 Lutz (2004)

Sagina saginoides (L.) H. Karst. 5 1440 0.000 Lutz (2004)

Salix hastata L. 1 0 1.000 Lutz (2004)

Salix hastata L. 1 1 0.138 Lutz (2004)

Salix hastata L. 1 5 0.069 Lutz (2004)

Salix hastata L. 1 10 0.057 Lutz (2004)

Salix hastata L. 1 30 0.046 Lutz (2004)

Salix hastata L. 1 60 0.046 Lutz (2004)

Salix hastata L. 1 120 0.000 Lutz (2004)

Salix hastata L. 1 180 0.000 Lutz (2004)

Salix hastata L. 1 360 0.000 Lutz (2004)

Salix hastata L. 1 1440 0.000 Lutz (2004)

Salix hastata L. 2 0 1.000 Lutz (2004)

Salix hastata L. 2 1 0.268 Lutz (2004)

Salix hastata L. 2 5 0.134 Lutz (2004)

Salix hastata L. 2 10 0.134 Lutz (2004)

Salix hastata L. 2 30 0.134 Lutz (2004)

Salix hastata L. 2 60 0.134 Lutz (2004)

Salix hastata L. 2 120 0.037 Lutz (2004)

Salix hastata L. 2 180 0.024 Lutz (2004)

Salix hastata L. 2 360 0.000 Lutz (2004)

Salix hastata L. 2 1440 0.000 Lutz (2004)

Salix hastata L. 3 0 1.000 Lutz (2004)

Salix hastata L. 3 1 0.209 Lutz (2004)

Salix hastata L. 3 5 0.110 Lutz (2004)

Salix hastata L. 3 10 0.099 Lutz (2004)

Salix hastata L. 3 30 0.066 Lutz (2004)

Salix hastata L. 3 60 0.066 Lutz (2004)

Salix hastata L. 3 120 0.055 Lutz (2004)

Salix hastata L. 3 180 0.022 Lutz (2004)

Salix hastata L. 3 360 0.000 Lutz (2004)

Salix hastata L. 3 1440 0.000 Lutz (2004)

Salix hastata L. 4 0 1.000 Lutz (2004)

Salix hastata L. 4 1 0.158 Lutz (2004)

Salix hastata L. 4 5 0.132 Lutz (2004)

Salix hastata L. 4 10 0.132 Lutz (2004)

Salix hastata L. 4 30 0.105 Lutz (2004)

Salix hastata L. 4 60 0.079 Lutz (2004)

Salix hastata L. 4 120 0.000 Lutz (2004)

Salix hastata L. 4 180 0.000 Lutz (2004)

Salix hastata L. 4 360 0.000 Lutz (2004)

Salix hastata L. 4 1440 0.000 Lutz (2004)

Salix hastata L. 5 0 1.000 Lutz (2004)

Salix hastata L. 5 1 0.111 Lutz (2004)

Salix hastata L. 5 5 0.074 Lutz (2004)

Salix hastata L. 5 10 0.074 Lutz (2004)

Salix hastata L. 5 30 0.074 Lutz (2004)

Salix hastata L. 5 60 0.074 Lutz (2004)

Salix hastata L. 5 120 0.037 Lutz (2004)

Salix hastata L. 5 180 0.000 Lutz (2004)

Salix hastata L. 5 360 0.000 Lutz (2004)

Salix hastata L. 5 1440 0.000 Lutz (2004)

Saxifraga bryoides L. 1 0 1.000 Lutz (2004)

Saxifraga bryoides L. 1 1 0.280 Lutz (2004)

Saxifraga bryoides L. 1 5 0.204 Lutz (2004)

Saxifraga bryoides L. 1 10 0.194 Lutz (2004)

Saxifraga bryoides L. 1 30 0.161 Lutz (2004)

Saxifraga bryoides L. 1 60 0.151 Lutz (2004)

Saxifraga bryoides L. 1 120 0.075 Lutz (2004)

Saxifraga bryoides L. 1 180 0.022 Lutz (2004)

Saxifraga bryoides L. 1 360 0.000 Lutz (2004)

Saxifraga bryoides L. 1 1440 0.000 Lutz (2004)

Saxifraga bryoides L. 2 0 1.000 Lutz (2004)

Saxifraga bryoides L. 2 1 0.484 Lutz (2004)

Saxifraga bryoides L. 2 5 0.374 Lutz (2004)

Saxifraga bryoides L. 2 10 0.363 Lutz (2004)

Saxifraga bryoides L. 2 30 0.330 Lutz (2004)

Saxifraga bryoides L. 2 60 0.319 Lutz (2004)

Saxifraga bryoides L. 2 120 0.198 Lutz (2004)

Saxifraga bryoides L. 2 180 0.154 Lutz (2004)

Saxifraga bryoides L. 2 360 0.000 Lutz (2004)

Saxifraga bryoides L. 2 1440 0.000 Lutz (2004)

Saxifraga bryoides L. 3 0 1.000 Lutz (2004)

Saxifraga bryoides L. 3 1 0.447 Lutz (2004)

Saxifraga bryoides L. 3 5 0.287 Lutz (2004)

Saxifraga bryoides L. 3 10 0.223 Lutz (2004)

Saxifraga bryoides L. 3 30 0.213 Lutz (2004)

Saxifraga bryoides L. 3 60 0.202 Lutz (2004)

Saxifraga bryoides L. 3 120 0.085 Lutz (2004)

Saxifraga bryoides L. 3 180 0.043 Lutz (2004)

Saxifraga bryoides L. 3 360 0.000 Lutz (2004)

Saxifraga bryoides L. 3 1440 0.000 Lutz (2004)

Saxifraga bryoides L. 4 0 1.000 Lutz (2004)

Saxifraga bryoides L. 4 1 0.237 Lutz (2004)

Saxifraga bryoides L. 4 5 0.175 Lutz (2004)

Saxifraga bryoides L. 4 10 0.155 Lutz (2004)

Saxifraga bryoides L. 4 30 0.124 Lutz (2004)

Saxifraga bryoides L. 4 60 0.124 Lutz (2004)

Saxifraga bryoides L. 4 120 0.052 Lutz (2004)

Saxifraga bryoides L. 4 180 0.031 Lutz (2004)

Saxifraga bryoides L. 4 360 0.021 Lutz (2004)

Saxifraga bryoides L. 4 1440 0.000 Lutz (2004)

Saxifraga bryoides L. 5 0 1.000 Lutz (2004)

Saxifraga bryoides L. 5 1 0.348 Lutz (2004)

Saxifraga bryoides L. 5 5 0.236 Lutz (2004)

Saxifraga bryoides L. 5 10 0.191 Lutz (2004)

Saxifraga bryoides L. 5 30 0.135 Lutz (2004)

Saxifraga bryoides L. 5 60 0.112 Lutz (2004)

Saxifraga bryoides L. 5 120 0.000 Lutz (2004)

Saxifraga bryoides L. 5 180 0.000 Lutz (2004)

Saxifraga bryoides L. 5 360 0.000 Lutz (2004)

Saxifraga bryoides L. 5 1440 0.000 Lutz (2004)

Saxifraga paniculata Mill. 1 0 1.000 Lutz (2004)

Saxifraga paniculata Mill. 1 1 0.362 Lutz (2004)

Saxifraga paniculata Mill. 1 5 0.277 Lutz (2004)

Saxifraga paniculata Mill. 1 10 0.266 Lutz (2004)

Saxifraga paniculata Mill. 1 30 0.213 Lutz (2004)

Saxifraga paniculata Mill. 1 60 0.202 Lutz (2004)

Saxifraga paniculata Mill. 1 120 0.064 Lutz (2004)

Saxifraga paniculata Mill. 1 180 0.011 Lutz (2004)

Saxifraga paniculata Mill. 1 360 0.000 Lutz (2004)

Saxifraga paniculata Mill. 1 1440 0.000 Lutz (2004)

Saxifraga paniculata Mill. 2 0 1.000 Lutz (2004)

Saxifraga paniculata Mill. 2 1 0.391 Lutz (2004)

Saxifraga paniculata Mill. 2 5 0.283 Lutz (2004)

Saxifraga paniculata Mill. 2 10 0.261 Lutz (2004)

Saxifraga paniculata Mill. 2 30 0.261 Lutz (2004)

Saxifraga paniculata Mill. 2 60 0.261 Lutz (2004)

Saxifraga paniculata Mill. 2 120 0.087 Lutz (2004)

Saxifraga paniculata Mill. 2 180 0.076 Lutz (2004)

Saxifraga paniculata Mill. 2 360 0.000 Lutz (2004)

Saxifraga paniculata Mill. 2 1440 0.000 Lutz (2004)

Saxifraga paniculata Mill. 3 0 1.000 Lutz (2004)

Saxifraga paniculata Mill. 3 1 0.344 Lutz (2004)

Saxifraga paniculata Mill. 3 5 0.215 Lutz (2004)

Saxifraga paniculata Mill. 3 10 0.183 Lutz (2004)

Saxifraga paniculata Mill. 3 30 0.151 Lutz (2004)

Saxifraga paniculata Mill. 3 60 0.140 Lutz (2004)

Saxifraga paniculata Mill. 3 120 0.054 Lutz (2004)

Saxifraga paniculata Mill. 3 180 0.011 Lutz (2004)

Saxifraga paniculata Mill. 3 360 0.011 Lutz (2004)

Saxifraga paniculata Mill. 3 1440 0.000 Lutz (2004)

Saxifraga paniculata Mill. 4 0 1.000 Lutz (2004)

Saxifraga paniculata Mill. 4 1 0.151 Lutz (2004)

Saxifraga paniculata Mill. 4 5 0.093 Lutz (2004)

Saxifraga paniculata Mill. 4 10 0.070 Lutz (2004)

Saxifraga paniculata Mill. 4 30 0.058 Lutz (2004)

Saxifraga paniculata Mill. 4 60 0.047 Lutz (2004)

Saxifraga paniculata Mill. 4 120 0.023 Lutz (2004)

Saxifraga paniculata Mill. 4 180 0.000 Lutz (2004)

Saxifraga paniculata Mill. 4 360 0.000 Lutz (2004)

Saxifraga paniculata Mill. 4 1440 0.000 Lutz (2004)

Saxifraga paniculata Mill. 5 0 1.000 Lutz (2004)

Saxifraga paniculata Mill. 5 1 0.350 Lutz (2004)

Saxifraga paniculata Mill. 5 5 0.250 Lutz (2004)

Saxifraga paniculata Mill. 5 10 0.213 Lutz (2004)

Saxifraga paniculata Mill. 5 30 0.163 Lutz (2004)

Saxifraga paniculata Mill. 5 60 0.150 Lutz (2004)

Saxifraga paniculata Mill. 5 120 0.050 Lutz (2004)

Saxifraga paniculata Mill. 5 180 0.000 Lutz (2004)

Saxifraga paniculata Mill. 5 360 0.000 Lutz (2004)

Saxifraga paniculata Mill. 5 1440 0.000 Lutz (2004)

Sclerochloa dura (L.) P. Beauv. 1 0 1.000 Pirzer (2007)

Sclerochloa dura (L.) P. Beauv. 1 1 0.763 Pirzer (2007)

Sclerochloa dura (L.) P. Beauv. 1 5 0.684 Pirzer (2007)

Sclerochloa dura (L.) P. Beauv. 1 10 0.632 Pirzer (2007)

Sclerochloa dura (L.) P. Beauv. 1 30 0.579 Pirzer (2007)

Sclerochloa dura (L.) P. Beauv. 1 60 0.553 Pirzer (2007)

Sclerochloa dura (L.) P. Beauv. 1 120 0.237 Pirzer (2007)

Sclerochloa dura (L.) P. Beauv. 1 180 0.000 Pirzer (2007)

Sclerochloa dura (L.) P. Beauv. 1 360 0.000 Pirzer (2007)

Sclerochloa dura (L.) P. Beauv. 1 1440 0.000 Pirzer (2007)

Sclerochloa dura (L.) P. Beauv. 2 0 1.000 Pirzer (2007)

Sclerochloa dura (L.) P. Beauv. 2 1 0.256 Pirzer (2007)

Sclerochloa dura (L.) P. Beauv. 2 5 0.256 Pirzer (2007)

Sclerochloa dura (L.) P. Beauv. 2 10 0.209 Pirzer (2007)

Sclerochloa dura (L.) P. Beauv. 2 30 0.116 Pirzer (2007)

Sclerochloa dura (L.) P. Beauv. 2 60 0.116 Pirzer (2007)

Sclerochloa dura (L.) P. Beauv. 2 120 0.093 Pirzer (2007)

Sclerochloa dura (L.) P. Beauv. 2 180 0.000 Pirzer (2007)

Sclerochloa dura (L.) P. Beauv. 2 360 0.000 Pirzer (2007)

Sclerochloa dura (L.) P. Beauv. 2 1440 0.000 Pirzer (2007)

Sclerochloa dura (L.) P. Beauv. 3 0 1.000 Pirzer (2007)

Sclerochloa dura (L.) P. Beauv. 3 1 0.840 Pirzer (2007)

Sclerochloa dura (L.) P. Beauv. 3 5 0.840 Pirzer (2007)

Sclerochloa dura (L.) P. Beauv. 3 10 0.840 Pirzer (2007)

Sclerochloa dura (L.) P. Beauv. 3 30 0.800 Pirzer (2007)

Sclerochloa dura (L.) P. Beauv. 3 60 0.800 Pirzer (2007)

Sclerochloa dura (L.) P. Beauv. 3 120 0.240 Pirzer (2007)

Sclerochloa dura (L.) P. Beauv. 3 180 0.040 Pirzer (2007)

Sclerochloa dura (L.) P. Beauv. 3 360 0.000 Pirzer (2007)

Sclerochloa dura (L.) P. Beauv. 3 1440 0.000 Pirzer (2007)

Sclerochloa dura (L.) P. Beauv. 4 0 1.000 Pirzer (2007)

Sclerochloa dura (L.) P. Beauv. 4 1 0.154 Pirzer (2007)

Sclerochloa dura (L.) P. Beauv. 4 5 0.077 Pirzer (2007)

Sclerochloa dura (L.) P. Beauv. 4 10 0.051 Pirzer (2007)

Sclerochloa dura (L.) P. Beauv. 4 30 0.026 Pirzer (2007)

Sclerochloa dura (L.) P. Beauv. 4 60 0.026 Pirzer (2007)

Sclerochloa dura (L.) P. Beauv. 4 120 0.026 Pirzer (2007)

Sclerochloa dura (L.) P. Beauv. 4 180 0.000 Pirzer (2007)

Sclerochloa dura (L.) P. Beauv. 4 360 0.000 Pirzer (2007)

Sclerochloa dura (L.) P. Beauv. 4 1440 0.000 Pirzer (2007)

Sclerochloa dura (L.) P. Beauv. 5 0 1.000 Pirzer (2007)

Sclerochloa dura (L.) P. Beauv. 5 1 0.905 Pirzer (2007)

Sclerochloa dura (L.) P. Beauv. 5 5 0.857 Pirzer (2007)

Sclerochloa dura (L.) P. Beauv. 5 10 0.857 Pirzer (2007)

Sclerochloa dura (L.) P. Beauv. 5 30 0.810 Pirzer (2007)

Sclerochloa dura (L.) P. Beauv. 5 60 0.810 Pirzer (2007)

Sclerochloa dura (L.) P. Beauv. 5 120 0.667 Pirzer (2007)

Sclerochloa dura (L.) P. Beauv. 5 180 0.381 Pirzer (2007)

Sclerochloa dura (L.) P. Beauv. 5 360 0.238 Pirzer (2007)

Sclerochloa dura (L.) P. Beauv. 5 1440 0.000 Pirzer (2007)

Sedum alpestre Vill. 1 0 1.000 Lutz (2004)

Sedum alpestre Vill. 1 1 0.315 Lutz (2004)

Sedum alpestre Vill. 1 5 0.239 Lutz (2004)

Sedum alpestre Vill. 1 10 0.239 Lutz (2004)

Sedum alpestre Vill. 1 30 0.228 Lutz (2004)

Sedum alpestre Vill. 1 60 0.228 Lutz (2004)

Sedum alpestre Vill. 1 120 0.152 Lutz (2004)

Sedum alpestre Vill. 1 180 0.054 Lutz (2004)

Sedum alpestre Vill. 1 360 0.000 Lutz (2004)

Sedum alpestre Vill. 1 1440 0.000 Lutz (2004)

Sedum alpestre Vill. 2 0 1.000 Lutz (2004)

Sedum alpestre Vill. 2 1 0.341 Lutz (2004)

Sedum alpestre Vill. 2 5 0.242 Lutz (2004)

Sedum alpestre Vill. 2 10 0.242 Lutz (2004)

Sedum alpestre Vill. 2 30 0.220 Lutz (2004)

Sedum alpestre Vill. 2 60 0.220 Lutz (2004)

Sedum alpestre Vill. 2 120 0.044 Lutz (2004)

Sedum alpestre Vill. 2 180 0.022 Lutz (2004)

Sedum alpestre Vill. 2 360 0.000 Lutz (2004)

Sedum alpestre Vill. 2 1440 0.000 Lutz (2004)

Sedum alpestre Vill. 3 0 1.000 Lutz (2004)

Sedum alpestre Vill. 3 1 0.176 Lutz (2004)

Sedum alpestre Vill. 3 5 0.110 Lutz (2004)

Sedum alpestre Vill. 3 10 0.110 Lutz (2004)

Sedum alpestre Vill. 3 30 0.088 Lutz (2004)

Sedum alpestre Vill. 3 60 0.066 Lutz (2004)

Sedum alpestre Vill. 3 120 0.022 Lutz (2004)

Sedum alpestre Vill. 3 180 0.022 Lutz (2004)

Sedum alpestre Vill. 3 360 0.022 Lutz (2004)

Sedum alpestre Vill. 3 1440 0.000 Lutz (2004)

Sedum alpestre Vill. 4 0 1.000 Lutz (2004)

Sedum alpestre Vill. 4 1 0.272 Lutz (2004)

Sedum alpestre Vill. 4 5 0.196 Lutz (2004)

Sedum alpestre Vill. 4 10 0.152 Lutz (2004)

Sedum alpestre Vill. 4 30 0.130 Lutz (2004)

Sedum alpestre Vill. 4 60 0.109 Lutz (2004)

Sedum alpestre Vill. 4 120 0.033 Lutz (2004)

Sedum alpestre Vill. 4 180 0.022 Lutz (2004)

Sedum alpestre Vill. 4 360 0.011 Lutz (2004)

Sedum alpestre Vill. 4 1440 0.000 Lutz (2004)

Sedum alpestre Vill. 5 0 1.000 Lutz (2004)

Sedum alpestre Vill. 5 1 0.292 Lutz (2004)

Sedum alpestre Vill. 5 5 0.191 Lutz (2004)

Sedum alpestre Vill. 5 10 0.169 Lutz (2004)

Sedum alpestre Vill. 5 30 0.157 Lutz (2004)

Sedum alpestre Vill. 5 60 0.124 Lutz (2004)

Sedum alpestre Vill. 5 120 0.056 Lutz (2004)

Sedum alpestre Vill. 5 180 0.011 Lutz (2004)

Sedum alpestre Vill. 5 360 0.000 Lutz (2004)

Sedum alpestre Vill. 5 1440 0.000 Lutz (2004)

Sempervivum montanum L. 1 0 1.000 Lutz (2004)

Sempervivum montanum L. 1 1 0.268 Lutz (2004)

Sempervivum montanum L. 1 5 0.227 Lutz (2004)

Sempervivum montanum L. 1 10 0.216 Lutz (2004)

Sempervivum montanum L. 1 30 0.186 Lutz (2004)

Sempervivum montanum L. 1 60 0.186 Lutz (2004)

Sempervivum montanum L. 1 120 0.103 Lutz (2004)

Sempervivum montanum L. 1 180 0.082 Lutz (2004)

Sempervivum montanum L. 1 360 0.031 Lutz (2004)

Sempervivum montanum L. 1 1440 0.000 Lutz (2004)

Sempervivum montanum L. 2 0 1.000 Lutz (2004)

Sempervivum montanum L. 2 1 0.483 Lutz (2004)

Sempervivum montanum L. 2 5 0.448 Lutz (2004)

Sempervivum montanum L. 2 10 0.414 Lutz (2004)

Sempervivum montanum L. 2 30 0.345 Lutz (2004)

Sempervivum montanum L. 2 60 0.333 Lutz (2004)

Sempervivum montanum L. 2 120 0.149 Lutz (2004)

Sempervivum montanum L. 2 180 0.069 Lutz (2004)

Sempervivum montanum L. 2 360 0.000 Lutz (2004)

Sempervivum montanum L. 2 1440 0.000 Lutz (2004)

Sempervivum montanum L. 3 0 1.000 Lutz (2004)

Sempervivum montanum L. 3 1 0.413 Lutz (2004)

Sempervivum montanum L. 3 5 0.337 Lutz (2004)

Sempervivum montanum L. 3 10 0.272 Lutz (2004)

Sempervivum montanum L. 3 30 0.239 Lutz (2004)

Sempervivum montanum L. 3 60 0.239 Lutz (2004)

Sempervivum montanum L. 3 120 0.130 Lutz (2004)

Sempervivum montanum L. 3 180 0.033 Lutz (2004)

Sempervivum montanum L. 3 360 0.000 Lutz (2004)

Sempervivum montanum L. 3 1440 0.000 Lutz (2004)

Sempervivum montanum L. 4 0 1.000 Lutz (2004)

Sempervivum montanum L. 4 1 0.378 Lutz (2004)

Sempervivum montanum L. 4 5 0.296 Lutz (2004)

Sempervivum montanum L. 4 10 0.276 Lutz (2004)

Sempervivum montanum L. 4 30 0.245 Lutz (2004)

Sempervivum montanum L. 4 60 0.133 Lutz (2004)

Sempervivum montanum L. 4 120 0.031 Lutz (2004)

Sempervivum montanum L. 4 180 0.010 Lutz (2004)

Sempervivum montanum L. 4 360 0.000 Lutz (2004)

Sempervivum montanum L. 4 1440 0.000 Lutz (2004)

Sempervivum montanum L. 5 0 1.000 Lutz (2004)

Sempervivum montanum L. 5 1 0.176 Lutz (2004)

Sempervivum montanum L. 5 5 0.165 Lutz (2004)

Sempervivum montanum L. 5 10 0.154 Lutz (2004)

Sempervivum montanum L. 5 30 0.143 Lutz (2004)

Sempervivum montanum L. 5 60 0.132 Lutz (2004)

Sempervivum montanum L. 5 120 0.033 Lutz (2004)

Sempervivum montanum L. 5 180 0.011 Lutz (2004)

Sempervivum montanum L. 5 360 0.000 Lutz (2004)

Sempervivum montanum L. 5 1440 0.000 Lutz (2004)

Setaria viridis (L.) P. Beauv. 1 0 1.000 Pirzer (2007)

Setaria viridis (L.) P. Beauv. 1 1 0.614 Pirzer (2007)

Setaria viridis (L.) P. Beauv. 1 5 0.591 Pirzer (2007)

Setaria viridis (L.) P. Beauv. 1 10 0.591 Pirzer (2007)

Setaria viridis (L.) P. Beauv. 1 30 0.523 Pirzer (2007)

Setaria viridis (L.) P. Beauv. 1 60 0.500 Pirzer (2007)

Setaria viridis (L.) P. Beauv. 1 120 0.182 Pirzer (2007)

Setaria viridis (L.) P. Beauv. 1 180 0.159 Pirzer (2007)

Setaria viridis (L.) P. Beauv. 1 360 0.023 Pirzer (2007)

Setaria viridis (L.) P. Beauv. 1 1440 0.000 Pirzer (2007)

Setaria viridis (L.) P. Beauv. 2 0 1.000 Pirzer (2007)

Setaria viridis (L.) P. Beauv. 2 1 0.567 Pirzer (2007)

Setaria viridis (L.) P. Beauv. 2 5 0.367 Pirzer (2007)

Setaria viridis (L.) P. Beauv. 2 10 0.367 Pirzer (2007)

Setaria viridis (L.) P. Beauv. 2 30 0.367 Pirzer (2007)

Setaria viridis (L.) P. Beauv. 2 60 0.367 Pirzer (2007)

Setaria viridis (L.) P. Beauv. 2 120 0.133 Pirzer (2007)

Setaria viridis (L.) P. Beauv. 2 180 0.033 Pirzer (2007)

Setaria viridis (L.) P. Beauv. 2 360 0.000 Pirzer (2007)

Setaria viridis (L.) P. Beauv. 2 1440 0.000 Pirzer (2007)

Setaria viridis (L.) P. Beauv. 3 0 1.000 Pirzer (2007)

Setaria viridis (L.) P. Beauv. 3 1 0.000 Pirzer (2007)

Setaria viridis (L.) P. Beauv. 3 5 0.000 Pirzer (2007)

Setaria viridis (L.) P. Beauv. 3 10 0.000 Pirzer (2007)

Setaria viridis (L.) P. Beauv. 3 30 0.000 Pirzer (2007)

Setaria viridis (L.) P. Beauv. 3 60 0.000 Pirzer (2007)

Setaria viridis (L.) P. Beauv. 3 120 0.000 Pirzer (2007)

Setaria viridis (L.) P. Beauv. 3 180 0.000 Pirzer (2007)

Setaria viridis (L.) P. Beauv. 3 360 0.000 Pirzer (2007)

Setaria viridis (L.) P. Beauv. 3 1440 0.000 Pirzer (2007)

Setaria viridis (L.) P. Beauv. 4 0 1.000 Pirzer (2007)

Setaria viridis (L.) P. Beauv. 4 1 0.897 Pirzer (2007)

Setaria viridis (L.) P. Beauv. 4 5 0.897 Pirzer (2007)

Setaria viridis (L.) P. Beauv. 4 10 0.897 Pirzer (2007)

Setaria viridis (L.) P. Beauv. 4 30 0.828 Pirzer (2007)

Setaria viridis (L.) P. Beauv. 4 60 0.793 Pirzer (2007)

Setaria viridis (L.) P. Beauv. 4 120 0.138 Pirzer (2007)

Setaria viridis (L.) P. Beauv. 4 180 0.069 Pirzer (2007)

Setaria viridis (L.) P. Beauv. 4 360 0.000 Pirzer (2007)

Setaria viridis (L.) P. Beauv. 4 1440 0.000 Pirzer (2007)

Setaria viridis (L.) P. Beauv. 5 0 1.000 Pirzer (2007)

Setaria viridis (L.) P. Beauv. 5 1 0.235 Pirzer (2007)

Setaria viridis (L.) P. Beauv. 5 5 0.235 Pirzer (2007)

Setaria viridis (L.) P. Beauv. 5 10 0.235 Pirzer (2007)

Setaria viridis (L.) P. Beauv. 5 30 0.235 Pirzer (2007)

Setaria viridis (L.) P. Beauv. 5 60 0.235 Pirzer (2007)

Setaria viridis (L.) P. Beauv. 5 120 0.059 Pirzer (2007)

Setaria viridis (L.) P. Beauv. 5 180 0.059 Pirzer (2007)

Setaria viridis (L.) P. Beauv. 5 360 0.000 Pirzer (2007)

Setaria viridis (L.) P. Beauv. 5 1440 0.000 Pirzer (2007)

Silene exscapa All. 1 0 1.000 Lutz (2004)

Silene exscapa All. 1 1 0.197 Lutz (2004)

Silene exscapa All. 1 5 0.070 Lutz (2004)

Silene exscapa All. 1 10 0.070 Lutz (2004)

Silene exscapa All. 1 30 0.056 Lutz (2004)

Silene exscapa All. 1 60 0.056 Lutz (2004)

Silene exscapa All. 1 120 0.014 Lutz (2004)

Silene exscapa All. 1 180 0.000 Lutz (2004)

Silene exscapa All. 1 360 0.000 Lutz (2004)

Silene exscapa All. 1 1440 0.000 Lutz (2004)

Silene exscapa All. 2 0 1.000 Lutz (2004)

Silene exscapa All. 2 1 0.348 Lutz (2004)

Silene exscapa All. 2 5 0.247 Lutz (2004)

Silene exscapa All. 2 10 0.202 Lutz (2004)

Silene exscapa All. 2 30 0.180 Lutz (2004)

Silene exscapa All. 2 60 0.146 Lutz (2004)

Silene exscapa All. 2 120 0.056 Lutz (2004)

Silene exscapa All. 2 180 0.045 Lutz (2004)

Silene exscapa All. 2 360 0.034 Lutz (2004)

Silene exscapa All. 2 1440 0.000 Lutz (2004)

Silene exscapa All. 3 0 1.000 Lutz (2004)

Silene exscapa All. 3 1 0.070 Lutz (2004)

Silene exscapa All. 3 5 0.035 Lutz (2004)

Silene exscapa All. 3 10 0.023 Lutz (2004)

Silene exscapa All. 3 30 0.023 Lutz (2004)

Silene exscapa All. 3 60 0.023 Lutz (2004)

Silene exscapa All. 3 120 0.012 Lutz (2004)

Silene exscapa All. 3 180 0.012 Lutz (2004)

Silene exscapa All. 3 360 0.000 Lutz (2004)

Silene exscapa All. 3 1440 0.000 Lutz (2004)

Silene exscapa All. 4 0 1.000 Lutz (2004)

Silene exscapa All. 4 1 0.056 Lutz (2004)

Silene exscapa All. 4 5 0.000 Lutz (2004)

Silene exscapa All. 4 10 0.000 Lutz (2004)

Silene exscapa All. 4 30 0.000 Lutz (2004)

Silene exscapa All. 4 60 0.000 Lutz (2004)

Silene exscapa All. 4 120 0.000 Lutz (2004)

Silene exscapa All. 4 180 0.000 Lutz (2004)

Silene exscapa All. 4 360 0.000 Lutz (2004)

Silene exscapa All. 4 1440 0.000 Lutz (2004)

Silene exscapa All. 5 0 1.000 Lutz (2004)

Silene exscapa All. 5 1 0.205 Lutz (2004)

Silene exscapa All. 5 5 0.141 Lutz (2004)

Silene exscapa All. 5 10 0.115 Lutz (2004)

Silene exscapa All. 5 30 0.064 Lutz (2004)

Silene exscapa All. 5 60 0.038 Lutz (2004)

Silene exscapa All. 5 120 0.026 Lutz (2004)

Silene exscapa All. 5 180 0.013 Lutz (2004)

Silene exscapa All. 5 360 0.000 Lutz (2004)

Silene exscapa All. 5 1440 0.000 Lutz (2004)

Soldanella pusilla Baumg. 1 0 1.000 Lutz (2004)

Soldanella pusilla Baumg. 1 1 0.211 Lutz (2004)

Soldanella pusilla Baumg. 1 5 0.171 Lutz (2004)

Soldanella pusilla Baumg. 1 10 0.145 Lutz (2004)

Soldanella pusilla Baumg. 1 30 0.145 Lutz (2004)

Soldanella pusilla Baumg. 1 60 0.145 Lutz (2004)

Soldanella pusilla Baumg. 1 120 0.066 Lutz (2004)

Soldanella pusilla Baumg. 1 180 0.026 Lutz (2004)

Soldanella pusilla Baumg. 1 360 0.000 Lutz (2004)

Soldanella pusilla Baumg. 1 1440 0.000 Lutz (2004)

Soldanella pusilla Baumg. 2 0 1.000 Lutz (2004)

Soldanella pusilla Baumg. 2 1 0.370 Lutz (2004)

Soldanella pusilla Baumg. 2 5 0.284 Lutz (2004)

Soldanella pusilla Baumg. 2 10 0.259 Lutz (2004)

Soldanella pusilla Baumg. 2 30 0.247 Lutz (2004)

Soldanella pusilla Baumg. 2 60 0.247 Lutz (2004)

Soldanella pusilla Baumg. 2 120 0.136 Lutz (2004)

Soldanella pusilla Baumg. 2 180 0.099 Lutz (2004)

Soldanella pusilla Baumg. 2 360 0.049 Lutz (2004)

Soldanella pusilla Baumg. 2 1440 0.000 Lutz (2004)

Soldanella pusilla Baumg. 3 0 1.000 Lutz (2004)

Soldanella pusilla Baumg. 3 1 0.224 Lutz (2004)

Soldanella pusilla Baumg. 3 5 0.122 Lutz (2004)

Soldanella pusilla Baumg. 3 10 0.122 Lutz (2004)

Soldanella pusilla Baumg. 3 30 0.122 Lutz (2004)

Soldanella pusilla Baumg. 3 60 0.112 Lutz (2004)

Soldanella pusilla Baumg. 3 120 0.051 Lutz (2004)

Soldanella pusilla Baumg. 3 180 0.000 Lutz (2004)

Soldanella pusilla Baumg. 3 360 0.000 Lutz (2004)

Soldanella pusilla Baumg. 3 1440 0.000 Lutz (2004)

Soldanella pusilla Baumg. 4 0 1.000 Lutz (2004)

Soldanella pusilla Baumg. 4 1 0.114 Lutz (2004)

Soldanella pusilla Baumg. 4 5 0.076 Lutz (2004)

Soldanella pusilla Baumg. 4 10 0.051 Lutz (2004)

Soldanella pusilla Baumg. 4 30 0.051 Lutz (2004)

Soldanella pusilla Baumg. 4 60 0.051 Lutz (2004)

Soldanella pusilla Baumg. 4 120 0.000 Lutz (2004)

Soldanella pusilla Baumg. 4 180 0.000 Lutz (2004)

Soldanella pusilla Baumg. 4 360 0.000 Lutz (2004)

Soldanella pusilla Baumg. 4 1440 0.000 Lutz (2004)

Soldanella pusilla Baumg. 5 0 1.000 Lutz (2004)

Soldanella pusilla Baumg. 5 1 0.250 Lutz (2004)

Soldanella pusilla Baumg. 5 5 0.200 Lutz (2004)

Soldanella pusilla Baumg. 5 10 0.175 Lutz (2004)

Soldanella pusilla Baumg. 5 30 0.125 Lutz (2004)

Soldanella pusilla Baumg. 5 60 0.113 Lutz (2004)

Soldanella pusilla Baumg. 5 120 0.075 Lutz (2004)

Soldanella pusilla Baumg. 5 180 0.012 Lutz (2004)

Soldanella pusilla Baumg. 5 360 0.012 Lutz (2004)

Soldanella pusilla Baumg. 5 1440 0.000 Lutz (2004)

Solidago virgaurea ssp. minuta (L.) Arcang. 1 0 1.000 Lutz (2004)

Solidago virgaurea ssp. minuta (L.) Arcang. 1 1 0.612 Lutz (2004)

Solidago virgaurea ssp. minuta (L.) Arcang. 1 5 0.469 Lutz (2004)

Solidago virgaurea ssp. minuta (L.) Arcang. 1 10 0.449 Lutz (2004)

Solidago virgaurea ssp. minuta (L.) Arcang. 1 30 0.388 Lutz (2004)

Solidago virgaurea ssp. minuta (L.) Arcang. 1 60 0.388 Lutz (2004)

Solidago virgaurea ssp. minuta (L.) Arcang. 1 120 0.163 Lutz (2004)

Solidago virgaurea ssp. minuta (L.) Arcang. 1 180 0.082 Lutz (2004)

Solidago virgaurea ssp. minuta (L.) Arcang. 1 360 0.000 Lutz (2004)

Solidago virgaurea ssp. minuta (L.) Arcang. 1 1440 0.000 Lutz (2004)

Solidago virgaurea ssp. minuta (L.) Arcang. 2 0 1.000 Lutz (2004)

Solidago virgaurea ssp. minuta (L.) Arcang. 2 1 0.510 Lutz (2004)

Solidago virgaurea ssp. minuta (L.) Arcang. 2 5 0.469 Lutz (2004)

Solidago virgaurea ssp. minuta (L.) Arcang. 2 10 0.469 Lutz (2004)

Solidago virgaurea ssp. minuta (L.) Arcang. 2 30 0.469 Lutz (2004)

Solidago virgaurea ssp. minuta (L.) Arcang. 2 60 0.429 Lutz (2004)

Solidago virgaurea ssp. minuta (L.) Arcang. 2 120 0.143 Lutz (2004)

Solidago virgaurea ssp. minuta (L.) Arcang. 2 180 0.102 Lutz (2004)

Solidago virgaurea ssp. minuta (L.) Arcang. 2 360 0.000 Lutz (2004)

Solidago virgaurea ssp. minuta (L.) Arcang. 2 1440 0.000 Lutz (2004)

Solidago virgaurea ssp. minuta (L.) Arcang. 3 0 1.000 Lutz (2004)

Solidago virgaurea ssp. minuta (L.) Arcang. 3 1 0.360 Lutz (2004)

Solidago virgaurea ssp. minuta (L.) Arcang. 3 5 0.200 Lutz (2004)

Solidago virgaurea ssp. minuta (L.) Arcang. 3 10 0.180 Lutz (2004)

Solidago virgaurea ssp. minuta (L.) Arcang. 3 30 0.160 Lutz (2004)

Solidago virgaurea ssp. minuta (L.) Arcang. 3 60 0.160 Lutz (2004)

Solidago virgaurea ssp. minuta (L.) Arcang. 3 120 0.060 Lutz (2004)

Solidago virgaurea ssp. minuta (L.) Arcang. 3 180 0.020 Lutz (2004)

Solidago virgaurea ssp. minuta (L.) Arcang. 3 360 0.000 Lutz (2004)

Solidago virgaurea ssp. minuta (L.) Arcang. 3 1440 0.000 Lutz (2004)

Solidago virgaurea ssp. minuta (L.) Arcang. 4 0 1.000 Lutz (2004)

Solidago virgaurea ssp. minuta (L.) Arcang. 4 1 0.286 Lutz (2004)

Solidago virgaurea ssp. minuta (L.) Arcang. 4 5 0.122 Lutz (2004)

Solidago virgaurea ssp. minuta (L.) Arcang. 4 10 0.102 Lutz (2004)

Solidago virgaurea ssp. minuta (L.) Arcang. 4 30 0.061 Lutz (2004)

Solidago virgaurea ssp. minuta (L.) Arcang. 4 60 0.061 Lutz (2004)

Solidago virgaurea ssp. minuta (L.) Arcang. 4 120 0.041 Lutz (2004)

Solidago virgaurea ssp. minuta (L.) Arcang. 4 180 0.020 Lutz (2004)

Solidago virgaurea ssp. minuta (L.) Arcang. 4 360 0.020 Lutz (2004)

Solidago virgaurea ssp. minuta (L.) Arcang. 4 1440 0.000 Lutz (2004)

Solidago virgaurea ssp. minuta (L.) Arcang. 5 0 1.000 Lutz (2004)

Solidago virgaurea ssp. minuta (L.) Arcang. 5 1 0.160 Lutz (2004)

Solidago virgaurea ssp. minuta (L.) Arcang. 5 5 0.080 Lutz (2004)

Solidago virgaurea ssp. minuta (L.) Arcang. 5 10 0.080 Lutz (2004)

Solidago virgaurea ssp. minuta (L.) Arcang. 5 30 0.080 Lutz (2004)

Solidago virgaurea ssp. minuta (L.) Arcang. 5 60 0.080 Lutz (2004)

Solidago virgaurea ssp. minuta (L.) Arcang. 5 120 0.040 Lutz (2004)

Solidago virgaurea ssp. minuta (L.) Arcang. 5 180 0.020 Lutz (2004)

Solidago virgaurea ssp. minuta (L.) Arcang. 5 360 0.000 Lutz (2004)

Solidago virgaurea ssp. minuta (L.) Arcang. 5 1440 0.000 Lutz (2004)

Sorghum halepense (L.) Pers. 1 0 1.000 Pirzer (2007)

Sorghum halepense (L.) Pers. 1 1 0.429 Pirzer (2007)

Sorghum halepense (L.) Pers. 1 5 0.429 Pirzer (2007)

Sorghum halepense (L.) Pers. 1 10 0.429 Pirzer (2007)

Sorghum halepense (L.) Pers. 1 30 0.429 Pirzer (2007)

Sorghum halepense (L.) Pers. 1 60 0.286 Pirzer (2007)

Sorghum halepense (L.) Pers. 1 120 0.000 Pirzer (2007)

Sorghum halepense (L.) Pers. 1 180 0.000 Pirzer (2007)

Sorghum halepense (L.) Pers. 1 360 0.000 Pirzer (2007)

Sorghum halepense (L.) Pers. 1 1440 0.000 Pirzer (2007)

Sorghum halepense (L.) Pers. 2 0 1.000 Pirzer (2007)

Sorghum halepense (L.) Pers. 2 1 0.500 Pirzer (2007)

Sorghum halepense (L.) Pers. 2 5 0.500 Pirzer (2007)

Sorghum halepense (L.) Pers. 2 10 0.500 Pirzer (2007)

Sorghum halepense (L.) Pers. 2 30 0.000 Pirzer (2007)

Sorghum halepense (L.) Pers. 2 60 0.000 Pirzer (2007)

Sorghum halepense (L.) Pers. 2 120 0.000 Pirzer (2007)

Sorghum halepense (L.) Pers. 2 180 0.000 Pirzer (2007)

Sorghum halepense (L.) Pers. 2 360 0.000 Pirzer (2007)

Sorghum halepense (L.) Pers. 2 1440 0.000 Pirzer (2007)

Sorghum halepense (L.) Pers. 3 0 1.000 Pirzer (2007)

Sorghum halepense (L.) Pers. 3 1 0.455 Pirzer (2007)

Sorghum halepense (L.) Pers. 3 5 0.364 Pirzer (2007)

Sorghum halepense (L.) Pers. 3 10 0.182 Pirzer (2007)

Sorghum halepense (L.) Pers. 3 30 0.182 Pirzer (2007)

Sorghum halepense (L.) Pers. 3 60 0.182 Pirzer (2007)

Sorghum halepense (L.) Pers. 3 120 0.182 Pirzer (2007)

Sorghum halepense (L.) Pers. 3 180 0.182 Pirzer (2007)

Sorghum halepense (L.) Pers. 3 360 0.000 Pirzer (2007)

Sorghum halepense (L.) Pers. 3 1440 0.000 Pirzer (2007)

Sorghum halepense (L.) Pers. 4 0 1.000 Pirzer (2007)

Sorghum halepense (L.) Pers. 4 1 0.227 Pirzer (2007)

Sorghum halepense (L.) Pers. 4 5 0.045 Pirzer (2007)

Sorghum halepense (L.) Pers. 4 10 0.000 Pirzer (2007)

Sorghum halepense (L.) Pers. 4 30 0.000 Pirzer (2007)

Sorghum halepense (L.) Pers. 4 60 0.000 Pirzer (2007)

Sorghum halepense (L.) Pers. 4 120 0.000 Pirzer (2007)

Sorghum halepense (L.) Pers. 4 180 0.000 Pirzer (2007)

Sorghum halepense (L.) Pers. 4 360 0.000 Pirzer (2007)

Sorghum halepense (L.) Pers. 4 1440 0.000 Pirzer (2007)

Sorghum halepense (L.) Pers. 5 0 1.000 Pirzer (2007)

Sorghum halepense (L.) Pers. 5 1 1.000 Pirzer (2007)

Sorghum halepense (L.) Pers. 5 5 0.900 Pirzer (2007)

Sorghum halepense (L.) Pers. 5 10 0.800 Pirzer (2007)

Sorghum halepense (L.) Pers. 5 30 0.700 Pirzer (2007)

Sorghum halepense (L.) Pers. 5 60 0.600 Pirzer (2007)

Sorghum halepense (L.) Pers. 5 120 0.000 Pirzer (2007)

Sorghum halepense (L.) Pers. 5 180 0.000 Pirzer (2007)

Sorghum halepense (L.) Pers. 5 360 0.000 Pirzer (2007)

Sorghum halepense (L.) Pers. 5 1440 0.000 Pirzer (2007)

Stellaria media agg. 1 0 1.000 original measurement

Stellaria media agg. 1 1 0.066 original measurement

Stellaria media agg. 1 5 0.049 original measurement

Stellaria media agg. 1 10 0.049 original measurement

Stellaria media agg. 1 30 0.049 original measurement

Stellaria media agg. 1 60 0.033 original measurement

Stellaria media agg. 1 120 0.033 original measurement

Stellaria media agg. 1 180 0.033 original measurement

Stellaria media agg. 1 360 0.000 original measurement

Stellaria media agg. 1 1440 0.000 original measurement

Stellaria media agg. 2 0 1.000 original measurement

Stellaria media agg. 2 1 0.082 original measurement

Stellaria media agg. 2 5 0.066 original measurement

Stellaria media agg. 2 10 0.033 original measurement

Stellaria media agg. 2 30 0.033 original measurement

Stellaria media agg. 2 60 0.033 original measurement

Stellaria media agg. 2 120 0.016 original measurement

Stellaria media agg. 2 180 0.000 original measurement

Stellaria media agg. 2 360 0.000 original measurement

Stellaria media agg. 2 1440 0.000 original measurement

Stellaria media agg. 3 0 1.000 original measurement

Stellaria media agg. 3 1 0.190 original measurement

Stellaria media agg. 3 5 0.143 original measurement

Stellaria media agg. 3 10 0.143 original measurement

Stellaria media agg. 3 30 0.071 original measurement

Stellaria media agg. 3 60 0.071 original measurement

Stellaria media agg. 3 120 0.071 original measurement

Stellaria media agg. 3 180 0.000 original measurement

Stellaria media agg. 3 360 0.000 original measurement

Stellaria media agg. 3 1440 0.000 original measurement

Stellaria media agg. 4 0 1.000 original measurement

Stellaria media agg. 4 1 0.123 original measurement

Stellaria media agg. 4 5 0.108 original measurement

Stellaria media agg. 4 10 0.092 original measurement

Stellaria media agg. 4 30 0.077 original measurement

Stellaria media agg. 4 60 0.062 original measurement

Stellaria media agg. 4 120 0.062 original measurement

Stellaria media agg. 4 180 0.031 original measurement

Stellaria media agg. 4 360 0.015 original measurement

Stellaria media agg. 4 1440 0.000 original measurement

Thymus pulegioides s.l. L. 1 0 1.000 original measurement

Thymus pulegioides s.l. L. 1 1 0.268 original measurement

Thymus pulegioides s.l. L. 1 5 0.141 original measurement

Thymus pulegioides s.l. L. 1 10 0.099 original measurement

Thymus pulegioides s.l. L. 1 30 0.085 original measurement

Thymus pulegioides s.l. L. 1 60 0.070 original measurement

Thymus pulegioides s.l. L. 1 120 0.000 original measurement

Thymus pulegioides s.l. L. 1 180 0.000 original measurement

Thymus pulegioides s.l. L. 2 0 1.000 original measurement

Thymus pulegioides s.l. L. 2 1 0.065 original measurement

Thymus pulegioides s.l. L. 2 5 0.052 original measurement

Thymus pulegioides s.l. L. 2 10 0.052 original measurement

Thymus pulegioides s.l. L. 2 30 0.026 original measurement

Thymus pulegioides s.l. L. 2 60 0.026 original measurement

Thymus pulegioides s.l. L. 2 120 0.013 original measurement

Thymus pulegioides s.l. L. 2 180 0.013 original measurement

Thymus pulegioides s.l. L. 2 360 0.000 original measurement

Thymus pulegioides s.l. L. 2 1440 0.000 original measurement

Thymus pulegioides s.l. L. 3 0 1.000 original measurement

Thymus pulegioides s.l. L. 3 1 0.299 original measurement

Thymus pulegioides s.l. L. 3 5 0.195 original measurement

Thymus pulegioides s.l. L. 3 10 0.182 original measurement

Thymus pulegioides s.l. L. 3 30 0.143 original measurement

Thymus pulegioides s.l. L. 3 60 0.130 original measurement

Thymus pulegioides s.l. L. 3 120 0.078 original measurement

Thymus pulegioides s.l. L. 3 180 0.039 original measurement

Thymus pulegioides s.l. L. 3 360 0.013 original measurement

Thymus pulegioides s.l. L. 3 1440 0.000 original measurement

Thymus pulegioides s.l. L. 4 0 1.000 original measurement

Thymus pulegioides s.l. L. 4 1 0.256 original measurement

Thymus pulegioides s.l. L. 4 5 0.207 original measurement

Thymus pulegioides s.l. L. 4 10 0.195 original measurement

Thymus pulegioides s.l. L. 4 30 0.183 original measurement

Thymus pulegioides s.l. L. 4 60 0.183 original measurement

Thymus pulegioides s.l. L. 4 120 0.061 original measurement

Thymus pulegioides s.l. L. 4 180 0.024 original measurement

Thymus pulegioides s.l. L. 4 360 0.000 original measurement

Thymus pulegioides s.l. L. 4 1440 0.000 original measurement

Trifolium badium Schreb. 1 0 1.000 Lutz (2004)

Trifolium badium Schreb. 1 1 0.041 Lutz (2004)

Trifolium badium Schreb. 1 5 0.027 Lutz (2004)

Trifolium badium Schreb. 1 10 0.027 Lutz (2004)

Trifolium badium Schreb. 1 30 0.000 Lutz (2004)

Trifolium badium Schreb. 1 60 0.000 Lutz (2004)

Trifolium badium Schreb. 1 120 0.000 Lutz (2004)

Trifolium badium Schreb. 1 180 0.000 Lutz (2004)

Trifolium badium Schreb. 1 360 0.000 Lutz (2004)

Trifolium badium Schreb. 1 1440 0.000 Lutz (2004)

Trifolium badium Schreb. 2 0 1.000 Lutz (2004)

Trifolium badium Schreb. 2 1 0.089 Lutz (2004)

Trifolium badium Schreb. 2 5 0.051 Lutz (2004)

Trifolium badium Schreb. 2 10 0.051 Lutz (2004)

Trifolium badium Schreb. 2 30 0.051 Lutz (2004)

Trifolium badium Schreb. 2 60 0.051 Lutz (2004)

Trifolium badium Schreb. 2 120 0.013 Lutz (2004)

Trifolium badium Schreb. 2 180 0.000 Lutz (2004)

Trifolium badium Schreb. 2 360 0.000 Lutz (2004)

Trifolium badium Schreb. 2 1440 0.000 Lutz (2004)

Trifolium badium Schreb. 3 0 1.000 Lutz (2004)

Trifolium badium Schreb. 3 1 0.037 Lutz (2004)

Trifolium badium Schreb. 3 5 0.012 Lutz (2004)

Trifolium badium Schreb. 3 10 0.012 Lutz (2004)

Trifolium badium Schreb. 3 30 0.012 Lutz (2004)

Trifolium badium Schreb. 3 60 0.012 Lutz (2004)

Trifolium badium Schreb. 3 120 0.000 Lutz (2004)

Trifolium badium Schreb. 3 180 0.000 Lutz (2004)

Trifolium badium Schreb. 3 360 0.000 Lutz (2004)

Trifolium badium Schreb. 3 1440 0.000 Lutz (2004)

Trifolium badium Schreb. 4 0 1.000 Lutz (2004)

Trifolium badium Schreb. 4 1 0.000 Lutz (2004)

Trifolium badium Schreb. 4 5 0.000 Lutz (2004)

Trifolium badium Schreb. 4 10 0.000 Lutz (2004)

Trifolium badium Schreb. 4 30 0.000 Lutz (2004)

Trifolium badium Schreb. 4 60 0.000 Lutz (2004)

Trifolium badium Schreb. 4 120 0.000 Lutz (2004)

Trifolium badium Schreb. 4 180 0.000 Lutz (2004)

Trifolium badium Schreb. 4 360 0.000 Lutz (2004)

Trifolium badium Schreb. 4 1440 0.000 Lutz (2004)

Trifolium badium Schreb. 5 0 1.000 Lutz (2004)

Trifolium badium Schreb. 5 1 0.056 Lutz (2004)

Trifolium badium Schreb. 5 5 0.019 Lutz (2004)

Trifolium badium Schreb. 5 10 0.019 Lutz (2004)

Trifolium badium Schreb. 5 30 0.000 Lutz (2004)

Trifolium badium Schreb. 5 60 0.000 Lutz (2004)

Trifolium badium Schreb. 5 120 0.000 Lutz (2004)

Trifolium badium Schreb. 5 180 0.000 Lutz (2004)

Trifolium badium Schreb. 5 360 0.000 Lutz (2004)

Trifolium badium Schreb. 5 1440 0.000 Lutz (2004)

Trifolium repens L. 1 0 1.000 original measurement

Trifolium repens L. 1 1 0.038 original measurement

Trifolium repens L. 1 5 0.019 original measurement

Trifolium repens L. 1 10 0.019 original measurement

Trifolium repens L. 1 30 0.019 original measurement

Trifolium repens L. 1 60 0.019 original measurement

Trifolium repens L. 1 120 0.019 original measurement

Trifolium repens L. 1 180 0.019 original measurement

Trifolium repens L. 1 360 0.000 original measurement

Trifolium repens L. 1 1440 0.000 original measurement

Trifolium repens L. 2 0 1.000 original measurement

Trifolium repens L. 2 1 0.043 original measurement

Trifolium repens L. 2 5 0.014 original measurement

Trifolium repens L. 2 10 0.014 original measurement

Trifolium repens L. 2 30 0.014 original measurement

Trifolium repens L. 2 60 0.000 original measurement

Trifolium repens L. 2 120 0.000 original measurement

Trifolium repens L. 2 180 0.000 original measurement

Trifolium repens L. 2 360 0.000 original measurement

Trifolium repens L. 2 1440 0.000 original measurement

Trifolium repens L. 3 0 1.000 original measurement

Trifolium repens L. 3 1 0.058 original measurement

Trifolium repens L. 3 5 0.058 original measurement

Trifolium repens L. 3 10 0.038 original measurement

Trifolium repens L. 3 30 0.038 original measurement

Trifolium repens L. 3 60 0.038 original measurement

Trifolium repens L. 3 120 0.038 original measurement

Trifolium repens L. 3 180 0.019 original measurement

Trifolium repens L. 3 360 0.000 original measurement

Trifolium repens L. 3 1440 0.000 original measurement

Trifolium repens L. 4 0 1.000 original measurement

Trifolium repens L. 4 1 0.000 original measurement

Trifolium repens L. 4 5 0.000 original measurement

Trifolium repens L. 4 10 0.000 original measurement

Trifolium repens L. 4 30 0.000 original measurement

Trifolium repens L. 4 60 0.000 original measurement

Trifolium repens L. 4 120 0.000 original measurement

Trifolium repens L. 4 180 0.000 original measurement

Trifolium repens L. 4 360 0.000 original measurement

Trifolium repens L. 4 1440 0.000 original measurement

Veronica chamaedrys s.str. L. 1 0 1.000 original measurement

Veronica chamaedrys s.str. L. 1 1 0.125 original measurement

Veronica chamaedrys s.str. L. 1 5 0.075 original measurement

Veronica chamaedrys s.str. L. 1 10 0.063 original measurement

Veronica chamaedrys s.str. L. 1 30 0.050 original measurement

Veronica chamaedrys s.str. L. 1 60 0.038 original measurement

Veronica chamaedrys s.str. L. 1 120 0.025 original measurement

Veronica chamaedrys s.str. L. 1 180 0.025 original measurement

Veronica chamaedrys s.str. L. 1 360 0.013 original measurement

Veronica chamaedrys s.str. L. 1 1440 0.000 original measurement

Veronica chamaedrys s.str. L. 2 0 1.000 original measurement

Veronica chamaedrys s.str. L. 2 1 0.333 original measurement

Veronica chamaedrys s.str. L. 2 5 0.267 original measurement

Veronica chamaedrys s.str. L. 2 10 0.244 original measurement

Veronica chamaedrys s.str. L. 2 30 0.211 original measurement

Veronica chamaedrys s.str. L. 2 60 0.178 original measurement

Veronica chamaedrys s.str. L. 2 120 0.156 original measurement

Veronica chamaedrys s.str. L. 2 180 0.044 original measurement

Veronica chamaedrys s.str. L. 2 360 0.011 original measurement

Veronica chamaedrys s.str. L. 2 1440 0.000 original measurement

Veronica chamaedrys s.str. L. 3 0 1.000 original measurement

Veronica chamaedrys s.str. L. 3 1 0.195 original measurement

Veronica chamaedrys s.str. L. 3 5 0.130 original measurement

Veronica chamaedrys s.str. L. 3 10 0.065 original measurement

Veronica chamaedrys s.str. L. 3 30 0.065 original measurement

Veronica chamaedrys s.str. L. 3 60 0.065 original measurement

Veronica chamaedrys s.str. L. 3 120 0.065 original measurement

Veronica chamaedrys s.str. L. 3 180 0.026 original measurement

Veronica chamaedrys s.str. L. 3 360 0.000 original measurement

Veronica chamaedrys s.str. L. 3 1440 0.000 original measurement

Veronica chamaedrys s.str. L. 4 0 1.000 original measurement

Veronica chamaedrys s.str. L. 4 1 0.103 original measurement

Veronica chamaedrys s.str. L. 4 5 0.103 original measurement

Veronica chamaedrys s.str. L. 4 10 0.103 original measurement

Veronica chamaedrys s.str. L. 4 30 0.103 original measurement

Veronica chamaedrys s.str. L. 4 60 0.052 original measurement

Veronica chamaedrys s.str. L. 4 120 0.017 original measurement

Veronica chamaedrys s.str. L. 4 180 0.017 original measurement

Veronica chamaedrys s.str. L. 4 360 0.000 original measurement

Veronica chamaedrys s.str. L. 4 1440 0.000 original measurement

Veronica officinalis L. 1 0 1.000 original measurement

Veronica officinalis L. 1 1 0.301 original measurement

Veronica officinalis L. 1 5 0.193 original measurement

Veronica officinalis L. 1 10 0.133 original measurement

Veronica officinalis L. 1 30 0.108 original measurement

Veronica officinalis L. 1 60 0.096 original measurement

Veronica officinalis L. 1 120 0.048 original measurement

Veronica officinalis L. 1 180 0.000 original measurement

Veronica officinalis L. 2 0 1.000 original measurement

Veronica officinalis L. 2 1 0.326 original measurement

Veronica officinalis L. 2 5 0.174 original measurement

Veronica officinalis L. 2 10 0.128 original measurement

Veronica officinalis L. 2 30 0.081 original measurement

Veronica officinalis L. 2 60 0.070 original measurement

Veronica officinalis L. 2 120 0.058 original measurement

Veronica officinalis L. 2 180 0.012 original measurement

Veronica officinalis L. 2 360 0.000 original measurement

Veronica officinalis L. 2 1440 0.000 original measurement

Veronica officinalis L. 3 0 1.000 original measurement

Veronica officinalis L. 3 1 0.209 original measurement

Veronica officinalis L. 3 5 0.149 original measurement

Veronica officinalis L. 3 10 0.119 original measurement

Veronica officinalis L. 3 30 0.090 original measurement

Veronica officinalis L. 3 60 0.090 original measurement

Veronica officinalis L. 3 120 0.045 original measurement

Veronica officinalis L. 3 180 0.045 original measurement

Veronica officinalis L. 3 360 0.015 original measurement

Veronica officinalis L. 3 1440 0.000 original measurement

Veronica officinalis L. 4 0 1.000 original measurement

Veronica officinalis L. 4 1 0.355 original measurement

Veronica officinalis L. 4 5 0.263 original measurement

Veronica officinalis L. 4 10 0.250 original measurement

Veronica officinalis L. 4 30 0.237 original measurement

Veronica officinalis L. 4 60 0.211 original measurement

Veronica officinalis L. 4 120 0.132 original measurement

Veronica officinalis L. 4 180 0.066 original measurement

Veronica officinalis L. 4 360 0.000 original measurement

Veronica officinalis L. 4 1440 0.000 original measurement

References for table S9:

"Lutz, M. (2004). Untersuchungen zum Ausbreitungspotential der Pflanzenarten"

"des Muttgletschervorfeldes (Wallis, Schweiz). Exam thesis (Staatsexamensarbeit) unpublished."

"Institute for Botany, University of Regensburg."

"Pirzer, S. (2007). Ausbreitungsbiologische Untersuchungen an mitteleuropaeischen"

"Suessgraeserarten. Exam thesis (Staatsexamensarbeit) unpublished. Institute for Botany,"

"University of Regensburg."
